# Simulation of Foot-and-Mouth Disease Spread and Effects of Mitigation Strategies to Support Veterinary Contingency Planning in Denmark

**DOI:** 10.3390/pathogens12030435

**Published:** 2023-03-09

**Authors:** Beate Conrady, Sten Mortensen, Søren Saxmose Nielsen, Hans Houe, Francisco Fernando Calvo-Artavia, Johanne Ellis-Iversen, Anette Boklund

**Affiliations:** 1 Department of Veterinary and Animal Sciences, University of Copenhagen, 1870 Frederiksberg C, Denmark; 2 Danish Veterinary and Food Administration, 2600 Glostrup, Denmark

**Keywords:** control strategies, economic impact, EuFMDiS, FMD, modelling

## Abstract

To forge a path towards livestock disease emergency preparedness in Denmark, 15 different strategies to mitigate foot-and-mouth disease (FMD) were examined by modelling epidemics initiated in cattle, pig or small ruminant herds across various production systems located in four different Danish regions (Scenario 1), or in one specific livestock production system within each of the three species geographically distributed throughout Denmark (Scenario 2). When additional mitigation strategies were implemented on top of basic control strategies in the European foot-and-mouth disease spread model (EuFMDiS), no significant benefits were predicted in terms of the number of infected farms, the epidemic control duration, and the total economic cost. Further, the model results indicated that the choice of index herd, the resources for outbreak control, and the detection time of FMD significantly influenced the course of an epidemic. The present study results emphasise the importance of basic mitigation strategies, including an effective back-and-forward traceability system, adequate resources for outbreak response, and a high level of awareness among farmers and veterinarians concerning the detection and reporting of FMD at an early stage of an outbreak for FMD control in Denmark.

## 1. Introduction

The spread and control of foot-and-mouth disease (FMD) is complex, with: (i) seven known immunologically distinct serotypes (O, A, C, Asia 1, and Southern African Territories 1–3), (ii) multiple transmission pathways, and (iii) several host species, such as cattle, pigs, and small ruminants [1,2,3,4]. This complexity is exacerbated by demographic and environmental heterogeneity, such as livestock density, frequencies of livestock movements, herd size, farm biosecurity standards, and livestock production systems. Livestock disease spread models can help to unpack this complexity in a close-knit multinational context [3,4] and aid in setting up a contingency plan for FMD preparedness by veterinary authorities.

National disease managers are faced with several logistical, economical, and societal challenges when responding to outbreaks of highly contagious diseases such as FMD. These challenges include which mitigation strategies to implement in the face of: (i) spatiotemporal heterogeneity of outbreaks, (ii) the effectiveness and efficiency of strategies to control the disease spread, (iii) available resources to manage outbreaks, and iv) potential losses due to international trade restrictions [3]. There is a growing interest in avoiding large-scale culling of infected or vaccinated animals [5] due to issues related to animal welfare, ethical, and sustainability goals [6,7]. If vaccinated animals are kept in the population until normal slaughter age, this adds complexity to the process of demonstrating the absence of FMD virus circulation in the livestock population and in regaining FMD-free status [8]. For countries with a large export-focused livestock industry, such as Denmark (N.B. the monetary value of intra-community traded (i.e., into EU countries) and exported (i.e., into non-EU countries) livestock and livestock products represents approximately 91.7% of the national monetary livestock outputs (EUR 6.75 billion) of live cattle, pig, and small ruminants and livestock products in Denmark in 2020. In 2020, agriculture, forestry, and fishing accounted for approximately 1.2% of Denmark’s gross value altogether [9,10,11]), it is a priority to regain FMD-free status as early as possible after an outbreak. As Denmark has not had an FMD case since 1983, we used the European foot-and-mouth disease spread (EuFMDiS) modelling framework to simulate epidemics in various regions, species, and livestock production systems in Denmark. The present study compares the epidemiological and economic effectiveness of varying mitigation strategies by comparing the predictions of: (i) number of infected, culled, and vaccinated farms; (ii) epidemic control duration (i.e., period between initial detection of first FMD-infected case and last culling of infected animal, including the completion of all control operational activities such as disinfection of farms, surveillance, vaccination); (iii) end day of post-outbreak management activities (i.e., the period of completion of all activities to regain FMD-free status, including surveillance in previously infected or vaccinated areas and culling, as well as disposal of vaccinated animals, if relevant); (iv) number of clinically inspected herds and number of performed laboratory tests of (non)-vaccinated herds; (v) direct costs of outbreak response, including: (a) operational control activities (i.e., surveillance, culling, disposal, cleaning, disinfection, vaccination, and compensation), (b) post-outbreak management activities (i.e., surveillance, culling, disposal, as well as compensation of vaccinated animals), and (c) production losses of farms due to business interruption; and finally, vi) indirect costs due to market disruptions of international trade. The present study aims to support veterinary authorities in terms of an effective outbreak response to FMD in Denmark by considering different mitigation strategies and introducing scenarios stratified by livestock species, geographical locations, and livestock production systems, as well as different detection periods and various national resources available for an outbreak response.

## 2. Materials and Methods

### 2.1. Epidemiological Model

For the simulation of the spread, detection, and control of FMD, the EuFMDiS model was used [3,12,13]. The Danish livestock population, including the species cattle, pig, and small ruminants (sheep and goats), was divided into different herd types—also defined as the livestock production systems—based on their production system, herd size, specific pathogen-free (SPF) status (N.B. SPF herds are regularly checked for the absence of several pathogens according to the health declaration system and follow a certain set of rules regarding biosecurity, including investments in changing farm facilities [14]. The SPF system is implemented in approximately 40% of all Danish pig herds), and number of on and off movements. The herd is the epidemiological unit and covers a group of animals of the same species in the same type of production system at the same geographical location. A farm may have one or more herds, and if a farm were to include several herds, each herd would be treated as a separate herd in the spread model. A herd has a dynamic set of descriptive attributes, such as infection status and vaccination states, while the unit of interest for FMD control is the farm. In total, 14.90 million livestock in 33,329 Danish herds (n = 28,748 farms) susceptible to FMD were incorporated in the model (Table 1). Farms located on the island of Bornholm in the Baltic Sea (Figure S1) were excluded from the model (i.e., from the total number of FMD-susceptible livestock herds, 2.0% pig herds, 0.9% cattle herds, and 1.9% small ruminant herds were excluded). Consequently, the model did not cover 0.02% of total livestock movements (i.e., movements between herds on Bornholm and the rest of Denmark).

The epidemiological model combines the following: (i) a susceptible-exposed-infectious-recovered-deceased-clinical (SEIRDC) (N.B. either susceptible (S) and then exposed (E) animals become infectious (I) and then transition to either recovered (R) (via natural immunity or vaccination) or dead (D) (although mortality rates are usually low, except in very young animals) [15]. A proportion of recovered cattle and sheep, except pigs, may continue to excrete the virus after they have recovered [3,4]. These animals are referred to as carriers [16]. Another option is that susceptible (S) and afterwards exposed (E) livestock either develop clinical disease (C) or are clinically inapparent [3]) compartmental, deterministic equation-based modelling technique to represent within-herd spread, (ii) a stochastic, spatial agent-based modelling techniques to simulate the spatiotemporal spread of disease between herds in daily time steps through multiple discrete transmission pathways [3], (iii) mitigating strategies, combined in a basic control strategy and/or additional strategies on top of the basic strategies, and (iv) national resources to ascertain and prove FMD-free status. FMD in herds is detected either through passive surveillance of clinical signs, such as by farmer awareness, and subsequently by a confirmation of suspected animals such as through laboratory tests (particularly applied for small ruminants, as FMD may not always be apparent clinically) and/or through active surveillance visits by veterinarians. Each herd in the model has a system of ordinary differential equations that model the herd’s infection and serological and clinical prevalence over time [3,4,12,13]. The proportion of infectious animals within the herd determines the likelihood of contact from a source herd leading to an infection in destination herds. Table S1 lists various key within-herd parameter settings used in this study.

The model considers the spread between herds in daily time steps through direct contacts, market/saleyard spread (mainly applicable for cattle herds in Denmark), indirect contacts, airborne spread, and local spread. Direct contacts can occur through the relocation of live animals between farms, from farms to markets and vice versa (referred as market/saleyard spread), and/or from farms to abattoirs, assembly centres, and other EU and non-EU countries (the last three trade activities are considered as “dead ends” of national direct disease transmission). Based on data from the Danish Central Husbandry Register (CHR), direct contacts between farms were described by the probability of livestock movements between different herd types (Figure S2); movement distance between herd types in km (Euclidean distance); seasonal movement patterns; the average number of livestock consignments moving off and on per herd type; and the size of consignment (i.e., number of transported animals). Based on the same source of data, indirect contacts through vehicles, e.g., the probability of the pickup of animals for slaughter and carcasses for rendering were calculated per herd type. Furthermore, the number of contacts, such as via veterinarians, milk tankers, feed delivery vehicles, artificial insemination technicians, and equipment for different herd types was estimated based on production systems and herd size, along with on estimates by Boklund et al. [17].

The airborne pathway function in the EuFMDiS model investigates for each simulation day whether weather conditions from 43 weather stations located in Denmark (Figure S1) are suitable for the spread of the virus through air beyond distances of 3 km [18,19]. As an infected pig can generate up to 400 million infectious doses per day (3000 times the doses of FMD virus particles excreted by ruminants [20]), only pigs were considered being a source for airborne transmission of FMD virus in the model. In contrast, pigs are considered quite resistant to airborne infection compared to ruminants (N.B. virus production is measured in tissue culture infectious dose of 50 units (TCID_50_), i.e., one TCID_50_ is the amount of virus that will infect 50% of exposed tissue cultures and is assumed to be directly proportional to the number of infectious virus particles present in a sample [21]. The threshold of virus concentration needed to become infected is much higher for pigs (=7.70 TCID_50_/m^3^) compared to cattle (=0.06 TCID_50_/m^3^), and small ruminants (=1.11 TCID_50_/m^3^) [22]). Besides weather conditions (N.B. appropriate weather conditions are constant wind direction from infected to susceptible herds, wind speed of five metres per second, high atmospheric stability, no precipitation, and relative humidity greater than 55% [18,19]), the probability of airborne transmission of FMD virus particles is influenced by the number of infected pigs in the source herd, virus plume concentration, distance from the susceptible herd to infected pig herds, susceptible herd species, and herd size (see Garner et al. [19] for additional details). Additionally, the model includes local spread within a 3 km radius as a spatial kernel approach to stochastically simulate the short-range transmission of FMD virus from an infected herd to neighbouring susceptible herds through aggregated spread mechanisms, such as unrecognised direct and indirect spread, as well as airborne spread pathways.

If farms practice high levels of external biosecurity, we assumed a lower probability of infection through the local spread and indirect contact pathways. Biosecurity weighting was assigned for each livestock herd type in the model according to the expert ranking (i.e., range 0 = low to 10 = high external biosecurity) by the Danish livestock industry and experts from the University of Copenhagen (n = 14 experts). For pig farms, nucleus pig herds were considered to have the highest levels of biosecurity (rank on average 10), while SPF farms were considered on average to have a rank of 8.2, and pig farms without SPF status were considered to have an average biosecurity rank of 6.2. For cattle and small ruminants, the rank was 4.2 and 5.0 on average, respectively. In this context, we assumed a linear relationship between biosecurity score and risk. For instance, herds with a score of > 6 are at a reduced risk of becoming infected through local and indirect contacts relative to the rest of the population. However, the ordinary differential equations system provides an updated SEIRDC compartment for each herd in case of infection through one of the five described transmission pathways and/or implemented mitigation strategies, such as the culling of infected animals or vaccination from that point in time onward [3,4].

### 2.2. Outbreak Scenarios and Mitigation Strategies

**Figure 1:**
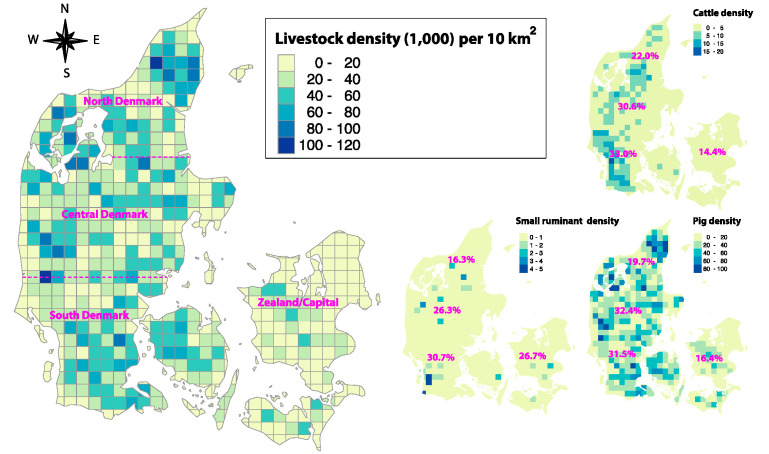
Livestock density map (n = 1000 livestock) per 10 km^2^ stratified by North Denmark, Central Denmark, Southern Denmark and Zealand/Capital (the left side of the figure represents the four regions of Scenario 1) with cattle, small ruminant, and pig density per 10 km^2^ (n = 1000) shown in maps on the right side of the figure. The percentages shown on the three maps to the right indicate the distribution of farms in North Denmark, Central Denmark, Southern Denmark, and Zealand/Capital.

We modelled an introduction of a type O pan-Asian strain in one of the regions: North Denmark, Central Denmark, South Denmark, or Zealand/Capital on 29 September 2021 (Figure 1). We randomly selected per region the following: (a) 1000 cattle index herds, (b) 1000 pig index herds, and (c) 1000 small ruminant index herds (Scenario 1) where the epidemic was initiated, which led to a total of 12 combinations (4 regions and 3 species). These index herds were chosen to cover all production systems within the associated species. We also selected one specific livestock production system per species as index herds independent of geographical regions, i.e.: (d) 1000 large commercial dairy herds; (e) 1000 large-scale commercial weaner pig herds, without SPF; and (f) 1000 small ruminant herds (Scenario 2, i.e., 1 country and 3 species). Further, Scenario 1 incorporated 1000 index herds per species, including hobby farms and livestock herds without any direct outgoing movements to other livestock farms, while in Scenario 2, the epidemic was always initiated in herd types with large numbers of livestock movements. Each of the 15 combinations (i.e., 12 combinations in Scenario 1 and three combinations in Scenario 2) was run for 1000 iterations (i.e., one repeat for each of the 1000 herds selected index herds), whereas in the sensitivity analysis, 10,000 runs were performed (see Section 2.4: Sensitivity analysis and model outcomes). In each iteration a different herd was chosen to consider the production variation between herds per species (Table 1). Each simulation was run until the disease/infection was eradicated (referred to control phase).

The model initially ran without any mitigation strategies until the end of a 21-day period (N.B. the 21-day period to detect the first FMD in the population was derived from other studies [24,25]) (defined as the silent spread phase) when the first FMD-outbreak was detected by the owners and was reported to the veterinary authorities. From the day of first detection onwards, the onset of basic control strategies were modelled (Table 2), including: a three-day standstill period for livestock movements at the national level (i.e., day 21–24); an establishment of a 3 km protection zone (PZ) and a 10 km surveillance zone (SZ) around each infected herd, with restrictions on livestock movements between herds, including animal products (illegal movements of animals and animal products were modelled as effects of movement restrictions not reaching 100% (Table 2)); and the culling and disposal of confirmed infected cases, which includes the cleaning and disinfection of infected holdings. Herds delivering or receiving animals to or from infected herds were assumed to be visited and/or tested, based on backward and forward tracing of contacts onto and off of infected herds within a 14-day trace window. Positively confirmed infected farms were depopulated and disinfected.

In addition to the basic mitigation strategies, 14 additional mitigation strategies were investigated and developed in consultation with the Danish Veterinary and Food Administration (Table 2). These additional mitigation strategies include depopulation (larger PZs and SZs), different vaccination campaigns, and both suppressive (inside PZs: vaccination to removal of animals) and protective ring vaccination (outside PZs: vaccination to retention of animals). Additional mitigation strategies were triggered once, e.g., a specific number of pending culling or infected herds were reached (Table 2 describes trigger functions), as we assumed that the veterinary authorities would start to implement additional control strategies, depending on the course of an epidemic and availability of resources.

The model considers that mitigation strategies are dynamically constrained by the following: (i) available national resources (i.e., surveillance, testing capacity, tracing, culling and disposal, cleaning, disinfection, and vaccination activities) due to a shortage of resources that can severely hamper the outbreak response, (ii) compliance with movement restrictions, (iii) accuracy of farmers reporting suspected cases, and (iv) the efficiency of the national tracing system (Table 3, [8]). For instance, depending on the number of infected farms, the model randomly generates false-positive reports of infected herds by owners (e.g., livestock showing clinical signs but not actually infected with FMD based on test results) with a predefined weight distribution function within PZs, SZs, and free zones (FZs; see Table 2). The clarification of suspected cases with associated resources in the model is prioritised for FZs, followed by SZs and PZs, both of which cover more realistic outbreak situations regarding consumed surveillance resources during epidemics. The EuFMDiS model tracks the availability and allocation of national resources (Table 3) and provides feedback on whether resources to perform control activities are constrained [3]. In case resources are limited to performing outbreak responses, such as culling of infected herds during outbreaks, the associated field operation is queued until the day the resources become available. In the EuFMDiS model, prioritisation of resources differs per operational activity. For instance, operational activities to farms are prioritised based on holding classification, herd/species priority, herd size, time in queue, and proximity to an infected farm. For example, a contact herd in FZs is assigned a higher surveillance visit priority compared to a contact farm in PZs. If several farms have the same priority, prioritisation is carried out based on how long a farm has been waiting for a surveillance visit, while farms awaiting culling are prioritised based on farm classification (i.e. infected farm > ring cull farm ≥ contact farm (direct contact) > suspect farm > trace farm (indirect contact) ≥ protection zone farm ≥ surveillance zone farm), species (i.e. pigs > cattle > sheep), and herd size (i.e. larger > smaller) in chronological order.

The model runs until the absence of virus circulation can be demonstrated and disease-free status can be regained (referred to as post-outbreak management), and subsequently includes clinical inspection and serological testing in the previously infected or vaccinated areas in order to identify past or present acute or persistent and/or sub-clinical infections (Table S2). If protective vaccination is applied as part of an outbreak response, more surveillance is necessary to differentiate between vaccinated and residually infected animals, for example, by detecting antibodies to non-structural proteins of the FMD virus [8]. Table S2 lists the sampling regimes of the post-outbreak management used in the present study, while Bradhurst et al. [4] and Garner et al. [8] provide a detailed description of a post-outbreak module in the EuFMDiS model, including the modelling of herds tested false-positive and false-negative.

### 2.3. Economic Model

The total economic impact of an FMD outbreak consists of direct and indirect costs. The former takes into account the following: (i) human resources (Table 3), equipment, facilities, and consumables for operational activities (i.e., clinical inspection, culling and disposal, cleaning and disinfection, and vaccination), compensation for farmers as well as control centre operations costs (defined as control operation costs) and (ii) post-outbreak management costs, including, e.g., surveillance visits, laboratory tests and follow-up costs for confirmation tests of already tested herds, and the culling, disposal, and compensation of vaccinated animals. Additionally, the present study considers contribution margin calculations (i.e., the difference between the total revenue and total variable costs at farm level) (Table 3) to estimate: (iii) production losses due to business interruptions for farmers in infected and vaccinated areas [7,30,31]. This includes empty stables caused by the culling of animals from the culling period until the last control day, and by using suppressive vaccination from the start day of vaccination until the last day of post-outbreak management activities. Table 3 lists selected epidemiological and economic parameters used in the model.

The indirect costs include losses due to national trade bans, intra-community movement restrictions on livestock and livestock products to the EU, and trade restrictions imposed by non-EU countries. Considering the Terrestrial Animal Health Code from the World Organisation for Animal Health (WOAH, formerly, Office International des Epizooties (OIE)) [32], a three-month waiting period for livestock trade following the culling of the last infected animal or vaccinated animal (suppressive vaccination), respectively, and a six-month waiting period under vaccination-to-retain strategy (protective vaccination) will be implemented before a country is declared as free from FMD. The same trade ban periods were assumed for animal products traded into non-EU countries and for farms in PZs and SZs with animal product consignments in EU countries (referred to as Calculation Approach 1), even though trade with animal and animal products to EU can resume as soon as the PZs and SZs are lifted in case of a non-vaccination policy [33].

Analysis of trade data from historical FMD outbreaks from various countries indicated that using the defined trade ban period based on the WOAH guidelines can underestimate the time to trade recovery (i.e., when trade value returns to a moving average for trade value in the months immediately prior to the outbreak [34]). In terms of the estimation of the full trade recovery period, Seitzinger et al. [34] proposed adding four months plus 3.8 months for each 30-day epidemic control (referred to as Calculation Approach 2). We used their suggested trade recovery period for all livestock and livestock product consignments delivered to non-EU countries and for all farms located in PZs and SZs delivering to the EU if a non-vaccination or suppressive vaccination strategy was applied. Nevertheless, we adjusted the associated total trade losses down- or upwards by using the following path of trade recovery for Denmark: we assumed that 12% of the value of the consignments that were previously intended for non-EU countries would be reallocated to the EU marked by considering the current oversupply, price decrease, and increasing storage of meat on the EU market [35]. In FZs, we assumed trade bans on live animals into the EU until the end of the control period. In terms of using a protective vaccination strategy, no good estimations for trade ban periods were available from historical data. Thus, the present study used the suggested trade recovery period by Seitzinger et al. [34] for all consignments into EU and non-EU countries, and the period when all post-outbreak management activities were completed, if a protective vaccination strategy were used. The economic model used control duration and post-outbreak management duration, as well as data on the simulated number and type of farms and herds in PZs, SZs, and FZs, as well as changes in farms between zones during the epidemic. The value of trade losses stratified by EU and non-EU countries per day was estimated proportionally to the regional production of the livestock farms in these zones [7]. Both Calculation Approaches 1 and 2 were used to estimate the indirect costs for Denmark and in both cases, we assumed that Danish imports and domestic consumption were unchanged during the outbreak period.

### 2.4. Sensitivity Analysis and Model Outcomes

The mitigation strategy with the lowest epidemic size and/or total economic costs was considered as the optimal control strategy for Denmark. The impact of potentially uncertain data inputs was investigated in terms of time until the first farm was detected (varied from day 21 (initial) to day 14 and day 28, respectively), and the available resources for outbreak response by doubling the currently estimated human resources, such as for surveillance, stamping out, disposal, and vaccination (referred as optimistic values in Table 3). We also analysed whether the number of infected farms and total economic losses were sensitive in terms of the number of iterations by comparing the model outcomes with each other for 1000 (i.e., one repeat of each of the 1000 index herds per species) vs. 10,000 iterations (i.e., 10 repeats of each of the 1000 seed herds).

For statistical analyses of the epidemiological and economic results from the 15 strategies and the sensitivity analyses, the Wilcoxon rank sum test for paired observations was used, while the unpaired version was used for the comparison of Scenarios 1 and 2, as the model outcomes were not normally distributed. The results in the present study were expressed as medians with the corresponding 5th and 95th percentiles. The significance level was set to *p* < 0.05. The statistical analyses were conducted with the open-source statistical computing environment R version 4.1.2 [36].

The selected epidemiological outcomes of the model presented in this study are:

▪ Number of farms infected
▪ Number of farms depopulated
▪ Number of farms vaccinated (when used)
▪ Epidemic control duration (days)
▪ End of post-outbreak management (days)
▪ Number of herds clinically inspected
▪ Number of vaccinated and unvaccinated herds tested
▪ Number of (vaccinated) animals culled
▪ Mean spatial distribution of disease spread (km)
▪ Proportion of the involved transmission pathways per 1000 simulations (%)
▪ How frequently enforcement and trigger setting (Table 2) initiated mitigation strategies

The selected economic outcomes of the model presented in this study are:

▪ Total operation costs for
  - Surveillance
  - Culling
  - Disposal
  - Disinfection
  - Vaccination
  - Control centres
  - Compensation
▪ Total production losses
▪ Total post-outbreak management costs for
  - Surveillance
  - Culling (applicable for suppressive vaccination)
  - Disposal (applicable for suppressive vaccination)
  - Compensation (applicable for suppressive vaccination)
▪ Total economic costs (direct and indirect) using Calculation Approaches 1 (WOAH) and 2 [34]
▪ Days out of market
▪ How often resource constraints occurred during simulation runs, stratified by Scenarios 1 and 2

## 3. Results

The results mainly compared Scenario 1 and Scenario 2, where Scenario 1 incorporated 1000 index herds per species, including hobby farms and livestock herds without any direct outgoing movements to other livestock farms (Figure S2); Scenario 2 considered the initiation of the epidemics in herd with a large number of livestock movements.

The model results indicated non-significant epidemiological or economic benefits in terms of reduced numbers of infected farms and total economic losses due to the implementation of additional mitigation strategies on top of the basic control strategies. This conclusion is independent of: (a) geographical region and species-group (Scenario 1) and specific livestock production systems in which the epidemic was initiated (Scenario 2), (b) how the indirect costs were calculated, (c) whether the resources for outbreak response were doubled, and d) whether the first infected herd was detected seven days earlier (i.e., on day 14 or instead on day 21). In the period before the first detection and implementation of mitigation strategies, the main driver for the FMD spread in Denmark was predicted to be direct contacts, on average with a proportion of 47.8%. After first detection and implementation of mitigation strategies, the importance of transmission pathways shifts to indirect (40.3%), local (27.6%) and direct contacts, airborne spread and market/saleyard spread (32.1%).

### 3.1. Epidemiological Model Outcomes

**Figure 2:**
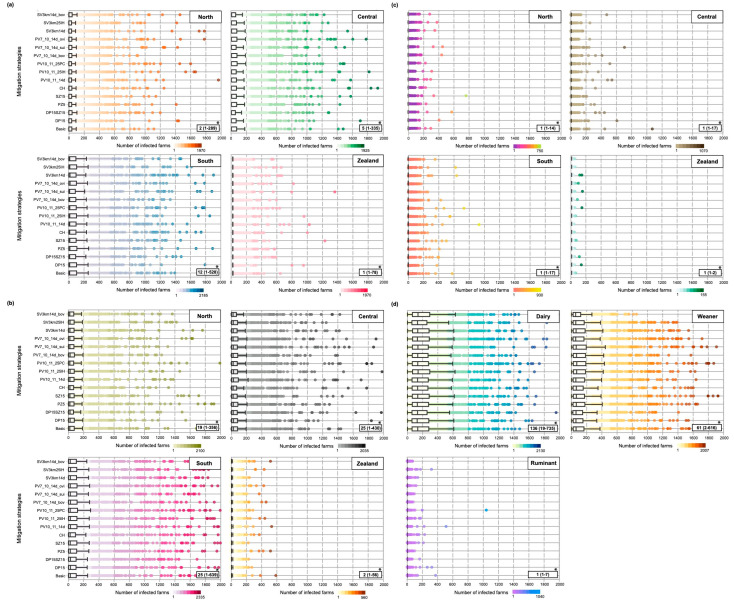
Box and whisker plot of the number of infected farms for the Scenario if the epidemic was initiated in: (**a**) the cattle population, (**b**) pig population, and (**c**) small ruminant population, or (**d**) in specific livestock production types. Thus, (**a**–**c**) represent Scenario 1 and (**d**) shows Scenario 2. Table 2 lists the definition of abbreviations for mitigation strategies. * Median (5th and 95th percentiles) mitigation strategy. N.B. line in the boxes is the median value, the box limits are the 25th and 75th percentiles, and the whiskers are the minimum and maximum values of the box and whisker plots.

The median predicted number (5th and 95th percentiles) of infected farms in Scenario 1 ranged between 1–12 (1–528), 2–25 (1–639), and 1 (1–17) if the epidemic was initiated in cattle, pigs, and small ruminants, respectively, and by the implementation of basic mitigation strategies (Figure 2; Table S3). The largest infection area was predicted if an infection started in cattle and pig farms in South Denmark (46,266 km^2^ on average) and the smallest area was predicted if the epidemic started in Zealand/Capital in a small ruminant population (973 km^2^ on average).

A significantly higher number (*p* < 0.001) of infected farms was predicted in Scenario 2 compared to Scenario 1. For instance, if FMD virus was introduced in a large commercial dairy herd, on average, 27 times more infected farms were predicted, compared to an epidemic initiated across all cattle production systems. A smaller effect was predicted in pigs, where the epidemic size was on average 3.4 times higher in Scenario 2 compared to Scenario 1. The epidemic was self-limiting and burned out without spreading to other farms in 30.0% of the simulation iterations in Scenario 1, while in Scenario 2, only 3.1% of them did not spread beyond the index herds. The number of infected farms in Scenario 2 corresponds to 0.47% of the number of FMD-susceptible farms in Denmark on average (Figure 2; Table S3).

**Figure 3:**
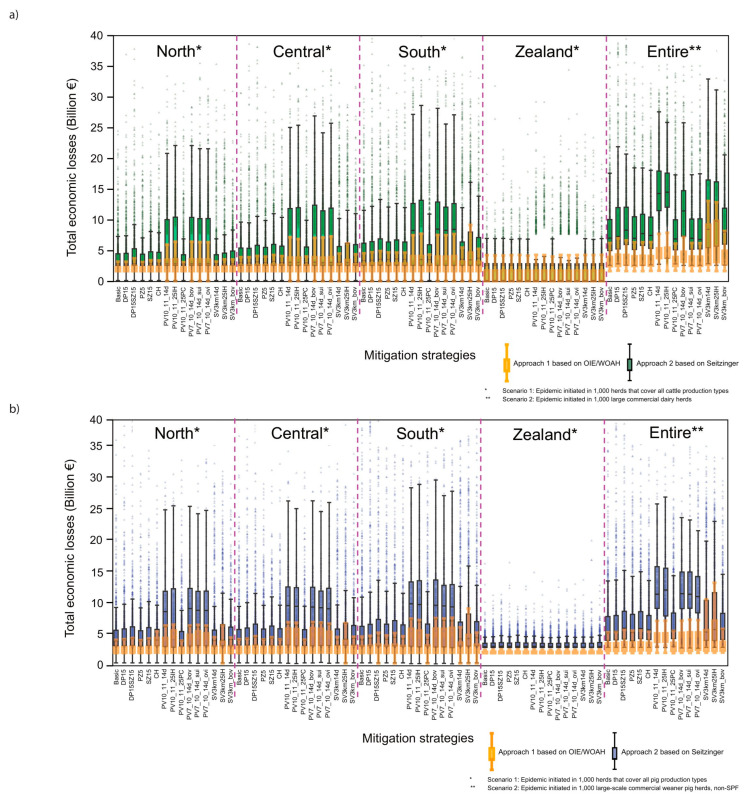
Total economic losses if the epidemic was initiated in: (**a**) the cattle population and (**b**) the pig population, stratified by Scenarios 1 (*) and 2 (**) and Calculation Approaches 1 (box plot coloured in yellow) and 2 (box plot coloured in green or blue). The cost of an epidemic initiated in small ruminants is not shown, since only a single horizontal line would be visible, as the median economic losses (EUR 2.48 billion in Scenario 1 and EUR 2.65 billion in Scenario 2) do not differ. Table 2 lists the definition of abbreviations for mitigation strategies.

A comparison of the model outcomes for Scenario 2 indicated that epidemics initiated in large commercial dairy herds are characterised by a larger number of affected livestock herds, longer epidemic control, and post-outbreak management duration, consequently leading to larger total economic losses compared to epidemics initiated in weaner pig herds. For instance, on average, a 2.23-fold higher number of infected farms, 1.25- and 1.28-fold longer control and post-outbreak duration, and 1.68-fold higher costs occurred for epidemics initiated in dairy herds compared to weaner herds in Scenario 2. By comparing epidemics initiated in dairy herds and small ruminant herds, on average 134-fold higher number of infected farms, 2.46- and 3.01-fold longer control and post-outbreak duration, and 3.38-fold higher costs caused in Scenario for dairy herds; *p*-value < 0.001; Figure 2 and Figure 3). Table S3 provides a detailed overview of the epidemiological model outcomes, including the number of culled farms and animals, number of clinical inspected and tested herds stratified by Scenarios 1 and 2, and different mitigation strategies.

### 3.2. Economical Model Outcomes

The lowest median economic losses were predicted to be caused by an implementation of basic mitigation (EUR 2.5–4.7 billion for Scenarios 1 and 2, respectively), and the highest economic losses occurred when using protective vaccination strategies 14 days after outbreak detection (EUR 2.5–10.0 billion for Scenarios 1 and 2, respectively) (Figure 3; Table S4). The days out of mark were predicted to be 59 days longer in Scenario 2 compared to Scenario 1 by using Approach 1 and 93 days longer by using Calculation Approach 2. In general, the median indirect costs were 37% higher when using Calculation Approach 2 compared to Approach 1 (Figure 3). Independent of the approach, most of the total economic costs can be attributed to indirect losses. In Scenario 1, on average, 0.32% (up to 1.85% with suppressive vaccination), and in Scenario 2, on average, 2.83% (up to 36.49% with suppressive vaccination) are direct losses (Table S4 presents costs in detail).

**Figure 4:**
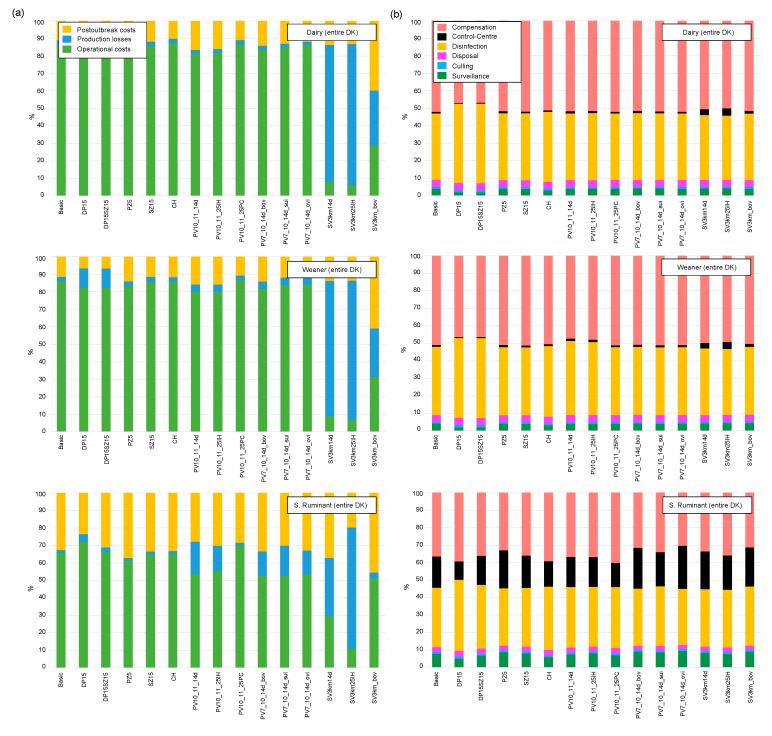
Distribution of direct costs; (**a**) Percentage distribution of the average direct costs stratified by operational control costs, production losses, and post-outbreak costs per mitigation strategies for Scenario 2; (**b**) detailed percentage distribution of the average costs within the control operation cost component, i.e., surveillance, culling, disposal, cleaning and disinfection, control centre costs, and compensation. Information for the other regions (Scenario 1) and other cost components are shown in Table S4.

Figure 4a shows the distribution of the predicted direct costs stratified by operational costs, production losses, and post-outbreak costs per mitigation strategies and species-group (Scenario 2). Figure 4b shows that the cleaning and disinfection activities and compensation payments for the farmers cover the highest share of the operational control costs. If the epidemic is initiated in small ruminants, the implementation and operation of national and local control centres covered a large proportion of the operational control costs as well. Table S4 provides a detailed distribution of the direct and indirect costs, stratified by Scenarios 1 and 2, and the various mitigation strategies used.

### 3.3. Sensitivity Analysis

**Figure 5:**
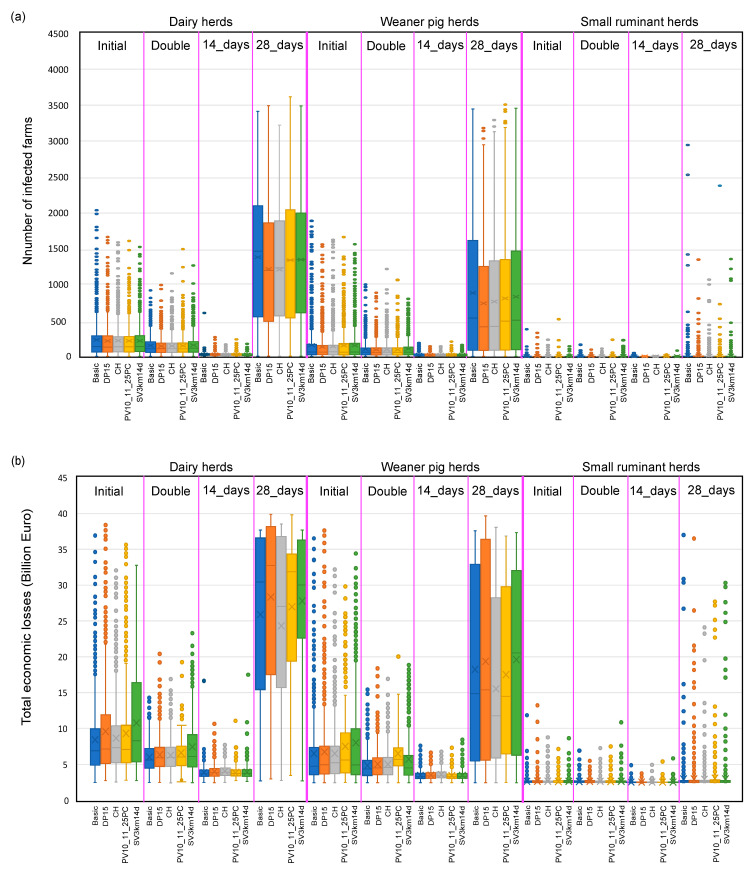
Box and whisker plot of the sensitivity analyses outcomes for some selected mitigation strategies: (**a**) number of infected farms and (**b**) total economic losses for the Scenario 2, stratified by an initiation of the epidemic in large dairy herds, large weaner pig herds, non-SPF, and small ruminant herds throughout Denmark. Resources are doubled (such as human resources min/max number of surveillance teams: 16/130; culling teams: 6/74; disposal teams: 8/68; decontamination teams: 8/82; vaccination teams: 14/144) compared to initial assumed resources (such as surveillance teams: 8/65; see further detail information in Table 3) and the detection day is decreased from 21 (initial) to 14 days and then increased to 28 days.

An inadequacy of resources to undertake a control programme was detected during the simulations that led to a 28-day delay on average between the last culling activity and last disease outbreak response activity, e.g., cleaning and disinfection. The sensitivity analysis suggested that increasing resources reduced the average number of infected farms and total economic losses by up to 15.6% and 15.4%, respectively. If the resources were doubled in Scenario 2, the numbers of infected farms and the total economic losses were reduced by 6.5% and 27.3% (simulations with initial resources: 0.3% and 3.6%, on average) for epidemics started in large commercial dairy herds, and 39.5–97.6% (simulations with initial resources: 5.8% and 18.9%, on average) for epidemics started in large commercial weaner pig herds without SPF of the simulation’s runs were scheduled surveillance visits and culling activities that could be completed on time, respectively. Further, the model outcomes were sensitive regarding the detection time (±7 days compared to the 21-day detection time). Decreasing or increasing the silent phase by seven days in Scenario 2 decreased or increased the predicted number of infected farms (or total economic losses) by 79% (42%) or 929% (400%) on average across all epidemic simulations initiated in large commercial dairy herds and large commercial weaner pig herds, without SPF, respectively. No significant changes were observed by variation of the detection time ±7 days, if the epidemic was initiated in small ruminant herds. Figure 5 shows the outcome of the sensitivity analysis for selected mitigation strategies in Scenario 2. The prolonged detection time from 21 to 28 days led to a shift in the optimal mitigation strategy in terms of minimal median number of infected farms from basic mitigation to pre-emptive depopulation of all susceptible herds within a 1 km radius around each infected herd, with a trigger after the confirmation of 15 infected herds (Figure 5a) and/or pre-emptive culling of dangerous contact herds to infected herds, based on tracing without any confirmations of the infection status (DP15 and CH; see Table 2 and Figure 5a,b), except in the Scenarios where the epidemic was initiated in small ruminant herds.

However, the pre-emptive depopulation of all susceptible herds predicted twice as many culled farms (up to four times more animals culled), an extension of the control duration of up to 50 days, and 1.16 times higher total economic losses occurred compared to the pre-emptive culling of dangerous contact herds (CH). Thus, from an epidemiological and economic point of view, the pre-emptive culling of dangerous contact herds to infected herds based on tracing without any confirmations of the infection status would be the most beneficial strategy in case of a late discovery of FMD, on average (resulted in 9.8% lower total economic losses compared to basic mitigation; across all simulations). The model outcomes did not differ statistically in terms of the number of performed iterations of 1000 vs. 10,000 (detailed results available upon request).

## 4. Discussion

This paper presents the results of a simulation study that investigated different mitigation strategies for FMD epidemics initiated in various species, regions, and production systems in Denmark. It is reassuring for veterinary authorities that in all simulations of Scenario 1, outbreaks were relatively small and readily controlled through basic mitigation activities and currently estimated available national resources; however, there is still a need for the improvement of investments. For instance, constraints were observed on performing surveillance, culling, and cleaning and disinfection activities during the simulations. One explanation for the quite similar number of infected farms between basic mitigation strategies and additional strategies (Table 2) is that the implementation of some mitigation strategies is linked to predefined trigger functions. For instance, only 28% of the simulations in Scenario 1 predicted the course of an epidemic to be larger than 15 infected herds that would enforce the implementation of depopulation in the model. Thus, we can conclude that basic mitigation activities may be the most appropriate outbreak response for smaller outbreaks as other mitigation strategies (including also strategies without any trigger functions; see Table 2), such as ring culling, depopulation of all direct contact herds, enlargement of PZ and SZ, or vaccination (Table S3), as additional mitigation strategies did not significantly reduce the outbreak size or epidemic duration, nor affect the total economic losses for Denmark (Figure 2 and Figure 3, Tables S3 and S4). This also implies an effective back- and forward tracing of direct and indirect contacts, the availability of estimated resources to manage the outbreak, and the reporting of suspicious cases in FZ, SZ, and PZ to identify infected herds within the basic mitigation strategies, as assumed in the model (see Table 2).

One explanation for the significantly higher number of predicted infected herds in Scenario 2 compared to Scenario 1 (Figure 2 and Figure 3) is the different herd sizes and trade patterns of the randomly selected 1000 index herds. While Scenario 1 incorporated all livestock production types per species, including hobby farms and livestock herds without any direct outgoing movements to other livestock farms (Figure S2), Scenario 2 considered herds with a large number of livestock movements. This influences the direct transmission probability of infection between farms. Consequently, the probability that the epidemic would not spread beyond the index herd is higher in Scenario 1 compared to Scenario 2 (see detail results in Section 3.1). Thus, the present study supports the model outcomes of other studies in terms of the course of the epidemic and control duration, and the control strategy of choice might be affected by epidemic initialisation in the chosen index herd [13,17,37,38,39,40]. Based on these results, we recommend investigating the epidemic course for each cattle production system individually in Denmark by using the EuFMDiS model, as the epidemiological and economic outcomes between Scenarios 1 and 2 varied much more for the epidemic initiated in the cattle population compared to the epidemic introduced into the pig population (Figure 2 and Figure 3, Tables S3 and S4), which might influence the choice of the optimal mitigation strategies from basic mitigation to other additional mitigation strategies.

Although it is difficult to directly compare our model outcomes with those from other studies due to different study and modelling designs, data inputs, scenarios, and assumptions, our findings are supported by other studies which report that additional mitigation strategies do not significantly reduce the number of infected herds across all three species, as shown in a previous Danish study by using another simulation model, referred to as DTU-DADS model [41], or in specific simulated regions in Austria by using the EuFMDiS model [7,31]. In contrast to our study, where 1000 cattle or small ruminant herds covering all production types were randomly selected in specific regions or for one specific production system throughout Denmark, Halasa et al. [41] chose 1000 cattle and small ruminant herds across all production types across Denmark. Despite this difference, the DTU-DADS model predicted, as in the presented study, a relatively small and comparable number of infected farms initiated in cattle and small ruminants (i.e. a median of 12 infected farms for epidemics initiated in cattle, independent of the region in Halasa et al.’s study [41], and a median of 1–12 infected farms in our study, depending on the region; a median of four infected farms for epidemics in small ruminants in the study by Halasa et al. [41], and a median of one infected farm in our study; epidemics initiated in pig herds differ between both studies in terms of the chosen production type and are not comparable). The tendency of the DTU-DADS model to predict smaller epidemics by initiating FMD in pig herds (there were 5 median infected farms, and in our study 2–25 in Scenario 1, depending on the region, and 61 median infected farms in Scenario 2) and smaller epidemic areas (5054 km^2^ [39] compared to 46,266 km^2^ in the present study; which might be explained by different approaches on how the convex hull was calculated) could be explained, apart from the chosen index herds, by the way in which the disease spread is modelled. In contrast to EuFMDiS, the DTU-DADS model does not include daily weather conditions for an airborne spread pathway module beyond a distance of 3 km [41], which might influence the epidemic size, control duration, and associated costs, as shown in other studies [17]. A limitation of the EuFMDiS model is that topographical conditions are not incorporated in the airborne spread pathway, such as mountain distributions which would cause a plume to deviate and thus reduce the transmission distance [7,42]. We assume that neglecting the topology does not affect the airborne spread pathway significantly in the present study, as for instance, the percentage of land area covered by mountains in Denmark is non-existent (0%) compared to other countries, such as Austria (74%) [43]. As less than 11% of the farms cover more than one species (see also [41]), direct movements between pig herds and cattle or small ruminant herds occur rarely in Denmark (Figure S2). Moreover, market/saleyard spread is mainly applicable for cattle herds in Denmark, whereby most of the infection between different domestic livestock species can be assigned to indirect, local, and airborne spread in Denmark. This is in contrast to other countries, such as the UK, where 56% of cattle farms also have small ruminants and a high record of movements between both species, including a high number of movements that went through livestock markets [44].

There are several additional dissimilarities between the models: (i) the EuFMDiS model incorporates external biosecurity strategies, which reduces the risk of a farm becoming infected through local and indirect transmission pathways; (ii) DTU-DADS categorises indirect contact in medium risk (e.g., for veterinarians, milk controllers) and low risk (e.g., animal feed and rendering trucks), while EuFMDiS does not distinguish between various risk levels for different types of indirect contacts, but distinguished the indirect contacts for different herd types similar to the DTU-DADS model; (iii) DTU-DADS assumes a quite constant effectiveness probability to trace animals; in the present study, we have considered that tracing effectiveness differs per species; (iv) some parameters stochastically incorporated in the model differ between both models (e.g. the probability of detecting diseases from clinical surveillance and testing); (v) EuFMDiS includes a detailed post-outbreak management plan that includes the false-negative and false-positive modelling of tested herds, while DTU-DADS does not do this, i.e., only testing of small ruminants before lifting of the zone (days 30–35) are incorporated in the DTU-DADS model; (vi) the definition of the epidemic control duration varied, while Halasa et al. [41] defined the period between the first detection and culling of the last infected herd, we have defined the end of the period by the completion of cleaning and the disinfection of the farms, and other studies provide no definition or other definitions [7,45,46,47,48]; (vii) EuFMDiS includes false reports by farmers during the epidemic, providing a more realistic outbreak situation regarding consumed resources during epidemics, hence causing more resource constraints and thus leading to a twice prolonged epidemic control duration compared to the DTU-DADS model, if the same definition of the epidemic control duration is used as by Halasa et al. [41]; and (viii) the trigger settings considered study periods and the economic inputs in terms of available national resources for an outbreak response, in addition to the prioritisation of these resources to perform operational activities which might also differ between the two models. These differences might explain the fact that, although the median number of infected farms was quite similar between both models, the median direct losses were 27.9% higher in the present study compared to Halasa et al. [41]. Nonetheless, as several studies show [7,17,31,38,39,40,41], indirect losses are the main driving force of total economic losses and were two times higher (Scenario 1) in the present study compared to a previous Danish study [41]. For instance, the Danish studies [17,41] assumed that 75% of the trade value previously allocated for non-EU countries would be placed on the local and EU market, while in the present study we assumed only a re-allocation of 12% on the EU market due to oversupply, price decrease, and increasing storage of meat on the EU market in the last two years [35]. In general, market behaviour in the event of an FMD outbreak and the reaction of various countries are difficult to predict, since they might not obey WOAH guidelines [40]. This is supported by Seitzinger et al.’s [34] analysis of historical trade data in countries formerly infected with FMD; their study indicates that WOAH waiting periods can underestimate trade losses, since the trade recovery period can be longer (as described in WOAH guidelines and shown in the present study by comparing Calculation Approach 1 and 2). In general, the calculated economic impact between studies is difficult to compare due to different data inputs and how the suggested trade recovery based on WOAH guidelines was down- or upwardly adjusted for livestock and/or individual livestock products. For instance, the allocation of the monetary trade value of FMD-susceptible livestock and associated animal products differed between the EU and non-EU countries over the study period between both studies (our study and Halasa et al. [41]). A 2.2 times higher monetary trade value of FMD-susceptible livestock and animal product consignments from Denmark to EU countries compared to non-EU countries was reported in 2018 [41], while in 2020, the export value to non-EU-countries was 1.11 times higher compared to EU countries [11] (Table 3). As EU and non-EU countries might differently manage the period of export bans of FMD-susceptible animal products in the event of an FMD outbreak, the different allocations of the trade value between EU and non-EU countries in both 2018 and 2020 might explain the huge difference in terms of indirect losses between both studies, in addition to the general higher exports of live animals and products in 2020 (EUR 21.6 Mio. per day) compared to 2018 (EUR 18.5 Mio. per day). All these differences might explain why Halasa et al. [41] identified basic mitigation strategies combined with ring depopulation in a 1 km radius from detected herds (with a standard or a 15 km surveillance zone) as the optimal mitigation strategy based on total economic losses (not in the epidemiological outcomes [41]). To address the potential limitation of economic dynamic changes and assumptions regarding market behaviours, we recommend not only choosing the optimal mitigation strategy based on total economic losses, but also on the epidemiological model outcomes itself, such as in the reduction of the number of infected and culled herds, outbreak duration, and size of affected areas.

For a disease manager to have confidence in simulation models, it is important that model limitations are recognised [19,49]. The model presented in this study depends on estimations and assumptions, e.g., in terms of the availability of resources for an outbreak response, and down- or upwards adjustments of trade losses, such as by assuming trading zones. These might influence the selection of the optimal mitigation strategy. For instance, besides the large epidemic size, resource constraints were the main reasons for the shift of the optimal control strategy from basic mitigation strategies to pre-emptive depopulation by the detection day 28 shown in the sensitivity analysis for Scenario 2 (Figure 5). Furthermore, rendering capacities for the destruction of affected livestock are not included in the EuFMDiS model for Denmark, which might be a limited resource in many countries during an outbreak by considering the ongoing increase of the herd sizes of livestock farms [41]. Finally, the total economic losses might be under- or overestimated by not considering the following: (a) 50% re-imbursements by the EU for the disposal, cleaning and disinfection of herds, destruction of animals, animal products, and contaminated feed [50]; (b) 48 h quarantine on all visitors in positive farms; (c) by assuming that Danish imports and domestic consumption were unchanged during the epidemic, which is quite unrealistic by considering exports bans and limited storage capacities for livestock products in Denmark; (d) direct losses might be underestimated by not considering production losses in zones (e.g., not collecting the raw milk from dairy farms) and economic shocks to other food and non-food industries such as wool production, biogas production; and (e) the model for Denmark is set up for the pan-Asian strain of the O serotype because the last outbreak in Denmark was caused by this serotype [51]. A consideration of other serotypes requires model adaptions in terms of the transmission characteristics, vaccine efficacy per species, time to seroconversion and seropositive duration in livestock species, and test sensitivity and specificity of the lab test.

However, in line with previous results from other models, our study demonstrates that, in spite of a few infected herds, an FMD outbreak can have a huge negative economic impact for Denmark (Figure 3). This is a clear indication for countries with a large livestock production and export-oriented livestock industry to be well-prepared to control infectious diseases such as FMD [8,41]. This includes guidelines on sampling during an outbreak and on post-outbreak management within the contingency planning to support regaining FMD-free status. In this context, future research is necessary to determine the best approach to regaining FMD-free status for Denmark, such as by incorporating new sampling techniques (e.g., bulk milk testing (BMT) of dairy herds [8,52]) in the EuFMDiS to investigate the benefits in terms of regaining FMD-free status earlier with these potentially faster-performing operational and post-outbreak surveillance activities, compared to traditional testing approaches (e.g., blood samples [8]). Additionally, seasonal effects of FMD spread should be investigated in terms of different weather conditions for the airborne dispersion of the FMD virus in detail, as in the present study, infections were initiated in autumn, the most appropriate season to transmit the virus through air [7,18]. Further, it is important to increase awareness among farmers and veterinarians concerning the detection and reporting of FMD at an early stage, as the detection time has a huge impact on the course of the epidemic, as shown in the present study. In contrast to the DTU-DADS model, the EuFMDiS includes a transboundary spread module and allows simulations beyond the national borders. Transboundary spread simulations with available national and EU resources will provide new knowledge to manage FAST diseases by exchanging resources across EU countries in outbreak situations, and set up a necessary core capacity for Europe. This requires a preparedness-index for FAST diseases at the national and EU level, stratified by different transmission pathways.

Although the present study results can provide valuable preparedness information for other countries with similar livestock and economic structures, the nature and extent of FMD outbreaks depend on many variables, including livestock and farm density, number of livestock movements, biosecurity practice, production systems, climate, pathogen specifics, effectiveness of tracing systems, animal health resources for outbreak response—which might influence the importance of individual transmission pathways—and the success of individually analysed mitigation strategies of countries. Thus, a specific model parameterisation and country-specific adaptation of the model are beneficial to obtain country-tailored model results for the contingency plan.

## Figures and Tables

**Table 1:** Herd types used in EuFMDiS model for FMD outbreak simulations in Denmark.

Herd Type ^1^	Number Of Herds	Mean Herd Size ^2^ (Min–Max)	Characteristics of Herd Types
Large dairy (commercial)	2846	294 (10–4231)	Deliver milk to the factory; cattle primarily kept to produce and sell milk (n > 10 heads)
Large beef (commercial)	676	175 (100–2686)	Cattle primarily kept to produce and sell meat (n ≥ 100 heads)
Heifer hotel	711	70 (1–1556)	Heifers sent from several farmers and then transported back when they begin producing milk
Mean cattle (commercial)	3678	28 (10–100)	Kept ≥10 female cattle heads but ≤100
Small cattle (commercial)	1056	13 (10–99)	Cattle primarily kept to produce and sell meat and/or milk on a smaller and local scale; kept <10 female cattle heads but <100
Small ruminants	305	85 (40–3717)	Small ruminants (sheep and/or goats) kept primarily to produce and sell meat, milk, and/or wool commercially (≥40 heads)
Large-scale fattening pig, non-SPF (commercial)	1610	1500 (100–18,200)	Pigs kept under intensive production system to be grown and sold for slaughter and meat production; no sows but finishers and optional weaners; not part of SPF system
Large-scale weaner pig herd, non-SPF (commercial)	89	3500 (400–22,000)	Pigs kept under intensive production system to be grown and sold for finisher production; no sows or finishers but weaners; not part of SPF system
Large-scale full-scale pig production herd, non-SPF (commercial)	283	2180 (102–19,600)	Full-scale pig production, from farrow to finisher; pigs kept under intensive production system to be grown and sold for slaughter and meat production; sows, finishers, and optional weaners; not part of SPF system
Large scale breeding pig, non-SPF (commercial)	72	1389 (110–7533)	Pigs kept under intensive production system for producing replacement pigs to be sold to other pig farms; sows, no finishers, and optional weaners; not part of SPF system
Large scale breeding pig, SPF (commercial)	290	1508 (100–14,500)	Pigs kept under intensive production system for producing replacement pigs to be sold to other pig farms; sows, no finishers but optional weaners; part of SPF system
Large-scale fattening pig herd, SPF (commercial)	789	2200 (10–19,600)	Pigs kept under intensive production system to be grown and sold for slaughter and meat production; no sows but finishers and optional weaners; part of SPF system
Large-scale weaner pig herd, SPF (commercial)	229	3800 (167–21,333)	Pigs kept under intensive production system to be grown and sold for finisher production; no sows or finishers but weaners; part of SPF system
Large-scale full-scale pig production herd, SPF (commercial)	821	2595 (120–16,800)	Full-scale pig production, from farrow to finisher; pigs kept under intensive production system to be grown and sold for slaughter and pig meat production; sows, finishers, and optional weaners; part of SPF System
Nucleus pig herd, SPF	201	2600 (100–15,300)	Pigs kept under intensive production system for producing replacement sows to be sold to sow holdings; highest level of biosecurity and part of SPF system
Hobby	6887	5 (1–97)	Small number of livestock kept primarily for own consumption (non-commercial) but with outgoing livestock movements to other farms; ≤10 cattle heads (all age groups) and/or <40 sheep and/or goat heads and/or <100 pig heads
Small ruminant herds without outgoing consignments	7112 ^3^	4 (1–2400)	No outgoing animal consignments (sheep and/or goats) to other farms but can receive consignments
Cattle herds without outgoing consignments	2534 ^4^	3 (1–341)	No outgoing animal consignments to other farms but can receive consignments
Pig herds without outgoing consignments	3140 ^5^	4 (1–12,500)	No outgoing animal consignments to other farms but can receive consignments
Total	33,329	-	Cattle herds (n = 16,033) with a population of 1.49 million heads, pig herds (n = 8043) with a population of 13.24 million heads, and small ruminant herds (n = 9254) with a population of 162,601 heads included in model

^1^ Population of FMD-susceptible livestock assigned to different herd types based on species, herd size, and production practices, SPF system and movement activities. ^2^ Herd size presented as median calculated for each herd based on monthly herd composition available in Denmark’s Central Husbandry Register. ^3^ 97% of the herds in this category have <40 sheep and/or goat heads. ^4^ 90% of the herds in this category have ≤10 cattle heads (all age groups). ^5^ 69% of the herds in this category have <100 pig heads. Categories related to type of production based on registered number of sows and finishers based on Schulz [23], i.e., finisher herd >7.5 finishers per sow; sow herd <5 finishers per sow; integrated herd 5–7.5 finishers per sow; FMD: foot-and-mouth disease; SPF: specific pathogen-free.

**Table 2:** Mitigation strategies investigated to control FMD outbreak in Denmark.

Abbreviation	Mitigation Strategies	Description
Basic	Basic control (reference)	- Three-day national standstill period for all livestock movements (model allows 2% illegal movements, i.e., 98% compliance) - 3 km PZ (with 98% compliance for direct contacts and 80% for indirect) around each infected herd - 10 km SZ (with 95% compliance for direct contacts and 70% for indirect) around each infected herd - Surveillance activities were modelled with the assumption of a false report ^1^ of infected herds by owners of 42% in PZs, 36% in SZs and 22% in FZs - Direct and indirect tracing of movements on and off infected herds within 14-day trace window; contact herds visited and/or tested (not applicable for contact herd (CH) mitigation strategy) - Culling, disposal, cleaning, and disinfection of confirmed infected herds
**All following mitigation strategies are on top of basic mitigation strategy**		
DP15	Depopulation triggered by 15 infected herds	Pre-emptive depopulation of susceptible herds within 1 km radius of infected herds, trigger after confirmation of 15 infected herds
DP15SZ15	Depopulation triggered by 15 infected herds plus enlarged SZ 15 km	Pre-emptive depopulation of all susceptible herds within 1 km radius of infected herds, trigger after confirmation of 15 infected herds, with enlargement of the SZs from 10 to 15 km
PZ5	Enlargement of PZ	Enlargement of PZ from 3 to 5 km
SZ15	Enlargement of SZ	Enlargement of SZ from 10 to 15 km
CH	Depopulation of dangerous contact herds	Pre-emptive culling of dangerous contact herds to infected herds based on tracing livestock and its products without consuming surveillance and laboratory resources to confirm the infection status
PV10_11_14d	Protective ring vaccination triggered 14 days after outbreak detection	Protective ring vaccination to increase probability of protection of susceptible animals from infection within 1 km radius outside SZs (i.e., 10–11 km radius from SZs), enforced 14 days after outbreak detection by keeping animals after vaccination (vaccination to live)
PV10_11_25 IH	Protective ring vaccination outside of SZ triggered by 25 infected herds	Protective ring vaccination within 1 km radius outside of SZs (i.e., 10–11 km from SZs), triggered once 25 infected herds were reached (i.e., if infected herds do not reach 25, then no vaccination) by keeping animals after vaccination (vaccination to live)
PV10_11_25 PC	Protective ring vaccination outside of SZ triggered by 25 pending culls	Protective ring vaccination within 1 km radius outside of SZs (i.e., 10–11 km radius from SZs), triggered once number of pending culls reached 25 herds per day (i.e., farms diagnosed with FMD but insufficient resources to start culling operations, if pending culls do not reach 25, then no vaccination), by keeping animals after vaccination (vaccination to live)
PV7_10_14d_ bov	Protective ring vaccination of cattle in outer 3 km of SZ triggered 14 days after outbreak detection	Protective ring vaccination of all cattle herds in the outer 3 km radius of the SZs (i.e., 7–10 km radius of SZs) enforced 14 days after outbreak detection
PV7_10_14d_ sui	Protective ring vaccination of pigs in outer 3 km of SZ triggered 14 days after outbreak detection	Protective ring vaccination of all pig herds in the outer 3 km radius of the SZs (i.e., 7–10 km radius of SZs) enforced 14 days after outbreak detection
PV7_10_14d_ ovi	Protective ring vaccination of small ruminants in outer 3 km of SZ triggered 14 days after outbreak detection	Protective ring vaccination of all ruminant herds in the outer 3 km radius of the SZs (i.e., 7–10 km radius of the SZs) enforced 14 days after outbreak detection
SV3km14d	Suppressive ring vaccination of all cattle, pigs, and small ruminants triggered 14 days after outbreak detection	Suppressive ring vaccination of all cattle, pigs, and small ruminants within a 3 km radius around each infected herd within PZs to suppress virus production and spread, enforced 14 days after outbreak detection by destroying the animals after vaccination when time and resources permit (vaccination to kill)
SV3km25IH	Suppressive ring vaccination of all cattle, pigs, and small ruminants triggered by 25 infected herds	Suppressive ring vaccination of all cattle, pigs, and small ruminants within a 3 km radius around each infected herd within PZs, triggered once 25 infected herds were reached (i.e., if infected herds do not reach 25, then no vaccination). Destroying vaccinated animals when time and resources permitted (vaccination to kill)
SV3km14d_ bov	Suppressive ring vaccination of cattle herds triggered 14 days after outbreak detection	Suppressive ring vaccination of cattle within a 3 km radius around each infected herd within PZs to suppress virus production and spread. Vaccination occurs around all infected holdings detected on or after day 14 of the control programme plus any farms diagnosed in the previous three days. All vaccinated animals culled when time and resources permitted (vaccination to kill)

^1^ False reports caused when, for example, livestock show clinical signs but are not actually infected with FMD. FMD: foot-and-mouth disease; SZ: surveillance zone; PZ: protection zone; FZ: free zone.

**Table 3:** A selected number of epidemiological and economic parameters used in the model.

Parameters	Value
Transmission rate ^1^ (ß)	0.5–2.2 (herd type-dependent)
Latent period ^1^ (days)	1–5 days (species-dependent)
Infectious period ^1^ (days)	5–10 days (species-dependent)
Incubation period ^1^ (days)	3–6 days (species-dependent)
Clinical period ^1^ (days)	10–14 days (species-dependent)
Probability of mortality ^1^	0.03–0.15 (herd type-dependent)
Number of days to report suspect premises after clinical signs	0–19 days (herd type-dependent)
Probability of reporting suspect cases	0.80–0.97 (herd type-dependent)
Ratio of false suspect premises reports to true reports ^2^	2.34:1
Time needed for direct trace (days)	0–3 days (species-dependent)
Time needed for indirect trace (days)	1–5 days (species-dependent)
Effectiveness of direct tracing	96–99% (species-dependent)
Effectiveness of indirect tracing	55–80% (species-dependent)
Effectiveness of vaccine ^1^	80–87% (species- and vaccine-dependent)
Immunity lag	6 days (i.e., from the time point an animal is vaccinated to the time point the animal achieves immunity)
Surveillance team ^3^	1 veterinarian and 1 technician for investigating clinical suspected herds, perform surveillance in zones and traced contact herds, including sampling of animals
Culling team ^3^	1 veterinarian, 4 technicians, and 1 truck driver
Disposal team ^3^	1 veterinarian, 4 technicians, and 1 truck driver
Cleaning and disinfection team^3^	1 veterinarian and 9 officers from the Danish Emergency Management Agency
Vaccination team^3^	1 veterinarian and 1 technician
Min/Max number of surveillance teams	8/65 (initial: pessimistic)| 16/130 (double in sensitivity analysis: optimistic)
Min/Max number of culling teams	3/37 (initial: pessimistic)| 6/74 (double in sensitivity analysis: optimistic)
Min/Max number of disposal teams	4/34 (initial: pessimistic)| 8/68 (double in sensitivity analysis: optimistic)
Min/Max number of decontamination teams	4/41 (initial: pessimistic)| 8/82 (double in sensitivity analysis: optimistic)
Min/Max number of vaccination teams	7/72 (initial: pessimistic)| 14/144 (double in sensitivity analysis: optimistic)
Days for herd surveillance visits ^4^	0.2–0.7 days (herd type-dependent)
Days to cull a herd ^4^	0.25–0.8 days (herd type-dependent)
Days to dispose of a herd ^4^	0.2–0.5 days (herd type-dependent)
Days to decontaminate premises ^4^	2–4 days (herd type-dependent)
Days to vaccinate a herd ^4^	0.2–0.7 days (herd type-dependent)
Surveillance visit costs ^5^ (per herd)	160–4227 EUR/herd plus staff time and laboratory tests (herd type-dependent)
Disinfection costs ^6^ (per herd)	81,081–283,784 EUR/herd plus staff time (herd type-dependent)
Culling costs (per animal)	1–36 EUR/animal (species-dependent)
Disposal costs (per animal)	18–118 EUR/animal (species-dependent)
Compensation costs (per animal) Vaccination costs (per animal) ^7^	154–1681 EUR/animal (species-dependent) 7.75–15.75 EUR/animal (species-dependent)
Disease control centre costs ^8^ (per centre)	10,607 EUR/day
Daily export value of live pig to non-EU countries ^9^	153,531 EUR/day
Daily export value of pig products to non-EU countries ^9^	7,142,620 EUR/day
Daily export value of live pig to EU countries ^9,10^	2,956,995 EUR/day
Daily export value of pig products to EU countries ^9,10^	2,925,764 EUR/day
Daily export value of live cattle to non-EU countries ^9^	71,121 EUR/day
Daily export value of live cattle to EU countries ^9,10^	111,698 EUR/day
Daily export value of beef to non-EU countries ^9^	66,165 EUR/day
Daily export value of beef to EU countries ^9,10^	867,370 EUR/day
Daily export value of dairy products to non-EU countries ^9,11^	3,934,152 EUR/day
Daily export value of dairy products to EU countries ^9–11^	3,383,179 EUR/day
ELISA costs ^12^	84–95 EUR/test
Daily ELISA capacity	3570/day
PCR costs	73 EUR/test
Daily PCR capacity	286/day
ELISA sensitivity ^13^	0.86–0.99
ELISA specificity ^13^	0.97–0.99
PCR sensitivity ^13^	0.95–0.99
PCR specificity ^13^	0.99–0.99
Clinical sensitivity of vaccinated animals	0.5–0.95 (species-dependent)
Clinical specificity of vaccinated animals	0.70
Clinical sensitivity of non-vaccinated animals	0.5–0.98 (species-dependent)
Clinical specificity of non-vaccinated animals	0.70
Average daily contribution margin per dairy cow ^14^	4.95/day
Average daily contribution margin per beef cattle ^14^	0.76/day
Average daily contribution margin per breeding pig ^14^	3.52/day
Average daily contribution margin per weaner pig ^14^	2.62/day
Average daily contribution margin per fattening pig ^14^	0.12/day
Average daily contribution margin per small ruminant ^14^	1.40/day

^1^Table S1 contains a detailed breakdown per species and herd type, including description of parametrisation of within-herd equation-based model. The values are based on Bradhurst et al. [3] and were derived from many different published FMD studies (see Supplementary Materials). ^2^ Value based on the study by McLaws et. al. [26]. ^3^ Composition of team defined based on performance of activities for average herd size of 90 cattle, 1600 pigs, and 17 small ruminants. Similar to Boklund et al. [27], we do not distinguish between the composition of teams in terms of staff qualifications, e.g., whether veterinarians are public employees or from private practice. Administrative staff only related to work in local crisis centre, and associated costs of component assigned to costs of control centres. ^4^ For example, 0.5 represents half a day and 1.25 one and a quarter days. ^5^ For example, clinical inspections of animals and decontamination of workers and equipment during surveillance visits and any consumables (e.g., blood tubes). ^6^ Covers decontamination/disinfection activities after culling, including equipment hire and consumables. ^7^ Including EUR 2.75 per vaccine/dose. ^8^ Including labour for 15 people per day per centre. ^9^ Values were calculated based on the annual statistics for Pigmeat, Beef and Dairy [28]. ^10^ EU27 (referred as EU countries). The UK withdrew from the EU28 in January 2020 and is now included as a third country (referred to as non-EU countries). ^11^ Dairy products: butter, cheese, preserved milk products, and liquid milk products. ^12^ ELISA costs differ between SPC and NSP ELISA. ^13^ Sensitivity and specificity of diagnostic tests varied between vaccinated and non-vaccinated animals. ^14^ Based on the calculations by SEGES [29]. N.B. If associated references are not mentioned in the footnotes, the parameters in this table are based on our own internal data collection/estimates.

## Data Availability

Not applicable.

## Supplementary Materials

The following supporting information can be downloaded at: https://www.mdpi.com/article/10.3390/pathogens12030435/s1, Figure S1: Geographical map of Denmark. Gray dots show all livestock farms (i.e., cattle, small ruminants, and pigs). Blue dots show all weather stations from which the data have been incorporated in the model. N.B. that the original location of the farms were scrambled within a 2.5 km radius circle. As Bornholm is more than 135 km away from the rest of Denmark, the island was not included in the model; Figure S2: Contact probability between different livestock herd types based on movement data in 2020; Table S1: Few parameterisation numbers of the within-herd equation-based model; Table S2: Post-outbreak surveillance sampling regime stratified by zone, number of sampled herds, and within-herd test procedure per species, based on the EU Directive (Garner et al., 2021; European Union, 2003); Table S3: Epidemiological outcomes of the simulation model, values shown as median (5th and 95th percentiles); Table S4: Economic outcomes of the simulation model, values shown as median (5th and 95th percentiles). References [53,54,55,56,57,58,59,60] are cited in the Supplementary Materials.
