# Supplementary material for: Simulation of Foot-and-Mouth Disease Spread and Effects of Mitigation Strategies to Support Veterinary Contingency Planning in Denmark
Source: Pathogens. 2023 Mar 9;12(3):435. doi: 10.3390/pathogens12030435 (PMC10056164; doi:10.3390/pathogens12030435)
Supplement: Supplementary file 1 [file pathogens-12-00435-s001.zip › pathogens-2259365-supplementary - updated.pdf]

Supplementary Material

Figure S1: Geographical map of Denmark. Gray dots show all livestock farms (i.e. cattle, small ruminant and pigs). Blue dots show all weather stations from which the data has been incorporated in the model. N.B. that the orginal location of the farms were scrambled within a 2.5 km radius circle. As Bornholm is more than 135 km away from rest of Denmark, the Island was not included in the model.

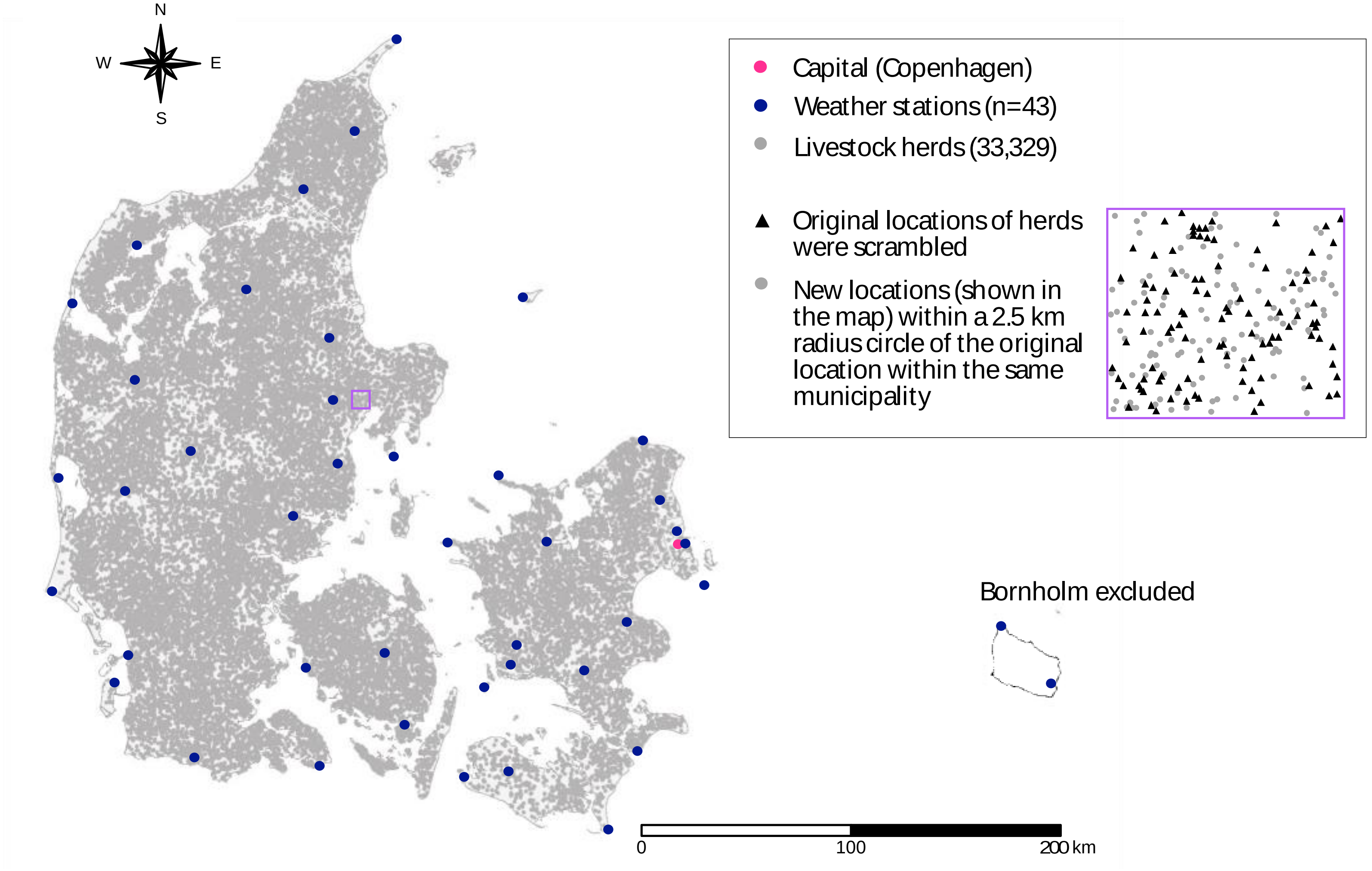

Figure S2: Contact probability between different livestock herd types based on movement data in 2020.

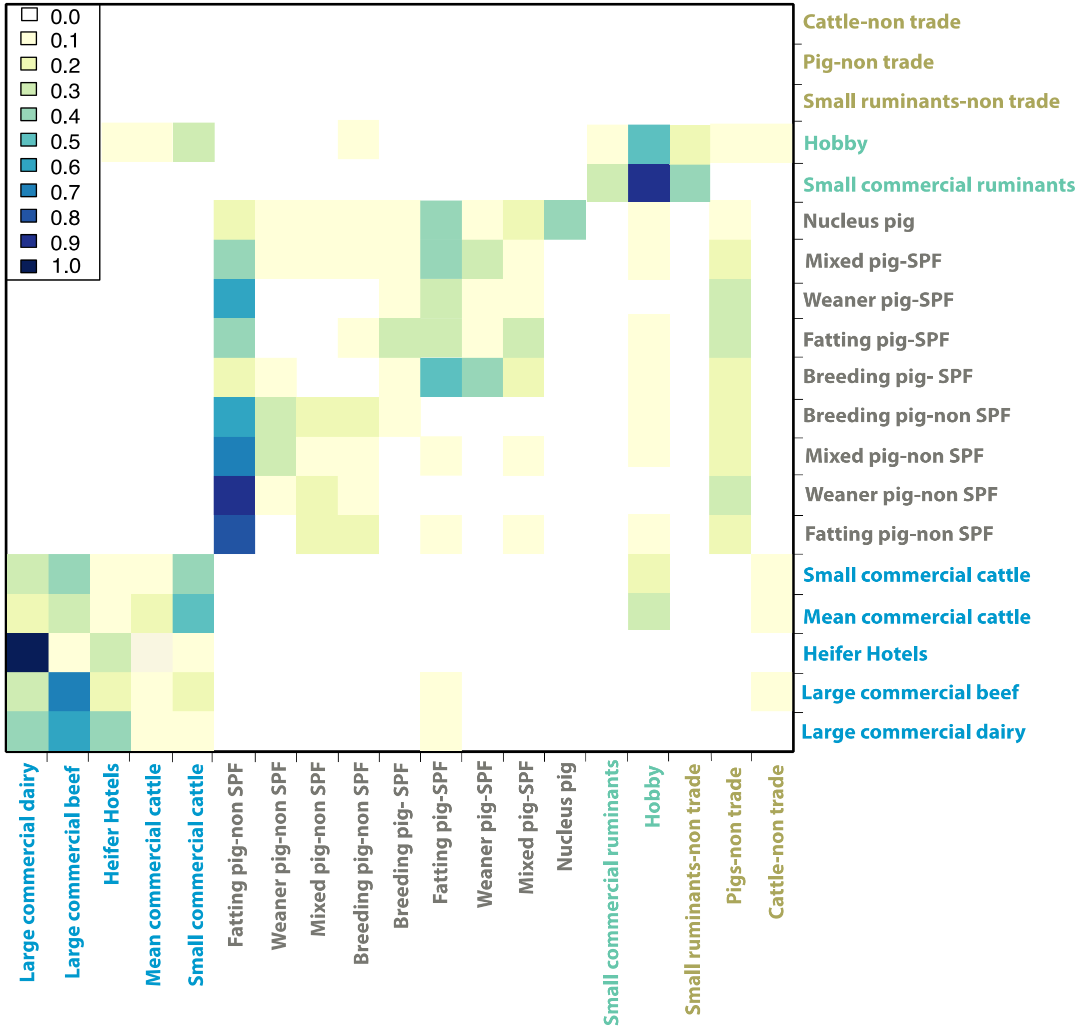

Table S1: Few parameterization numbers of the within-herd equation-based model.

Herd Types	Transmission rate (ß)[1]	Latent period (days)[2]	Infectious period (days)[3]	Incubation period (days)[4]	Clinical period (days)[5]	Probability of mortality[6]
Large commercial dairy	2.1	2	10	3	12	0.05
Large commercial beef	1.6	2	10	3	12	0.05
Heifer Hotel	1.8	2	10	3	12	0.05
Mean commercial cattle	1.8	2	10	3	12	0.05
Small commercial cattle	1.8	2	10	3	12	0.05
Small commercial ruminants	0.5	5	10	6	10	0.03
Large-scale commercial fattening pig, non-SPF	2.2	1	6	5	14	0.03
Large-scale commercial weaner pig herd, non-SPF	2.2	1	6	5	14	0.03
Large-scale commercial mixed pig herd, non-SPF	2.2	1	6	5	14	0.03
Large scale commercial breeding pig, non-SPF	2.2	1	6	5	14	0.15
Large scale commercial breeding pig, SPF	2.2	1	6	5	14	0.15
Hobby farms	1.5	4	5	4	12	0.03
Large-scale commercial fattening pig herd, SPF	2.2	1	6	5	14	0.03
Large-scale commercial weaner pig herd, SPF	2.2	1	6	5	14	0.03
Large-scale commercial mixed pig herd, SPF	2.2	1	6	5	14	0.03
Nucleus pig herd	2.2	1	6	5	14	0.03
Small ruminant without outgoings movements	0.5	5	10	6	10	0.03
Cattle farms without outgoings movements	1.8	2	10	3	12	0.05
Pig farms without outgoings movements	2.2	1	6	5	14	0.03

[1] Transmission rate = contact rate x transmission probability

[2] The period from initial infection to when an animal begins excreting FMD virus

[3] The period during which infected animals excrete virus and are thus capable of spreading the infection

[4] The period between exposure and onset of clinical symptoms

[5] Days of showing clinical signs. N.B. FMD not always be apparent clinically, especially in sheep

[6] Mortality rates are usually low, except in very young animals

Table S2. Post-outbreak surveillance sampling regime stratified by zone, number of sampled herds, and within-herd test procedure per species, based on EU Directive (based on the study by Garner et al., 2021; European Union, 2003).

**Protection zone**	**Sampling intensity (herds)**	**Test procedure within herds**
**Cattle**	Inspect all[1]	Clinical inspection only
**Sheep**	Inspect all	Clinical + 95:5 animal sampling (serology)[3]
**Pigs**	Inspect all	Clinical inspection only
**Surveillance zone**		
**Cattle**	95:2 random sample[2]	Clinical inspection only
**Sheep**	95:2 random sample	Clinical + 95:5 sampling (serology)
**Pigs**	95:2 random sample	Clinical inspection only
**Vaccination zone**[4]		
**Cattle**	Inspect all	Clinical + all animals (serology)
**Sheep**	Inspect all	Clinical + all animals (serology)
**Pigs**	Inspect all	Clinical + 95:5 animal sampling (serology)[5]

[1] Clinical inspection of all susceptible animals in all herds. Post-outbreak surveillance in previously infected areas is assumed to begin 30 days after last infected holding is found.
[2] Randomly select sufficient herds to be 95% confident of detecting infection assuming a 2% herd prevalence (95:2). The exact number of holdings that need to be sampled depends on total number of eligible holdings.
[3] Within-herd sampling regime of 95:5 involves testing sufficient animals to achieve 95% confidence that an infected prevalence of at least 5% would be detected (Bradhurst et al., 2021).
[4] Note that surveillance of vaccinated population is only required under a vaccinate-and-retain policy and the test procedure shown is valid if all animals are vaccinated.
[5] Sampling all pigs is considered impractical. NB. we assume that any sample that tests positive within the post-outbreak surveillance would be subject to a confirmatory test as uncertainty about the presence of infection would be of concern for national disease managers, particularly the number of infected herds that are not detected (false negative) and the number of non-infected herds that are incorrectly tested as infected (false positive). Performance of laboratory tests is measured in terms of sensitivity and specificity. Any sample that tests positive in an initial (screening) test would be subject to a second confirmatory test. For unvaccinated populations this could involve two different structural protein serological tests or a structural and non-structural protein test. For vaccinated populations a combination of non-structural tests may be used, as vaccinated animals will test positive using structural protein serological tests (Garner et al., 2021).

Table S3: Epidemiological outcomes of the simulation model. Values shown as Median (5th and 95th percentiles)

Mitigation measures stratified by epidemic initiation scenarios	Number of infected farms	Control duration (in days)	Depopulated farms	Vaccinated farms	Post-Outbreak (end day)	Clinical inspected herds	Number of tested herds (non-vaccinated)/(vaccinated)	Total animals culled
**North_Cattle (SZ 1)**								
**Basic**	2 (0-289)	35 (0-104)	2 (0-288)	0 (0-0)	64 (1-216)	339 (1-10777)	1240 (1-32212)	108 (0-108779)
**DP15**	2 (0-257)	35 (0-114)	2 (0-795)	0 (0-0)	64 (1-242)	357 (1-15822)	1245 (1-46265)	116 (0-409811)
**DP15SZ15**	2 (0-294)	35 (0-125)	2 (0-918)	0 (0-0)	64 (1-209)	383 (1-11181)	1315 (1-33281)	250 (0-491166)
**PZ5**	3 (0-332)	36 (0-112)	3 (0-328)	0 (0-0)	65 (1-270)	481 (1-16977)	1486 (1-49653)	392 (0-125265)
**SZ15**	2 (0-258)	36 (0-102)	2 (0-257)	0 (0-0)	64 (1-206)	347 (1-10972)	1252 (1-32644)	243 (0-99352)
**CH**	3 (0-268)	36 (0-97)	3 (0-350)	0 (0-0)	62 (1-209)	526 (1-11729)	1692 (1-34862)	410 (0-127879)
**PV1km14d**	2 (0-279)	35 (0-105)	2 (0-279)	0 (0-2637)	64 (1-253)	355 (1-12459)	1259 (1-30349)/ 0 (0-98831)	178 (0-103929)
**PV1km25IH**	3 (0-301)	36 (0-109)	3 (0-298)	0 (0-2641)	65 (1-238)	415 (1-11852)	1348 (1-31058)/ 0 (0-80636)	275 (0-111091)
**PV1km25PC**	2 (0-342)	36 (0-117)	2 (0-342)	0 (0-0)	64 (1-229)	390 (1-11086)	1291 (1-33183)	250 (0-132409)
**PV3km14d_bov**	2 (0-250)	36 (0-101)	2 (0-250)	0 (0-4574)	65 (1-302)	399 (1-13240)	1210 (1-24006)/ 0 (0-179977)	217 (0-99230)
**PV3km14d_sui**	2 (0-293)	35 (0-106)	2 (0-291)	0 (0-1038)	65 (1-232)	374 (1-11322)	1284 (1-31106)/0 (0-76985)	235 (0-116046)
**PV3km14d_ovi**	3 (0-263)	36 (0-101)	3 (0-263)	0 (0-2558)	65 (1-247)	424 (1-12273)	1323 (0-31068)/0 (0-41959)	273 (0-97935)

**SV3km14d**	2 (0-288)	36 (0-106)	2 (0-286)	0 (0-3252)	64 (1-1220)	367 (1-8767)	1255 (1-25909)	169 (0-112494) / 0 (0-2007420)
**SV3km25IH**	2 (0-282)	35 (0-110)	2 (0-280)	0 (0-2903)	64 (1-1224)	366 (1-8201)	1231 (1-23691)	145 (0-106421) / 0 (0-2007231)
**SV3km14d_bov**	2 (0-250)	36 (0-98)	2 (0-250)	0 (0-1332)	62 (1-344)	407 (1-9448)	1382 (1-24721)	219 (0-91013) / 0 (0-259929)
**Central_Cattle (SZ 1)**								
**Basic**	5 (0-335)	39 (0-119)	5 (0-333)	0 (0-0)	72 (1-231)	964 (1-11451)	2714 (1-34088)	1522 (0-125808)
**DP15**	4 (0-328)	39 (0-134)	4 (0-1022)	0 (0-0)	69 (1-216)	748 (1-11402)	2074 (1-34297)	1225 (0-539976)
**DP15SZ15**	5 (0-345)	40 (0-140)	5 (0-1070)	0 (0-0)	72 (1-220)	963 (1-11820)	2839 (1-35173)	1462 (0-555116)
**PZ5**	5 (0-407)	39 (0-124)	5 (0-406)	0 (0-0)	72 (1-298)	857 (1-18303)	2315 (1-52396)	1414 (0-148119)
**SZ15**	7 (0-357)	41 (0-117)	7 (0-354)	0 (0-0)	76 (1-230)	1138 (1-11757)	3533 (1-34772)	1996 (0-139484)
**CH**	4 (0-356)	39 (0-116)	5 (0-476)	0 (0-0)	69 (1-244)	1036 (1-12819)	2657 (1-39105)	1597 (0-175800)
**PV1km14d**	5 (0-390)	40 (0-123)	5 (0-389)	0 (0-2887)	74 (1-273)	1061 (1-13056)	3003 (1-32469) / 0 (0-101987)	1701 (0-144701)
**PV1km25IH**	5 (0-386)	39 (0-123)	5 (0-384)	0 (0-2796)	72 (1-254)	900 (1-12423)	2612 (1-33580) / 0 (0-80690)	1991 (0-148202)
**PV1km25PC**	4 (0-373)	39 (0-123)	4 (0-372)	0 (0-0)	70 (1-236)	762 (1-11586)	2168 (1-33984)	1187 (0-136713)
**PV3km14d_bov**	6 (0-356)	40 (0-118)	6 (0-355)	0 (0-5707)	74 (1-354)	964 (1-14370)	1873 (1-24879) / 0 (0-222817)	1459 (0-135196)
**PV3km14d_sui**	5 (0-402)	39 (0-124)	5 (0-399)	0 (0-1053)	70 (1-259)	795 (1-12493)	2370 (1-34623) / 0 (0-79963)	1238 (0-149368)
**PV3km14d_ovi**	5 (0-402)	39 (0-127)	5 (0-400)	0 (0-3567)	71 (1-301)	801 (1-13990)	2059 (1-33777) / 0 (0-57943)	1319 (0-156362)
**SV3km14d**	6 (0-379)	40 (0-128)	6 (0-379)	0 (0-3439)	74 (1-1246)	950 (1-9394)	3042 (1-27269)	1687 (0-145767) / 0 (0-2009984)

**SV3km25IH**	5 (0-423)	40 (0-130)	5 (0-421)	0 (0-3286)	71 (1-1241)	769 (1-9266)	2410 (1-27656)	1663 (0-164735) / 0 (0-2009185)
**SV3km14d_bov**	4 (0-357)	39 (0-117)	4 (0-357)	0 (0-1918)	67 (1-457)	741 (1-9876)	2870 (1-25305)	1312 (0-128121) / 0 (0-380189)
**South_Cattle (SZ 1)**								
**Basic**	12 (0-528)	45 (0-148)	12 (0-526)	0 (0-0)	86 (1-277)	2134 (1-13085)	6021 (1-39074)	4450 (0-207986)
**DP15**	12 (0-453)	45 (0-169)	12 (0-1414)	0 (0-0)	88 (1-248)	2245 (1-12900)	6476 (1-39154)	4466 (0-712246)
**DP15SZ15**	12 (0-471)	45 (0-173)	12 (0-1463)	0 (0-0)	86 (1-248)	2130 (1-12861)	6238 (1-37916)	4409 (0-708263)
**PZ5**	15 (0-484)	46 (0-138)	14 (0-482)	0 (0-0)	95 (1-315)	2919 (1-19212)	8177 (1-55634)	5322 (0-192045)
**SZ15**	14 (0-491)	45 (0-139)	14 (0-491)	0 (0-0)	90 (1-266)	2613 (1-12807)	7315 (1-38284)	5173 (0-188077)
**CH**	13 (0-489)	45 (0-139)	17 (0-631)	0 (0-0)	93 (1-276)	3374 (1-14143)	9377 (1-41490)	5846 (0-235647)
**PV1km14d**	13 (0-546)	45 (0-150)	12 (0-544)	56 (0-3096)	89 (1-311)	2216 (1-13580)	6017 (1-34390) / 350 (0-104195)	4251 (0-203343)
**PV1km25IH**	14 (0-471)	46 (0-138)	14 (0-470)	0 (0-2841)	93 (1-279)	2428 (1-13049)	7196 (1-35466) / 0 (0-85489)	5611 (0-177554)
**PV1km25PC**	14 (0-553)	45 (0-154)	14 (0-552)	0 (0-0)	93 (1-288)	2700 (1-13209)	7451 (1-39135)	5557 (0-212977)
**PV3km14d_bov**	13 (0-423)	45 (0-131)	13 (0-420)	72 (0-6197)	92 (1-365)	2467 (1-14804)	4191 (1-24455) / 2901 (0-228879)	4779 (0-162685)
**PV3km14d_sui**	12 (0-436)	44 (0-135)	12 (0-436)	12 (0-1067)	88 (1-276)	2137 (1-12699)	6232 (1-35221) / 1 (0-81024)	3962 (0-164186)
**PV3km14d_ovi**	17 (0-474)	46 (0-141)	17 (0-470)	44 (0-3930)	98 (1-328)	3258 (1-14864)	7488 (1-35936) / 815 (0-63428)	5635 (0-178483)
**SV3km14d**	16 (0-501)	46 (0-145)	16 (0-499)	43 (0-3435)	98 (1-1252)	2954 (1-9834)	8307 (1-28149)	5064 (0-189499) / 23736 (0-2010446)
**SV3km25IH**	14 (0-469)	45 (0-139)	14 (0-468)	0 (0-3498)	92 (1-1252)	2554 (1-9615)	7606 (1-28117)	4908 (0-168753) / 0 (0-2010802)

**SV3km14d_bov**	16 (0-451)	46 (0-135)	16 (0-450)	16 (0-2414)	91 (1-553)	2878 (1-10399)	5909 (1-25932)	5324 (0-165951) / 2885 (0-468393)
**Zealand_Cattle (SZ 1)**								
**Basic**	1 (0-78)	30 (0-65)	1 (0-78)	0 (0-0)	58 (1-167)	205 (1-9633)	455 (1-27825)	18 (0-28070)
**DP15**	1 (0-67)	30 (0-67)	1 (0-190)	0 (0-0)	58 (1-160)	203 (1-9418)	461 (1-25917)	17 (0-90106)
**DP15SZ15**	1 (0-78)	30 (0-71)	1 (0-223)	0 (0-0)	58 (1-174)	192 (1-10020)	415 (1-28331)	15 (0-93746)
**PZ5**	1 (0-88)	30 (0-70)	1 (0-87)	0 (0-0)	58 (1-184)	197 (1-11186)	417 (1-31744)	15 (0-30518)
**SZ15**	1 (0-66)	30 (0-65)	1 (0-66)	0 (0-0)	58 (1-160)	197 (1-9374)	431 (1-25835)	16 (0-23444)
**CH**	1 (0-82)	30 (0-68)	1 (0-114)	0 (0-0)	56 (1-173)	198 (1-10786)	441 (1-31836)	16 (0-35894)
**PV1km14d**	1 (0-60)	30 (0-60)	1 (0-60)	0 (0-562)	58 (1-153)	200 (1-8439)	428 (1-22756) / 0 (0-7680)	16 (0-20511)
**PV1km25IH**	1 (0-70)	30 (0-67)	1 (0-69)	0 (0-1923)	58 (1-181)	197 (1-9489)	446 (1-24578) / 0 (0-38880)	15 (0-22989)
**PV1km25PC**	1 (0-76)	30 (0-68)	1 (0-76)	0 (0-0)	58 (1-170)	199 (1-9493)	422 (1-27101)	17 (0-26775)
**PV3km14d_bov**	1 (0-65)	30 (0-64)	1 (0-64)	0 (0-716)	58 (1-178)	191 (1-8954)	416 (1-20378) / 0 (0-28778)	15 (0-22428)
**PV3km14d_sui**	1 (0-72)	30 (0-65)	1 (0-71)	0 (0-196)	58 (1-160)	200 (1-9006)	420 (1-25085) / 0 (0-6913)	17 (0-22716)
**PV3km14d_ovi**	1 (0-73)	30 (0-65)	1 (0-73)	0 (0-475)	58 (1-168)	194 (1-9417)	419 (1-24849) / 0 (0-8169)	15 (0-24668)
**SV3km14d**	1 (0-59)	30 (0-64)	1 (0-59	0 (0-386)	58 (1-201)	201 (1-7955)	437 (1-22716)	15 (0-21451) / 0 (0-203384)
**SV3km25IH**	1 (0-70)	30 (0-65)	1 (0-70)	0 (0-1205)	58 (1-429)	196 (1-7372)	435 (1-21009)	16 (0-27141) / 0 (0-626903)
**SV3km14d_bov**	1 (0-58)	30 (0-60)	1 (0-58)	0 (0-134)	56 (1-149)	205 (1-8441)	464 (1-21140)	17 (0-20013) / 0 (0-25644)

**Entire_DK_Diary (SZ 2)**								
**Basic**	136 (19-735)	79 (46-179)	135 (19-732)	0 (0-0)	180 (101-322)	9502 (3420-14680)	27955 (9047-44437)	52197 (5882-277535)
**DP15**	128 (19-693)	81 (49-232)	398 (31-2121)	0 (0-0)	173 (95-300)	9386 (3689-15335)	27887 (10809-45090)	194943 (11217-1053971)
**DP15SZ15**	133 (20-649)	81 (50-220)	412 (35-2017)	0 (0-0)	174 (98-286)	9594 (3737-14771)	28373 (10943-43304)	199090 (14259-1011028)
**PZ5**	130 (20-670)	77 (46-166)	129 (20-666)	0 (0-0)	199 (101-365)	12305 (3655-20951)	35443 (8700-61992)	47715 (6233-251715)
**SZ15**	136 (18-663)	77 (47-163)	136 (18-662)	0 (0-0)	179 (98-298)	9814 (3484-14195)	28906 (9628-42782)	50049 (6425-248784)
**CH**	134 (20-716)	78 (46-172)	189 (26-895)	0 (0-0)	178 (106-329)	10255 (4690-16125)	30055 (12304-48879)	67802 (9015-329897)
**PV1km14d**	134 (23-670)	77 (48-166)	134 (23-666)	1659 (123-3441)	236 (108-365)	13425 (4192-20901)	32529 (10377-56912) / 66817 (782-109562)	50336 (7589-246317)
**PV1km25IH**	140 (21-712)	79 (46-173)	139 (21-708)	1280 (0-3274)	233 (105-365)	13216 (3876-21045)	33151 (10341-59941) / 68294 (0-88415)	51832 (6532-258430)
**PV1km25PC**	136 (21-793)	77 (47-190)	135 (21-790)	0 (0-0)	179 (104-335)	9508 (3660-14356)	27671 (9116-43147)	50304 (7527-294309)
**PV3km14d**	136 (20-641)	77 (47-166)	136 (20-639)	1073 (39-4878)	201 (101-365)	10181 (3449-15443)	25289 (4703-31009) / 32022 (192-175922)	48729 (6229-248571)
**PV3km14d_sui**	138 (20-635)	78 (47-167)	138 (20-632)	708 (28-1101)	189 (104-321)	9963 (3576-14564)	27345 (10373-40489) / 40312 (420-83974)	53431 (6278-238393)
**PV3km14d_ovi**	131 (22-769)	77 (46-189)	131 (21-768)	552 (20-3243)	187 (102-365)	10016 (3825-15356)	27747 (8476-40644) / 6392 (37-47417)	49162 (6364-290516)
**SV3km14d**	136 (23-653)	79 (47-173)	136 (23-651)	1384 (68-3623)	463 (107-1268)	10845 (4179-17429)	31145 (11185-51952)	52090 (8142-249623) / 769536 (33085-2012313)
**SV3km25IH**	133 (20-664)	78 (45-168)	133 (20-660)	2149 (0-3614)	721 (101-1251)	9801 (3685-18747)	28149 (8749-55640)	49156 (6325-252339) / 1288102 (0-2011771)
**SV3km14d_bov**	137 (21-706)	77 (48-178)	136 (21-703)	600 (26-3471)	209 (102-728)	9771 (3650-14170)	20954 (7421-28882)	49514 (7051-265102) / 122328 (4510-664626)
**North_Pig (SZ 1)**								

**Basic**	19 (0-356)	49 (0-117)	19 (0-350)	0 (0-0)	93 (1-226)	2632 (1-11266)	8359 (1-33018)	8073 (0-147673)
**DP15**	22 (0-329)	51 (0-131)	41 (0-1013)	0 (0-0)	97 (1-212)	2867 (1-10839)	9038 (1-32523)	18296 (0-525966)
**DP15SZ15**	21 (0-312)	51 (0-124)	38 (0-919)	0 (0-0)	95 (1-207)	2820 (1- 10708)	8866 (1-32041)	19725 (0-494001)
**PZ5**	20 (0-335)	49 (0-111)	19 (0-330)	0 (0-0)	96 (1-256)	2730 (1-15157)	8431 (1-44987)	8260 (0-129330)
**SZ15**	20 (0-359)	49 (0-118)	19 (0-355)	0 (0-0)	95 (1-220)	2793 (1-11241)	8690 (1-33130)	8883 (0-153012)
**CH**	20 (0-337)	49 (0-114)	26 (0-434)	0 (0-0)	100 (1-226)	3830 (1-11911)	11680 (1-35726)	11285 (0-174532)
**PV1km14d**	20 (0-345)	48 (0-118)	20 (0-341)	109 (0-2865)	95 (1-268)	2605 (1-12520)	8044 (1-30230) / 1408 (0-99354)	7799 (0-139025)
**PV1km25IH**	22 (0-353)	50 (0-117)	22 (0-349)	0 (0-2889)	100 (1-245)	2995 (1-11910)	9337 (1-30594) /0 (0-83052)	8912 (0-139469)
**PV1km25PC**	19 (0-377)	49 (0-124)	19 (0-375)	0 (0-0)	94 (1-232)	2677 (1-11123)	8217 (1-33206)	8393 (0-160572)
**PV3km14d_bov**	22 (0-331)	50 (0-112)	21 (0-325)	141 (0-5095)	101 (1-333)	2927 (1-13305)	7543 (1-22911) / 5606 (0-192821)	8915 (0-135357)
**PV3km14d_sui**	20 (0-307)	48 (0-108)	19 (0-304)	30 (0-1037)	96 (1-230)	2875 (1-11037)	8950 (1-30675) / 786 (0-76718)	8543 (0-128404)
**PV3km14d_ovi**	20 (0-412)	48 (0-127)	19 (0-407)	49 (0-3304)	96 (1-295)	2709 (1-13245)	8277 (1-31677) / 842 (0-51409)	8305 (0-162518)
**SV3km14d**	20 (0-409)	49 (0-134)	19 (0-403)	59 (0-3268)	102 (1-1246)	2662 (1-9056)	8463 (1-26979)	7850 (0-157677) / 31442 (0-2008746)
**SV3km25IH**	21 (0-366)	50 (0-125)	21 (0-364)	0 (0-3158)	100 (1-1233)	2771 (1-8475)	8553 (1-24159)	9235 (0-145513) / 0 (0-2008844)
**SV3km14d_bov**	21 (0-337)	49 (0-116)	20 (0-336)	24 (0-1632)	92 (1-407)	2644 (1-10785)	7817 (1-24015)	8517 (0-132010) / 4737 (0-327618)
**Central_Pig (SZ 1)**								
**Basic**	25 (0-430)	51 (0-133)	25 (0-427)	0 (0-0)	103 (1-245)	3530 (1-11445)	10439 (1-34413)	10785 (0-173829)

**DP15**	27 (0-384)	53 (0-149)	55 (0-1166)	0 (0-0)	106 (1-230)	3888 (1-11518)	11634 (1-33979)	29883 (0-600881)
**DP15SZ15**	24 (0-375)	52 (0-146)	49 (0-1159)	0 (0-0)	102 (1-221)	3604 (1-11501)	10717 (1-34572)	25119 (0-595399)
**PZ5**	25 (0-388)	51 (0-120)	24 (0-385)	0 (0-0)	104 (1-279)	3675 (1-17476)	10878 (1-50136)	11436 (0-160248)
**SZ15**	23 (0-379)	50 (0-118)	23 (0-375)	0 (0-0)	101 (1-231)	3261 (1-11376)	9557 (1-33826)	10248 (0-150099)
**CH**	23 (0-312)	50 (0-111)	32 (0-424)	0 (0-0)	110 (1-227)	4862 (1-11932)	14487 (1-35718)	14221 (0-167099)
**PV1km14d**	27 (0-467)	51 (0-139)	27 (0-463)	190 (0-2915)	109 (1-287)	3781 (1-13077)	10910 (1-33244) / 2488 (0-100911)	11372 (0-186378)
**PV1km25IH**	28 (0-341)	51 (0-118)	27 (0-336)	209 (0-2882)	108 (1-247)	3856 (1-11794)	11235 (1-31547) / 2619 (0-82332)	11352 (0-137046)
**PV1km25PC**	24 (0-383)	50 (0-121)	23 (0-381)	0 (0-0)	101 (1-234)	3344 (1-11168)	9910 (1-33261)	10808 (0-151069)
**PV3km14d_bov**	23 (0-380)	51 (0-121)	23 (0-379)	162 (0-5666)	105 (1-356)	3395 (1-14223)	8227 (1-23150) / 6574 (0-214813)	10518 (0-152495)
**PV3km14d_sui**	25 (0-386)	51 (0-127)	24 (0-380)	48 (0-1050)	106 (1-253)	3681 (1-11708)	10651 (1-31972) / 1271 (0-79178)	10635 (0-159152)
**PV3km14d_ovi**	23 (0-423)	49 (0-131)	22 (0-418)	69 (0-3507)	102 (1-300)	3317 (1-13916)	9938 (1-33701) / 1274 (0-57431)	9943 (0-169126)
**SV3km14d**	24 (0-388)	50 (0-129)	23 (0-387)	71 (0-3200)	108 (1-1242)	3210 (1-9096)	9527 (1-26529)	10204 (0-154408) / 43328 (0-2009158)
**SV3km25IH**	25 (0-357)	51 (0-125)	24 (0-352)	0 (0-3126)	108 (1-1235)	3384 (1-8440)	10076 (1-25021)	10681 (0-152327) / 0 (0-2008526)
**SV3km14d_bov**	22 (0-317)	49 (0-112)	22 (0-315)	27 (0-1668)	98 (1-419)	3113 (1-10914)	8746 (1-24139)	9652 (0-131827) / 5544 (0-339562)
**South_Pig (SZ 1)**								
**Basic**	25 (0-639)	50 (0-166)	25 (0-638)	0 (0-0)	103 (1-294)	3553 (1-12960)	9807 (1-38722)	9868 (0-249125)
**DP15**	27 (0-657)	54 (0-215)	60 (0-1953)	0 (0-0)	103 (1-268)	3885 (1-14136)	11053 (1-40634)	28163 (0-976200)

**DP15SZ15**	27 (0-642)	54 (0-213)	58 (0-1888)	0 (0-0)	105 (1-262)	4110 (1-13898)	11614 (1-40918)	27374 (0-959598)
**PZ5**	25 (0-647)	52 (0-164)	24 (0-645)	0 (0-0)	106 (1-358)	3613 (1-18992)	9448 (1-55239)	9932 (0-257784)
**SZ15**	24 (0-607)	52 (0-159)	24 (0-603)	0 (0-0)	103 (1-283)	3411 (1-12806)	9305 (1-37308)	10603 (0-241488)
**CH**	23 (0-549)	50 (0-145)	31 (0-702)	0 (0-0)	107 (1-289)	4744 (1-13592)	12555 (1-39558)	12122 (0-270584)
**PV1km14d**	27 (0-652)	52 (0-169)	26 (0-649)	187 (0-3038)	107 (1-328)	3506 (1-13448)	9269 (1-34534) / 2689 (0-102848)	10689 (0-254845)
**PV1km25IH**	25 (0-639)	51 (0-170)	25 (0-637)	48 (0-3088)	106 (1-12996)	3572 (1-12996)	9298 (1-34415) / 0 (0-89999)	9930 (0-247548)
**PV1km25PC**	24 (0-676)	51 (0-174)	23 (0-670)	0 (0-0)	101 (1-301)	3113 (1-12611)	8459 (1-38062)	9775 (0-269470)
**PV3km14d_bov**	27 (0-513)	51 (0-146)	26 (0-505)	200 (0-6467)	110 (1-365)	3854 (1-14546)	7211 (1-23416) / 8168 (0-225836)	10873 (0-209534)
**PV3km14d_sui**	25 (0-600)	51 (0-162)	24 (0-593)	43 (0-1069)	104 (1-312)	3586 (1-13368)	9404 (1-36986) / 1182 (0-82305)	10058 (0-243414)
**PV3km14d_ovi**	24 (0-679)	51 (0-174)	24 (0-676)	81 (0-4422)	105 (1-365)	3405 (1-14209)	8122 (1-33771) / 1371 (0-63285)	9976 (0-270614)
**SV3km14d**	25 (0-579)	52 (0-166)	25 (0-576)	88 (0-3408)	111 (1-1263)	3560 (1-9558)	9441 (1-27667)	10307 (0-243034) / 47649 (0-2010559)
**SV3km25IH**	29 (0-653)	51 (0-168)	28 (0-649)	115 (0-3411)	117 (1-1258)	3823 (1-10117)	10439 (1-29726)	11160 (0-249313) / 52512 (0-2010686)
**SV3km14d_bov**	25 (0-573)	51 (0-162)	24 (0-571)	36 (0-2757)	100 (1-607)	3352 (1-12188)	8048 (1-25130)	9906 (0-230743) / 6886 (0-543897)
**Zealand_Pig (SZ 1)**								
**Basic**	2 (0-56)	32 (0-60)	1 (0-55)	0 (0-0)	59 (1-128)	230 (1-5775)	481 (1-15801)	396 (0-28917)
**DP15**	2 (0-54)	34 (0-63)	2 (0-151)	0 (0-0)	61 (1-130)	230 (1-6238)	501 (1-16968)	432 (0-78246)
**DP15SZ15**	2 (0-51)	34 (0-61)	2 (0-141)	0 (0-0)	60 (1-126)	233 (1-5801)	518 (1-15316)	437 (0-70021)

**PZ5**	2 (0-54)	34 (0-60)	2 (0-52)	0 (0-0)	61 (1-127)	235 (1-5746)	488 (1-14828)	473 (0-25819)
**SZ15**	2 (0-56)	34 (0-60)	1 (0-54)	0 (0-0)	59 (1-130)	229 (1-5857)	483 (1-15519)	383 (0-30934)
**CH**	2 (0-51)	34 (0-59)	2 (0-71)	0 (0-0)	57 (1-138)	236 (1-7696)	516 (1-21502)	525 (0-32431)
**PV1km14d**	2 (0-56)	34 (0-62)	2 (0-55)	0 (0-551)	61 (1-135)	233 (1-6286)	499 (1-16054) / 0 (0-7632)	441 (0-28296)
**PV1km25IH**	2 (0-48)	34 (0-59)	1 (0-47)	0 (0-1171)	59 (1-130)	225 (1-5475)	468 (1-12704) / 0 (0-18534)	438 (0-27942)
**PV1km25PC**	2 (0-56)	34 (0-60)	1 (0-54)	0 (0-0)	59 (1-127)	229 (1-5707)	468 (1-15028)	390 (0-30617)
**PV3km14d_bov**	2 (0-59)	34 (0-62)	1 (0-57)	0 (0-441)	59 (1-137)	230 (1-5856)	488 (1-13320) / 0 (0-16675)	413 (0-29281)
**PV3km14d_sui**	2 (0-54)	34 (0-60)	2 (0-53)	0 (0-120)	60 (1-127)	234 (1-5611)	499 (1-14820) / 0 (0-4350)	473 (0-28638)
**PV3km14d_ovi**	2 (0-59)	32 (0-61)	1 (0-57)	0 (0-302)	59 (1-135)	224 (1-6403)	482 (1-16243) / 0 (0-4352)	329 (0-26726)
**SV3km14d**	1 (0-54)	31 (0-61)	1 (0-54)	0 (0-243)	59 (1-144)	227 (1-5558)	467 (1-15722)	362 (0-25931) / 0 (0-111740)
**SV3km25IH**	1 (0-49)	31 (0-60)	1 (0-49)	0 (0-638)	59 (1-269)	229 (1-4816)	486 (1-13311)	389 (0-28435) / 0 (0-352349)
**SV3km14d_bov**	2 (0-54)	32 (0-60)	1 (0-53)	0 (0-93)	56 (1-125)	227 (1-5718)	473 (1-14749)	438 (0-27084) / 0 (0-16755)
**Entire_DK_Weaner (SZ 2)**								
**Basic**	61 (2-616)	63 (35-161)	59 (2-605)	0 (0-0)	143 (62-292)	6909 (290-12832)	20432 (550-38215)	24572 (1556-229912)
**DP15**	59 (2-524)	64 (35-184)	163 (2-1598)	0 (0-0)	138 (59-262)	6911 (255-12931)	20351 (550-38945)	87696 (1306-829743)
**DP15SZ15**	60 (2-491)	64 (34-177)	164 (2-1507)	0 (0-0)	139 (59-254)	6823 (205-12786)	20058 (521-37916)	84029 (1167-783622)
**PZ5**	64 (2-644)	63 (35-164)	61 (2-638)	0 (0-0)	151 (62-360)	7806 (281-19692)	22873 (598-58796)	26083 (1458-247955)

**SZ15**	64 (2-655)	63 (35-165)	62 (2-650)	0 (0-0)	145 (62-298)	6969 (271-13125)	20711 (525-39520)	26209 (1426-249037)
**CH**	63 (2-542)	64 (35-148)	85 (2-684)	0 (0-0)	152 (59-285)	8304 (272-14464)	24375 (585-44671)	33896 (1343-260430)
**PV1km14d**	58 (2-614)	62 (35-161)	57 (2-608)	630 (0-3116)	151 (62-365)	7541 (272-19483)	20914 (535-52343) / 13803 (0-103230)	24618 (1213-242252)
**PV1km25IH**	69 (2-582)	64 (36-154)	67 (2-573)	865 (0-3032)	175 (63-356)	8514 (274-19192)	21535 (550-54663) / 43156 (0-86934)	27522 (1556-222614)
**PV1km25PC**	70 (2-724)	65 (35-181)	68 (2-721)	0 (0-0)	147 (62-321)	7224 (193-13914)	21145 (512-42456)	29718 (1556-273456)
**PV3km14d_bov**	67 (3-597)	63 (36-158)	66 (2-594)	317 (0-4523)	152 (64-365)	6896 (307-14180)	18670 (514-28836) / 6745 (0-1683559	26805 (1557-235960)
**PV3km14d_sui**	63 (3-615)	64 (36-164)	60 (2-609)	178 (0-1070)	145 (62-311)	7031 (306-13434)	19773 (620-37822) / 8321 (0-82604)	25880 (1552-238906)
**PV3km14d_ovi**	56 (2-629)	61 (35-164)	54 (2-625)	133 (0-2795)	141 (62-338)	6574 (271-14083)	18661 (514-37116) / 1052 (0-41960)	23391 (1295-241843)
**SV3km14d**	61 (2-606)	64 (36-169)	59 (2-605)	323 (0-3464)	189 (62-1256)	7102 (276-16390)	20772 (587-46838)	26545 (1453-240083) / 191320 (0-2010723)
**SV3km25IH**	57 (2-606)	63 (36-159)	57 (2-599)	853 (0-3449)	359 (63-1245)	6265 (298-16941)	18376 (636-48229)	24926 (1556-229674) / 502259 (0-2010822)
**SV3km14d_bov**	56 (2-460)	61 (35-142)	54 (2-457)	121 (0-2328)	135 (60-536)	5156 (282-8598)	15291 (614-24887)	22486 (1464-177328) / 24285 (0-454574)
**North_Ruminant (SZ 1)**								
**Basic**	1 (0-14)	30 (0-45)	1 (0-14)	0 (0-0)	58 (1-88)	251 (1-2479)	846 (1-7879)	8 (0-6477)
**DP15**	1 (0-14)	30 (0-49)	1 (0-14)	0 (0-0)	58 (1-89)	251 (1-2152)	846 (1-6963)	7 (0-6843)
**DP15SZ15**	1 (0-15)	30 (0-48)	1 (0-20)	0 (0-0)	58 (1-94)	254 (1-2700)	863 (1-8648)	8 (0-8633)
**PZ5**	1 (0-18)	30 (0-48)	1 (0-18)	0 (0-0)	58 (1-99)	254 (1-3462)	844 (1-11175)	8 (0-5928)
**SZ15**	1 (0-15)	30 (0-45)	1 (0-15)	0 (0-0)	58 (1-92)	253 (1-2686)	863 (1-8694)	8 (0-6438)

**CH**	1 (0-16)	30 (0-47)	1 (0-22)	0 (0-0)	56 (1-101)	256 (1-3996)	864 (1-13589)	8 (0-9617)
**PV1km14d**	1 (0-16)	30 (0-45)	1 (0-16)	0 (0-68)	58 (1-93)	251 (1-2862)	844 (1-8641) / 0 (0-793)	7 (0-6482)
**PV1km25IH**	1 (0-14)	30 (0-46)	1 (0-14)	0 (0-0)	58 (1-92)	254 (1-2713)	857 (1-8548)	8 (0-5627)
**PV1km25PC**	1 (0-15)	30 (0-45)	1 (0-14)	0 (0-0)	58 (1-89)	252 (1-2525)	858 (1-7860)	8 (0-5291)
**PV3km14d_bov**	1 (0-20)	30 (0-47)	1 (0-20)	0 (0-110)	58 (1-103)	253 (1-3224)	842 (1-8961) / 0 (0-4484)	8 (0-7317)
**PV3km14d_sui**	1 (0-17)	30 (0-47)	1 (0-17)	0 (0-21)	58 (1-93)	253 (1-2584)	861 (1-8793) / 0 (0-229)	8 (0-5981)
**PV3km14d_ovi**	1 (0-13)	30 (0-44)	1 (0-13)	0 (0-30)	58 (1-87)	252 (1-2022)	852 (1-6865) / 0 (0-430)	7 (0-4800)
**SV3km14d**	1 (0-11)	30 (0-44)	1 (0-11)	0 (0-29)	58 (1-89)	252 (1-1856)	847 (1-5861)	8 (0-4182) / 0 (0-21287)
**SV3km25IH**	1 (0-15)	30 (0-46)	1 (0-15)	0 (0-0)	58 (1-94)	253 (1-2559)	855 (1-8717)	8 (0-5819) / 0 (0-0)
**SV3km14d_bov**	1 (0-14)	30 (0-47)	1 (0-14)	0 (0-16)	56 (1-89)	253 (1-2943)	853 (1-8772)	8 (0-6565) / 0 (0-3043)
**Central_Ruminant (SZ 1)**								
**Basic**	1 (0-17)	30 (0-47)	1 (0-17)	0 (0-0)	58 (1-99)	231 (1-3207)	665 (1-10243)	8 (0-7086)
**DP15**	1 (0-19)	30 (0-49)	1 (0-30)	0 (0-0)	58 (1-99)	230 (1-3281)	662 (1-9685)	8 (0-12619)
**DP15SZ15**	1 (0-19)	30 (0-52)	1 (0-31)	0 (0-0)	58 (1-101)	230 (1-3771)	670 (1-10858)	7 (0-11024)
**PZ5**	1 (0-21)	30 (0-48)	1 (0-21)	0 (0-0)	58 (1-103)	230 (1-3828)	657 (1-11022)	8 (0-7089)
**SZ15**	1 (0-23)	30 (0-51)	1 (0-23)	0 (0-0)	58 (1-107)	232 (1-3931)	676 (1-12248)	8 (0-5362)
**CH**	1 (0-17)	30 (0-48)	1 (0-26)	0 (0-0)	56 (1-107)	232 (1-4613)	665 (1-14051)	7 (0-10139)

**PV1km14d**	1 (0-14)	30 (0-47)	1 (0-14)	0 (0-99)	58 (1-95)	230 (1-2898)	659 (1-8753) / 0 (0-952)	7 (0-6062)
**PV1km25IH**	1 (0-18)	30 (0-47)	1 (0-18)	0 (0-0)	58 (1-98)	230 (1-2935)	660 (1-9188)	7 (0-6736)
**PV1km25PC**	1 (0-17)	30 (0-47)	1 (0-17)	0 (0-0)	58 (1-96)	230 (1-2985)	660 (1-9095)	7 (0-6663)
**PV3km14d_bov**	1 (0-13)	30 (0-45)	1 (0-13)	0 (0-72)	58 (1-91)	230 (1-2322)	670 (1-6248) / 0 (0-3164)	7 (0-5512)
**PV3km14d_sui**	1 (0-18)	30 (0-47)	1 (0-17)	0 (0-26)	58 (1-96)	230 (1-3144)	676 (1-9670) / 0 (0-535)	8 (0-7539)
**PV3km14d_ovi**	1 (0-22)	30 (0-49)	1 (0-21)	0 (0-65)	58 (1-103)	231 (1-3678)	674 (1-11210) / 0 (0-1075)	8 (0-8411)
**SV3km14d**	1 (0-17)	30 (0-46)	1 (0-17)	0 (0-52)	58 (1-97)	231 (1-3233)	670 (1-9903)	8 (0-6725) / 0 (0-29745)
**SV3km25IH**	1 (0-14)	30 (0-45)	1 (0-14)	0 (0-0)	58 (1-91)	229 (1-2658)	655 (1-8350)	7 (0-5362) / 0 (0-0)
**SV3km14d_bov**	1 (0-14)	30 (0-46)	1 (1-13)	0 (0-18)	56 (1-86)	230 (1-3491)	664 (1-10142)	7 (0-7209) / 0 (0-4664)
**South_Ruminant (SZ 1)**								
**Basic**	1 (0-17)	30 (0-49)	1 (0-17)	0 (0-0)	58 (1-99)	277 (1-3134)	766 (1-8872)	11 (0-8606)
**DP15**	1 (0-23)	30 (0-52)	1 (0-44)	0 (0-0)	58 (1-105)	281 (1-3950)	789 (1-11974)	11 (0-16718)
**DP15SZ15**	1 (0-22)	30 (0-53)	1 (0-43)	0 (0-0)	58 (1-104)	280 (1-3854)	786 (1-11350)	11 (0-17214)
**PZ5**	1 (0-20)	30 (0-49)	1 (0-20)	0 (0-0)	58 (1-101)	283 (1-3776)	771 (1-10968)	10 (0-8689)
**SZ15**	1 (0-32)	30 (0-53)	1 (0-32)	0 (0-0)	58 (1-118)	280 (1-4987)	780 (1-14151)	12 (0-12918)
**CH**	1 (0-22)	30 (0-51)	1 (0-32)	0 (0-0)	56 (1-117)	283 (1-5320)	794 (1-15761)	12 (0-12867)
**PV1km14d**	1 (0-22)	30 (0-49)	1 (0-22)	0 (0-182)	58 (1-107)	280 (1-4002)	776 (1-11671) / 0 (0-2135)	12 (0-10143)

**PV1km25IH**	1 (0-29)	30 (0-55)	1 (0-28)	0 (0-269)	58 (1-117)	279 (1-4626)	784 (1-13699) / 0 (0-3086)	13 (0-9953)
**PV1km25PC**	1 (0-26)	30 (0-52)	1 (0-26)	0 (0-0)	58 (1-112)	281 (1-4409)	787 (1-11805)	12 (0-10818)
**PV3km14d_bov**	1 (0-25)	30 (0-50)	1 (0-24)	0 (0-190)	58 (1-112)	278 (1-4105)	769 (1-10573) / 0 (0-7745)	11 (0-9611)
**PV3km14d_sui**	1 (0-26)	30 (0-51)	1 (0-25)	0 (0-39)	58 (1-109)	279 (1-4132)	777 (1-11848) / 0 (0-942)	10 (0-8942)
**PV3km14d_ovi**	1 (0-28)	30 (0-50)	1 (0-28)	1 (0-27)	58 (1-112)	279 (1-4476)	789 (1-12467) / 0 (0-1969)	11 (0-11142)
**SV3km14d**	1 (0-27)	30 (0-51)	1 (0-27)	0 (0-111)	58 (1-118)	278 (1-4611)	773 (1-13251)	11 (0-10734) / 0 (0-58425)
**SV3km25IH**	1 (0-25)	30 (0-50)	1 (0-24)	0 (0-0)	58 (1-106)	280 (1-3970)	781 (1-11115)	11 (0-9742) / 0 (0-0)
**SV3km14d_bov**	1 (0-25)	30 (0-50)	1 (0-25)	0 (0-38)	56 (1-104)	280 (1-3896)	776 (1-10493)	13 (0-9570) / 0 (0-8125)
**Zealand_Ruminant (SZ 1)**								
**Basic**	1 (0-2)	30 (0-36)	1 (0-2)	0 (0-0)	58 (1-64)	199 (1-325)	413 (1-842)	6 (0-353)
**DP15**	1 (0-2)	30 (0-36)	1 (0-2)	0 (0-0)	58 (1-64)	199 (1-340)	412 (1-818)	6 (0-216)
**DP15SZ15**	1 (0-2)	30 (0-36)	1 (0-2)	0 (0-0)	58 (1-64)	202 (1-396)	417 (1-871)	6 (0-418)
**PZ5**	1 (0-2)	30 (0-36)	1 (0-2)	0 (0-0)	58 (1-63)	201 (1-377)	413 (1-850)	6 (0-268)
**SZ15**	1 (0-2)	30 (0-36)	1 (0-2)	0 (0-0)	58 (1-63)	199 (1-324)	417 (1-813)	6 (0-420)
**CH**	1 (0-2)	30 (0-34)	1 (0-2)	0 (0-0)	56 (1-60)	199 (1-363)	423 (1-843)	6 (0-257)
**PV1km14d**	1 (0-2)	30 (0-36)	1 (0-2)	0 (0-0)	58 (1-64)	199 (1-353)	416 (1-872)	6 (0-328)
**PV1km25IH**	1 (0-2)	30 (0-36)	1 (0-2)	0 (0-0)	58 (1-64)	198 (1-354)	413 (1-835)	6 (0-255)
**PV1km25PC**	1 (0-2)	30 (0-36)	1 (0-2)	0 (0-0)	58 (1-64)	198 (1-341)	414 (1-850)	6 (0-773)

**PV3km14d_bov**	1 (0-2)	30 (0-36)	1 (0-2)	0 (0-0)	58 (1-64)	199 (1-325)	414 (1-830)	6 (0-322)
**PV3km14d_sui**	1 (0-2)	30 (0-36)	1 (0-2)	0 (0-0)	58 (1-64)	199 (1-329)	413 (1-850)	6 (0-440)
**PV3km14d_ovi**	1 (0-2)	30 (0-36)	1 (0-2)	0 (0-0)	58 (1-65)	200 (1-343)	414 (1-875)	6 (0-441)
**SV3km14d**	1 (0-3)	30 (0-36)	1 (0-3)	0 (0-0)	58 (1-64)	200 (1-388)	418 (1-843)	7 (0-509) / 0 (0-0)
**SV3km25IH**	1 (0-2)	30 (0-36)	1 (0-2)	0 (0-0)	58 (1-64)	200 (1-353)	413 (1-840)	6 (0-365) / 0 (0-0)
**SV3km14d_bov**	1 (0-2)	30 (0-35)	1 (0-2)	0 (0-0)	56 (1-61)	199 (1-322)	410 (1-808)	6 (0-428) / 0 (0-0)
**Entire_DK_Ruminant (SZ 2)**								
**Basic**	1 (1-7)	30 (30-45)	1 (1-7)	0 (0-0)	58 (57-80)	266 (97-1340)	708 (187-3862)	140 (44-3228)
**DP15**	1 (1-7)	30 (30-44)	1 (1-7)	0 (0-0)	56 (55-76)	268 (97-1313)	731 (187-3626)	139 (45-3940)
**DP15SZ15**	1 (1-6)	30 (30-44)	1 (1-6)	0 (0-0)	56 (55-74)	268 (97-1197)	736 (186-3569)	133 (43-3474)
**PZ5**	1 (1-7)	31 (30-44)	1 (1-7)	0 (0-0)	59 (57-80)	279 (104-1250)	750 (175-3702)	139 (45-3463)
**SZ15**	1 (1-7)	30 (30-43)	1 (1-7)	0 (0-0)	58 (57-76)	267 (96-1214)	726 (177-3415)	138 (43-2981)
**CH**	1 (1-7)	31 (30-44)	1 (1-11)	0 (0-0)	56 (55-84)	277 (97-2231)	762 (177-6582)	145 (45-4204)
**PV1km14d**	1 (1-7)	30 (30-43)	1 (1-7)	0 (0-40)	59 (57-79)	278 (109-1364)	765 (244-4051)	129 (44-3560)
**PV1km25IH**	1 (1-11)	30 (30-45)	1 (1-11)	0 (0-0)	59 (57-88)	276 (201-5730)	745 (201-5730)	139 (45-4329)
**PV1km25PC**	1 (1-9)	30 (30-43)	1 (1-9)	0 (0-0)	59 (57-82)	277 (104-1633)	771 (201-4653)	150 (45-3458)
**PV3km14d_bov**	1 (1-6)	30 (30-43)	1 (1-6)	0 (0-13)	59 (57-77)	275 (103-1131)	771 (203-3569)	139 (43-2982)

**PV3km14d_sui**	1 (1-9)	30 (30-44)	1 (1-9)	0 (0-14)	59 (57-81)	271 (104-1587)	729 (177-4730)	139 (43-3902)
**PV3km14d_ovi**	1 (1-6)	30 (30-42)	1 (1-6)	0 (0-9)	58 (57-76)	272 (104-1068)	745 (206-3168)	129 (44-2903)
**SV3km14d**	1 (1-6)	31 (30-43)	1 (1-6)	0 (0-16)	59 (57-79)	270 (96-1196)	743 (186-3422)	131 (45-3188) / 0 (0-8584)
**SV3km25IH**	1 (1-7)	30 (30-43)	1 (1-7)	0 (0-0)	59 (57-75)	270 (97-1260)	726 (187-3534)	120 (43-3095) / 0 (0-0)
**SV3km14d_bov**	1 (1-7)	30 (30-44)	1 (1-7)	0 (0-8)	56 (55-76)	268 (103-1419)	710 (201-4440)	139 (47-3058) /0 (0-854)

Table S4: Economic outcomes of the simulation model. Values shown as Median (5th and 95th percentiles)

**Mitigation measures stratified by epidemic initiation scenarios**	**Surveillance**	**Culling**	**Disposal**	**Cleaning_and_ Disinfection**	**Vaccination**	**Control_ Center**	**Compensation**	**Post-Outbreakmanagement**	**Production losses**	**Total_ Direct_Losses**	**Total_ Indirect_Losses**	**Total_ Economic_Losses**
**North_Cattle (SZ 1)**												
**Basic**	220119 (0-9691780)	2965 (0-2405785)	8360 (0-9330777)	567568 (0-95110199)	0 (0-0)	456101 (0-2068365)	112557 (0-123294489)	910736 (0-28709313)	6744 (0-6859140)	2351934 (0-271939592)	2715124762 (0-9603551423)	2719106044 (0-9886169576)
**DP15**	215659 (0-8244013)	2791 (0-5933220)	9221 (0-18244374)	567568 (0-202025777)	0 (0-0)	456101 (0-2387105)	123421 (0-215148423)	967652 (0-41791136)	7212 (0-47293939)	2553955 (0-541369246)	2696235409 (0-11287875244)	2699418242 (0-11856439059)
**DP15SZ15**	238329 (0-9119599)	5899 (0-6914614)	18516 (0-21205964)	648649 (0-235463641)	0 (0-0)	456101 (0-1994646)	211295 (0-243927148)	1026538 (0-29533423)	15579 (0-57569100)	2902974 (0-609269722)	2743351709 (0-12292718678)	2746710308 (0-12875488625)
**PZ5**	284049 (0-10532189)	8378 (0-2840110)	30007 (0-11204092)	770271 (0-106019667)	0 (0-0)	466708 (0-2642734)	367125 (0-145387234)	1281683 (0-44706454)	26016 (0-8476415)	3146330 (0-338004464)	2739236143 (0-10962915551)	2743096087 (0-11305216277)
**SZ15**	255387 (0-9011694)	4043 (0-2254096)	13192 (0-9167246)	648649 (0-85627763)	0 (0-0)	456101 (0-1963356)	163474 (0-119441808)	931962 (0-29253044)	15716 (0-7204884)	2689690 (0-260468551)	2754271884 (0-10093449303)	2757108986 (0-10348941072)
**CH**	248635 (0-8573558)	10346 (0-2959081)	34404 (0-11460219)	851352 (0-113760889)	0 (0-0)	434887 (0-1994116)	354440 (0-147549616)	1419474 (0-31001430)	25570 (0-8386345)	3362920 (0-329282904)	2738019378 (0-9569719156)	2741304294 (0-9874696397)
**PV1km14d**	227650 (0-9332566)	4614 (0-2347163)	14876 (0-9112926)	648649 (0-93310199)	0 (0-15710845)	456101 (0-2460824)	180628 (0-120510936)	956384 (0-41591808)	11568 (0-6904059)	2663869 (0-294916058)	2722896724 (0-16654881062)	2724960846 (0-16956633537)
**PV1km25IH**	252958 (0-10115628)	6079 (0-2488463)	18891 (0-10062761)	729730 (0-97566959)	0 (0-15672603)	466708 (0-2302249)	232338 (0-130550342)	1108393 (0-37805791)	17444 (0-7598483)	2972012 (0-308614451)	2733891068 (0-16607765608)	2736283164 (0-16931813968)
**PV1km25PC_**	236492 (0-11544635)	5652 (0-2999719)	15241 (0-11986551)	648649 (0-111182509)	0 (0-0)	456101 (0-2206256)	194814 (0-158477684)	1057612 (0-29381876)	16439 (0-8496767)	2711278 (0-339554491)	2717117090 (0-10887171444)	2719199501 (0-11208776616)
**PV3km14d_bov**	229991 (0-9131830)	4754 (0-2202377)	13676 (0-8399300)	567568 (0-82643976)	0 (0-7344904)	466708 (0-2980567)	172438 (0-109421003)	1078299 (0-50333112)	15436 (0-7357632)	2699901 (0-310172282)	2724504592 (0-17252901538)	2726608473 (0-17530694967)
**PV3km14d_sui**	241009 (0-10173680)	5530 (0-2625477)	17255 (0-10431088)	648649 (0-96095334)	0 (0-15517368)	466708 (0-2238077)	213316 (0-136646620)	1011316 (0-35961749)	17608 (0-6613335)	2726513 (0-272491165)	2708662616 (0-16314482707)	2711063400 (0-16650855615)
**PV3km14d_ovi**	239736 (0-9121548)	5464 (0-2325117)	13388 (0-9266600)	648649 (0-86187221)	0 (0-2328654)	466708 (0-2397712)	176209 (0-120862968)	1147836 (0-34302062)	17608 (0-6613335)	2726513 (0-272491165)	2739757614 (0-16331124404)	2743174320 (0-16596437735)
**SV3km14d**	238093 (0-10753039)	4127 (0-2458194)	13524 (0-9697190)	648649 (0-93537227)	0 (0-15691989)	456101 (0-12718854)	162541 (0-128260161)	1009591 (0-670183895)	11239 (0-5277799491)	2745236 (0-6245144661)	2721201944 (0-9458977658)	2723055614 (0-15288889683)
**SV3km25IH**	203570 (0-11005003)	3330 (0-2512459)	10050 (0-10189366)	587838 (0-95969662)	0 (0-15687553)	456101 (0-12761282)	119137 (0-130396138)	988254 (0-658236463)	9434 (0-5358374044)	2595134 (0-6323702349)	2695892745 (0-9802260157)	2798682158 (0-16210101486)

**SV3km14d_bov**	238862 (0-9191398)	5305 (0-2062102)	16040 (0-8383054)	648649 (0-82435192)	0 (0-2066980)	434887 (0-3427122)	196486 (0-109425145)	1111142 (0-346761862)	12114 (0-177388619)	2762596 (0-745978985)	2753663502 (0-9745403380)	2756966933 (0-10509551582)
**Central_Cattle (SZ 1)**												
**Basic**	529777 (0-11566231)	34298 (0-2930406)	120042 (0-11549699)	1702704 (0-111821022)	0 (0-0)	540957 (0-2228531)	1510182 (0-152913648)	2566700 (0-30435197)	95034 (0-8817066)	6995634 (0-331866620)	2925443086 (0-11198244687)	2934312160 (0-11523052797)
**DP15**	482820 (0-9952796)	26320 (0-7549403)	86066 (0-23066453)	1297298 (0-259518384)	0 (0-0)	514440 (0-2069426)	1053429 (0-273388507)	1995230 (0-30116006)	79155 (0-66430554)	5446304 (0-660107127)	2882240588 (0-12376761146)	2886125682 (0-13060762552)
**DP15SZ15**	574040 (0-10364138)	35592 (0-7859343)	121533 (0-24571997)	1500001 (0-276506901)	0 (0-0)	551564 (0-2123521)	1464694 (0-289651126)	2581784 (0-31094374)	93772 (0-72756684)	6962630 (0-719794704)	3029560696 (0-13760837663)	3034878911 (0-14485759752)
**PZ5**	589002 (0-12264187)	32954 (0-3276409)	113153 (0-12957709)	1439190 (0-131700091)	0 (0-0)	540957 (0-2938139)	1394315 (0-166260949)	2171208 (0-47758133)	88290 (0-9982992)	6424527 (0-391603021)	2928113227 (0-12663041381)	2934718106 (0-13038201094)
**SZ15**	703185 (0-11485465)	46728 (0-3173570)	162866 (0-12551836)	2270272 (0-119215623)	0 (0-0)	578082 (0-2216863)	1990784 (0-163651718)	3074445 (0-31060473)	128580 (0-9190191)	9471452 (0-352625806)	3113027002 (0-11769330807)	3121195276 (0-12113122869)
**CH**	413764 (0-11036583)	38597 (0-3963410)	134724 (0-15633752)	1702704 (0-158779164)	0 (0-0)	509136 (0-2365361)	1718828 (0-205418634)	2759988 (0-33935162)	104400 (0-11368380)	7418417 (0-444592115)	2901547340 (0-11771018430)	2905633552 (0-12178269960)
**PV1km14d**	586018 (0-12414490)	37586 (0-3274393)	132326 (0-13277592)	1581082 (0-127538601)	0 (0-15736266)	556868 (0-2673494)	1682218 (0-173020928)	2789743 (0-43062042)	106210 (0-9390953)	7525300 (0-399863199)	2944109038 (0-18477687241)	2949321831 (0-18869790647)
**PV1km25IH**	586127 (0-12064708)	40168 (0-3242253)	134677 (0-12915395)	1722974 (0-123896032)	0 (0-15703638)	540957 (0-2471431)	1571034 (0-167643553)	2409084 (0-39141015)	125645 (0-9536395)	7389314 (0-378232237)	2940173580 (0-18077854252)	2950484602 (0-18472337855)
**PV1km25PC**	453617 (0-12291677)	24423 (0-3148320)	92173 (0-12521829)	1378379 (0-122728465)	0 (0-0)	519743 (0-2281035)	1032999 (0-164584370)	2066918 (0-30789782)	78072 (0-9214447)	5524768 (0-357941365)	2878396150 (0-11674849450)	2881726656 (0-12025515833)
**PV3km14d_bov**	619249 (0-11626371)	33129 (0-3123332)	112912 (0-12503282)	1702704 (0-119126432)	0 (0-9201594)	562171 (0-3533722)	1468524 (0-161656345)	2580449 (0-56363704)	96081 (0-9167394)	7021429 (0-395148632)	2965062786 (0-19745265879)	2971981232 (0-20151035509)
**PV3km14d_sui**	491742 (0-12785346)	27603 (0-3444134)	102019 (0-14058494)	1418920 (0-130443332)	0 (0-15521125)	519743 (0-2525527)	1331372 (0-183143347)	2093588 (0-40112244)	80565 (0-10025606)	6614986 (0-413856706)	2883206731 (0-18394478754)	2886882418 (0-18783813816)
**PV3km14d_ovi**	474554 (0-13029616)	27966 (0-3484671)	95179 (0-13865251)	1418920 (0-135938607)	0 (0-3035571)	530350 (0-2969960)	1102098 (0-179845891)	2202881 (0-39504663)	85092 (0-9970738)	5807686 (0-397827802)	2917338906 (0-19488076009)	2923518776 (0-19902787149)

**SV3km14d**	555239 (0-13725151)	36748 (0-3312001)	125872 (0-13550430)	1682434 (0-124240627)	0 (0-15754673)	562171 (0-12993575)	1539408 (0-177134277)	3223784 (0-697208408)	150035 (0-5431289648)	7588886 (0-6489274017)	2972109718 (0-11789878891)	2980358216 (0-18359904992)
**SV3km25IH**	562398 (0-14363999)	37041 (0-3517851)	136551 (0-14026994)	1540542 (0-139074418)	0 (0-15745284)	530350 (0-12940540)	1564044 (0-181230589)	1999254 (0-690774386)	104775 (0-5435276759)	6900042 (0-6481469044)	2913417229 (0-12052728107)	2939169758 (0-18587312310)
**SV3km14d_bov**	464305 (0-12054783)	28608 (0-2893932)	95783 (0-11961989)	1216217 (0-120523055)	0 (0-3066172)	482619 (0-4627834)	1145845 (0-156999200)	1939230 (0-486914755)	77957 (0-368908237)	5884032 (0-1168871548)	2927011732 (0-11740757695)	2933321680 (0-12857168013)
**South_Cattle (SZ 1)**												
**Basic**	1093622 (0-16254965)	93122 (0-4605419)	366543 (0-18812241)	3831084 (0-175137282)	0 (0-0)	689455 (0-2716983)	4786626 (0-247857389)	5664102 (0-34519005)	282697 (0-13449334)	16596658 (0-511402358)	3257661190 (0-14226823970)	3273797248 (0-14735440898)
**DP15**	1072438 (0-13033681)	104447 (0-10180450)	382209 (0-31432520)	3810813 (0-360004245)	0 (0-0)	721276 (0-2450217)	4997828 (0-369718018)	5946636 (0-34206780)	291433 (0-113022389)	17348878 (0-927674784)	3302609407 (0-15838812409)	3321345321 (0-16767811984)
**DP15SZ15**	1033678 (0-12895555)	103914 (0-10353981)	382652 (0-32616538)	3810814 (0-372385335)	0 (0-0)	705366 (0-2461354)	5035148 (0-390385046)	5684930 (0-33976635)	280824 (0-117787061)	16995250 (0-969880060)	3451635619 (0-16976775203)	3461375682 (0-17939378273)
**PZ5**	1241145 (0-14405672)	125268 (0-4266770)	460920 (0-17228701)	4662166 (0-163520384)	0 (0-0)	784918 (0-3120049)	6026770 (0-223913042)	7747248 (0-49955013)	340329 (0-13236214)	20833984 (0-491144995)	3404798678 (0-14239468156)	3428151812 (0-14725332722)
**SZ15**	1123115 (0-14970673)	122729 (0-4240421)	444166 (0-17223635)	4459462 (0-164558220)	0 (0-0)	731883 (0-2599245)	5719354 (0-224122243)	6831988 (0-33925849)	338905 (0-12098023)	19676331 (0-472501356)	3489551694 (0-14071866298)	3512194667 (0-14549994060)
**CH**	972539 (0-14599767)	136484 (0-5247328)	517503 (0-20739090)	5391896 (0-208745414)	0 (0-0)	763704 (0-2704785)	6793914 (0-271429813)	8921532 (0-37033539)	380378 (0-15517047)	23746968 (0-575097765)	3403713822 (0-14154377034)	3428694638 (0-14737132200)
**PV1km14d**	1080577 (0-16763713)	105847 (0-4729932)	390024 (0-19064651)	3790543 (0-181232555)	227457 (0-15768308)	726580 (0-3086637)	5036681 (0-248484867)	5961998 (0-44718447)	278477 (0-12857789)	18266742 (0-545267943)	8134980382 (0-21681754301)	8155907816 (0-22220406482)
**PV1km25IH**	1227390 (0-14756936)	128175 (0-4059913)	495449 (0-16400831)	4621625 (0-155726456)	0 (0-15744896)	763704 (0-2779564)	6212311 (0-214101707)	6541567 (0-41376571)	369895 (0-11503546)	20491487 (0-476150499)	3376430607 (0-19978898020)	3396219102 (0-20449017768)
**PV1km25PC**	1164720 (0-17099514)	124953 (0-4764999)	456635 (0-19120642)	4621624 (0-181435261)	0 (0-0)	763704 (0-2832069)	5733027 (0-249276737)	7129416 (0-34500563)	348446 (0-13702655)	20988659 (0-523398917)	3370417017 (0-14895007339)	3395141792 (0-15405266902)

**PV3km14d_bov**	1151802 (0-13450936)	112596 (0-3678652)	422147 (0-14724363)	4216219 (0-139869014)	111049 (0-9866177)	758401 (0-3648808)	5464064 (0-191535912)	7085650 (0-57788501)	304520 (0-10359240)	19330264 (0-448568522)	8327332468 (0-21240857383)	8356822928 (0-21687506501)
**PV3km14d_sui**	1104279 (0-14266509)	95107 (0-3761904)	348610 (0-15638658)	4094598 (0-143081855)	181932 (0-15524016)	710669 (0-2715392)	4355085 (0-201697829)	5803002 (0-40213304)	257324 (0-10566809)	16984792 (0-450030490)	8147947938 (0-19707659965)	8165909651 (0-20162722299)
**PV3km14d_ovi**	1269677 (0-14865943)	132235 (0-4076176)	525811 (0-16726182)	5554058 (0-157366324)	37381 (0-3301910)	816739 (0-3257940)	6958323 (0-220594061)	8753226 (0-42262626)	366398 (0-11832124)	23638540 (0-479096934)	8310524254 (0-21114124077)	8323788042 (0-21490760275)
**SV3km14d**	1161655 (0-16512626)	125190 (0-4356746)	476699 (0-17856858)	5270274 (0-164185248)	185303 (0-15759148)	816739 (0-13057217)	6122756 (0-231521638)	17020104 (0-692543669)	3267712 (0-5455383691)	36977785 (0-6526346084)	3421740732 (0-13643667935)	3461633162 (0-20255449683)
**SV3km25IH**	1183624 (0-14977530)	122152 (0-3766019)	463709 (0-15185788)	4439192 (0-150318346)	0 (0-15787162)	747794 (0-13057217)	5988426 (0-197624275)	6874048 (0-722238343)	307642 (0-5486576795)	19670337 (0-6548208943)	3344944635 (0-12741067995)	3359603005 (0-19212854869)
**SV3km14d_bov**	1168586 (0-14390226)	122840 (0-3731032)	481660 (0-15148540)	5108112 (0-148560912)	22777 (0-3790320)	742490 (0-5642924)	6157222 (0-199153046)	12362166 (0-603725596)	480757 (0-541198554)	27697712 (0-1536080798)	3610866432 (0-13456721079)	3638731108 (0-14987260574)
**Zealand_Cattle (SZ 1)**												
**Basic**	51465 (0-3666421)	437 (0-623562)	1408 (0-2339782)	283784 (0-26280423)	0 (0-0)	392459 (0-1549683)	18810 (0-29149078)	492378 (0-25308367)	1124 (0-1862449)	1200824 (0-91165094)	2468346465 (0-5213640386)	2469600727 (0-5315741964)
**DP15**	54041 (0-3096424)	414 (0-1315683)	1320 (0-4045825)	283784 (0-49420972)	0 (0-0)	392459 (0-1474903)	18495 (0-48666350)	492790 (0-24372067)	1061 (0-6609155)	1211766 (0-136470663)	2468306746 (0-5140332966)	2469403361 (0-5280778713)
**DP15SZ15**	48976 (0-3559071)	368 (0-1474655)	1183 (0-4760723)	283784 (0-54993275)	0 (0-0)	392459 (0-1623401)	16383 (0-56954262)	464903 (0-26095274)	937 (0-6679613)	1169245 (0-155446948)	2485822605 (0-6461271005)	2486824085 (0-6609109138)
**PZ5**	49747 (0-4338120)	368 (0-682111)	1165 (0-2855438)	283784 (0-28621641)	0 (0-0)	392459 (0-1728941)	16826 (0-38931679)	470087 (0-29326367)	937 (0-1905541)	1181749 (0-113231797)	2467869843 (0-6011615815)	2469149824 (0-6127956652)
**SZ15**	50235 (0-3078418)	391 (0-552636)	1175 (0-2042382)	283784 (0-21841231)	0 (0-0)	392459 (0-1475964)	17262 (0-26425984)	477484 (0-24517988)	999 (0-1513410)	1155718 (0-81600044)	2486616975 (0-5798849179)	2487693344 (0-5881687932)
**CH**	49352 (0-3676670)	391 (0-845854)	1183 (0-3331200)	283784 (0-38955432)	0 (0-0)	371245 (0-1612794)	17262 (0-43712421)	479830 (0-28559957)	999 (0-2365477)	1156066 (0-121688951)	2468425902 (0-5925760435)	2469598585 (0-6047394874)
**PV1km14d**	52089 (0-3102961)	391 (0-483221)	1232 (0-1806089)	283784 (0-20073662)	0 (0-2429946)	392459 (0-1401185)	17638 (0-23499209)	479725 (0-22884608)	999 (0-1301308)	1180760 (0-77429256)	2467830124 (0-10888640983)	2468988082 (0-10961059784)
**PV1km25IH**	52716 (0-3699433)	379 (0-538123)	1155 (0-2260767)	283784 (0-23335151)	0 (0-8878747)	392459 (0-1701893)	17262 (0-30292030)	478668 (0-28311933)	952 (0-1579973)	1183236 (0-97914145)	2468147872 (0-11927990628)	2469311326 (0-12010620469)
**PV1km25PC**	51433 (0-4187619)	395 (0-660891)	1232 (0-2635597)	283784 (0-26400019)	0 (0-0)	392459 (0-1580443)	17653 (0-34618917)	480237 (0-24893675)	1061 (0-1671840)	1174082 (0-97778034)	2467830124 (0-5485698873)	2468938560 (0-5573658804)
**PV3km14d_bov**	48095 (0-3630003)	375 (0-499318)	1144 (0-1847314)	283784 (0-20985826)	0 (0-1138351)	392459 (0-1665299)	16463 (0-24198377)	463039 (0-25401872)	937 (0-1433138)	1156854 (0-82091422)	2467591814 (0-11705813162)	2468782756 (0-11789920009)
**PV3km14d_sui**	50110 (0-3328664)	414 (0-500398)	1278 (0-2028752)	283784 (0-22641907)	0 (0-2681886)	392459 (0-1474903)	18495 (0-26933725)	480179 (0-24287570)	1061 (0-1490739)	1182084 (0-84527195)	2467949280 (0-11232929605)	2469225238 (0-11319781454)

**PV3km14d_ovi**	48001 (0-3664912)	348 (0-565178)	1122 (0-2231142)	283784 (0-24735827)	0 (0-382536)	392459 (0-1559759)	16029 (0-29316462)	466218 (0-25079422)	906 (0-1645437)	1155310 (0-93082863)	2467710969 (0-11485148578)	2468870068 (0-11566988937)
**SV3km14d**	49873 (0-2878786)	391 (0-475197)	1232 (0-1838156)	283784 (0-21046636)	0 (0-1625178)	392459 (0-1910851)	17262 (0-24221509)	484305 (0-96153891)	937 (0-75360380)	1180514 (0-221475850)	2467949280 (0-4928408143)	2469192171 (0-5144243749)
**SV3km25IH**	48628 (0-3855335)	391 (0-628395)	1232 (0-2400321)	283784 (0-23695961)	0 (0-4941756)	392459 (0-4330308)	17262 (0-31699892)	475654 (0-231907055)	999 (0-558292747)	1169894 (0-844540571)	2467790406 (0-5071521751)	2468957319 (0-5919477224)
**SV3km14d_bov**	52443 (0-2788862)	406 (0-462532)	1232 (0-1793770)	283784 (0-20073663)	0 (0-206614)	371245 (0-1357696)	18495 (0-22693211)	481965 (0-55191075)	1061 (0-5855263)	1163142 (0-109807394)	2486060916 (0-5408353173)	2487286146 (0-5512569139)
**Entire_DK_Dairy (SZ 2)**												
**Basic**	5817562 (1404800-21035012)	1197270 (143785-6335728)	4708046 (548617-25725087)	45891924 (6484464-237423809)	0 (0-0)	1686513 (848560-3193768)	61937493 (7206708-337243850)	25178408 (8820206-38824419)	3421650 (377863-18634090)	150434554 (26129219-681718046)	6745024000 (3522130028-17692141248)	6894703737 (3556677571-18369038681)
**DP15**	5433228 (1278426-19005594)	2840125 (225625-15155131)	8818693 (746864-47308645)	102486542 (8874329-538449604)	0 (0-0)	1617568 (816209-3065953)	105062054 (9793453-554734780)	24965854 (9737917-39692011)	14440647 (727408-222376076)	264752934 (34759041-1439449334)	6912528140 (3616179782-22584818779)	7155401002 (3662103926-24032510615)
**DP15SZ15**	5309869 (1365656-16993759)	2925229 (248441-14394033)	9066472 (816190-44513671)	106277086 (10084466-504833371)	0 (0-0)	1633478 (827346-2875027)	106324228 (10422822-516024831)	25496610 (9815866-38693861)	15905185 (1000727-211366088)	274691878 (36338487-1351190572)	7916815360 (4004655927-21881505679)	8198298778 (4043602339-23232414110)
**PZ5**	5509184 (1337980-18679729)	1103334 (147051-5585963)	4328024 (593993-22728362)	43013543 (6482437-217733259)	0 (0-0)	1888046 (848560-3648808)	56929786 (7872301-296333024)	32471776 (9568149-54995439)	3108851 (411954-16658448)	147781599 (27048549-634314802)	6712245125 (3462533011-17929225887)	6855588324 (3496744329-18562631848)
**SZ15**	5521154 (1324868-18401408)	1159969 (151347-5584056)	4597870 (564571-22658309)	45243274 (5876355-214818390)	0 (0-0)	1675906 (816739-2940260)	60167986 (6800615-295874848)	26028091 (9169454-37437172)	3272013 (432313-16458635)	148160688 (24676771-620015515)	7485428618 (3752025338-16611131234)	7629444939 (3777486216-17183875557)
**CH**	5277287 (1189584-19519274)	1557437 (221437-7378263)	6016355 (791944-29449544)	63243287 (8616898-294438040)	0 (0-0)	1665299 (900534-3267486)	78891728 (10295264-383849558)	27034023 (12279409-42563665)	4499233 (592032-22444676)	186535362 (36440709-804921215)	7149864160 (3549432007-17528338132)	7334687279 (3584887059-18290837995)
**PV1km14d**	5506325 (1422219-18860999)	1154082 (182274-5549593)	4493660 (687345-22766932)	44614895 (7451356-219217042)	10932287 (479052-15799684)	2280505 (922809-3648808)	58967018 (8738044-295778587)	41313440 (11128622-63664918)	3284906 (508165-16322391)	171775841 (31868977-656343547)	13994154615 (8819883486-24115332385)	14158537302 (8852186025-24760491119)
**PV1km25IH**	5815067 (1355537-19731664)	1194134 (163048-5893262)	4742265 (614494-24385662)	46155438 (7210140-228229211)	15590262 (0-15778434)	2248684 (890988-3648808)	62691365 (7696617-318191927)	75861023 (10318076-298343889)	3368306 (424089-17364078)	183814776 (28741488-700292795)	14188667961 (3595939447-24690227386)	14372194139 (3624078771-2536419487)
**PV1km25PC**	5675578 (1391268-22197726)	1152999 (175271-6535084)	4586275 (640538-26029858)	44716248 (7287167-259054230)	0 (0-0)	1675906 (880381-3332719)	60317840 (8175500-339900907)	25149308 (9715718-37875922)	3173825 (490376-20078959)	146382053 (28427729-716507388)	6623910609 (3516645080-18770946189)	6766563762 (3552738250-19488415845)
**PV3km14d_bov**	5615429 (1377286-19052800)	1144385 (147993-5557594)	4547226 (570217-	43844624 (6565545-210867712)	1775876 (57667-7598685)	1909260 (848560-3648808)	59745040 (7117993-283701581)	30194131 (9226388-54436655)	3159176 (395857-16085953)	152113237 (26994733-628994398)	11167227320 (3723134922-122346031286)	11326379796 (3755989049-22858329744)

			21924617)									
**PV3km14d_sui**	5735554 (1347818-19013374)	1225634 (155918-5343859)	4753403 (566551-21866868)	45385167 (6727707-209655552)	10510372 (391126-15530718)	1781976 (880381-3182100)	62217902 (7504818-284314064)	30196488 (9767370-45729488)	3513401 (391976-16602715)	166842524 (28929925-620902522)	6703940842 (3474988398-16279464634)	6872730434 (3500310882-16897942426)
**PV3km14d_ovi**	5586130 (1311744-22269173)	1117592 (156173-6534766)	4443583 (607845-26205330)	43114894 (6687167-250560982)	489841 (7851-2666495)	1755458 (859167-3648808)	58667177 (7812111-342111903)	27076042 (10072870-43178718)	3200563 (413309-19167205)	144862150 (28498657-713460514)	6759218562 (3513556740-18171961225)	6982758222 (3739991018-18846154729)
**SV3km14d**	6065737 (1349052-20062022)	1166267 (200794-5701427)	4674514 (766977-22800373)	44675706 (7416897-214944065)	6033893 (258522-15813295)	4688294 (912202-13227459)	61365497 (9538542-299267286)	259693900 (25050561-737956126)	849563552 (5532770-5531287267)	1288074303 (53266768-6696242750)	6929376769 (3552396853-18016326379)	8314447292 (3606694465-23768818540)
**SV3km25IH**	6199202 (1288010-19665699)	1145926 (155049-5602457)	4560521 (535056-23215383)	43358138 (6281761-213233254)	10113982 (0-15807198)	7424900 (848030-13046610)	59622006 (6543324-303754248)	424345824 (9652616-743972391)	1741516218 (412651-8547898487)	2455584140 (25842375-6609713347)	6728932960 (3449398126-17771048274)	9669133116 (3481612316-22988538106)
**SV3km14d_bov**	5622254 (1369096-20708007)	1153851 (173571-5972253)	4613337 (658424-24830022)	45506788 (7214194-231890021)	985035 (36005-5284486)	1988812 (859167-7503392)	60509114 (8172947-321424584)	175565134 (16189840-806083658)	47369944 (690562-983100734)	349884580 (36361243-2383031452)	7495036272 (3936727716-17730290785)	7864165774 (3962676274-20457109120)
**North_Pig (SZ 1)**												
**Basic**	1277514 (0-11443747)	152862 (0-2999450)	534016 (0-11853541)	6040545 (0-113381835)	0 (0-0)	763704 (0-2174435)	6532020 (0-153292938)	6978144 (0-29682958)	524996 (0-9501857)	23509859 (0-333633110)	3472565206 (0-10827532813)	3495979202 (0-11196808385)
**DP15**	1336880 (0-9758801)	291038 (0-7294285)	942089 (0-22206030)	10763520 (0-256309597)	0 (0-0)	806132 (0-2026998)	11043990 (0-256103733)	7668538 (0-28711296)	1305567 (0-67613502)	35360389 (0-638406958)	3639070793 (0-11887245122)	3676364446 (0-12530861045)
**DP15SZ15**	1282472 (0-8768669)	292759 (0-6841655)	882619 (0-20484497)	10459466 (0-232607556)	0 (0-0)	784918 (0-1973432)	10119079 (0-234600734)	7549744 (0-28490661)	1406180 (0-60353728)	33487305 (0-591002608)	3835309691 (0-11922229648)	3870222178 (0-12556432094)
**PZ5**	1336913 (0-10573463)	157522 (0-2730085)	571566 (0-10908894)	6121626 (0-107914941)	0 (0-0)	795525 (0-2494766)	7179366 (0-140872293)	7267067 (0-39971896)	551256 (0-8636868)	24751312 (0-322481240)	3496952730 (0-10715361150)	3523833428 (0-11035662494)
**SZ15**	1300540 (0-11074162)	155326 (0-3034222)	563465 (0-11458975)	6202707 (0-118465620)	0 (0-0)	790222 (0-2111854)	7179527 (0-147630068)	7437392 (0-29584521)	587005 (0-10295655)	24252610 (0-331961388)	3723178042 (0-11413782569)	3749110592 (0-11761380276)
**CH**	1201286 (0-10255791)	206563 (0-3653588)	748968 (0-13882913)	8189195 (0-140989960)	0 (0-0)	848560 (0-2175496)	9124500 (0-180633298)	10174358 (0-31621537)	784278 (0-11811524)	31755375 (0-387958780)	3594418248 (0-10856271814)	3628627548 (0-11273553131)

**PV1km14d**	1320348 (0-11196128)	150039 (0-2955548)	524224 (0-11961614)	6445950 (0-112791972)	483434 (0-15710623)	784918 (0-2620459)	6490596 (0-155599405)	7235033 (0-41066223)	524473 (0-9433689)	25400276 (0-355226993)	8558587224 (0-17979300822)	8585561912 (0-18325037941)
**PV1km25IH**	1387206 (0-11060914)	172638 (0-2948016)	600853 (0-11180432)	6871626 (0-112033860)	0 (0-15694451)	837953 (0-2377559)	7546732 (0-142728286)	8138222 (0-37884927)	567399 (0-9268071)	26389488 (0-348404084)	3700058802 (0-17504039880)	3723575169 (0-17840263223)
**PV1km25PC**	1334556 (0-12404085)	159588 (0-3391357)	569262 (0-13293333)	6040544 (0-123596030)	0 (0-0)	784918 (0-2302780)	7284934 (0-169447058)	7106630 (0-29259799)	570309 (0-10425872)	24323532 (0-365229280)	3506880564 (0-11622505403)	3530526186 (0-12011465903)
**PV3km14d_bov**	1395723 (0-10816703)	178183 (0-2863728)	629256 (0-10728846)	6709464 (0-107781154)	222869 (0-7959902)	848560 (0-3309384)	7833460 (0-135403338)	8300910 (0-51075742)	589215 (0-8606066)	27192728 (0-348947522)	8887098653 (0-19165399981)	8915562608 (0-19531349021)
**PV3km14d_sui**	1313928 (0-10094866)	159703 (0-2636960)	579903 (0-10252793)	6364869 (0-99518985)	440868 (0-15518725)	795525 (0-2216863)	7133470 (0-132078607)	7712669 (0-35407681)	567682 (0-8779107)	25637828 (0-311514607)	8632070044 (0-16500134890)	8657072968 (0-16804359927)
**PV3km14d_ovi**	1289897 (0-12569276)	153983 (0-3459109)	588439 (0-13682324)	6121625 (0-129796706)	37427 (0-2767849)	795525 (0-2908970)	7390547 (0-176139921)	7374659 (0-36985727)	536621 (0-10822229)	24473546 (0-396194199)	8603973672 (0-19421293095)	8631150428 (0-19826735909)
**SV3km14d**	1240462 (0-13724255)	152750 (0-3379639)	545548 (0-13460081)	6263518 (0-131282522)	250214 (0-15725744)	859167 (0-12993575)	6903191 (0-174933701)	19006714 (0-667051291)	4435768 (0-5427011638)	41421258 (0-6453104181)	3503808354 (0-11637840226)	3550739205 (0-18169992210)
**SV3km25IH**	1388266 (0-12646240)	172753 (0-3086903)	618974 (0-11983426)	6608112 (0-116862242)	0 (0-15734912)	837953 (0-12856745)	7844900 (0-157937840)	8309367 (0-671258324)	815428 (0-5403728246)	26432757 (0-6396014330)	3564278910 (0-10994412427)	3606724294 (0-17473354294)
**SV3km14d_bov**	1282776 (0-10990312)	154350 (0-2833817)	544753 (0-11211576)	6283788 (0-107318991)	38186 (0-2648323)	753097 (0-4096423)	7034946 (0-145701904)	12714570 (0-421434867)	622824 (0-269846938)	29466486 (0-981150554)	3720896322 (0-11143322100)	3746376950 (0-12220062129)
**Central_Pig (SZ 1)**												
**Basic**	1512550 (0-13466816)	211193 (0-3817186)	778567 (0-14524704)	8168924 (0-138040636)	0 (0-0)	869774 (0-2377029)	9773346 (0-190561292)	9268021 (0-30188602)	709545 (0-10920823)	31397884 (0-400488234)	3653651306 (0-12396445120)	3686345842 (0-12795867678)
**DP15**	1577167 (0-11189193)	450956 (0-8669772)	1439187 (0-26629795)	15425685 (0-301715029)	0 (0-0)	901595 (0-2217393)	17015480 (0-310723381)	10361169 (0-30581011)	2124760 (0-83983477)	50389684 (0-783964280)	3837406475 (0-13683250119)	3884286508 (0-14483585268)
**DP15SZ15**	1471012 (0-10309320)	368476 (0-8317461)	1175330 (0-25044544)	13074330 (0-293365692)	0 (0-0)	869774 (0-2143144)	14159076 (0-291412460)	9617108 (0-30371809)	1758858 (0-87163766)	41602610 (0-745306571)	4021788908 (0-14197368178)	4064723786 (0-14942674749)

**PZ5**	1544899 (0-11618084)	221088 (0-3283709)	773787 (0-12691353)	7722978 (0-124060219)	0 (0-0)	880381 (0-2738197)	9887808 (0-163001123)	9729979 (0-44883499)	740226 (0-10034954)	32451790 (0-377999691)	3713036712 (0-11809595348)	3748474563 (0-12176215545)
**SZ15**	1444862 (0-11354633)	194972 (0-3242498)	714050 (0-12462500)	7662167 (0-121646028)	0 (0-0)	848560 (0-2228531)	9156368 (0-159499114)	8536022 (0-30341234)	701402 (0-10418829)	30131950 (0-350699817)	3902364974 (0-11679299097)	3936263744 (0-12029910870)
**CH**	1377530 (0-10014784)	275269 (0-3611124)	995514 (0-14000451)	10459467 (0-138835230)	0 (0-0)	944023 (0-2185042)	12307738 (0-179285496)	12871983 (0-31726078)	944624 (0-10961549)	40926622 (0-393943122)	3740209425 (0-10628081925)	3782359088 (0-11045397333)
**PV1km14d**	1539527 (0-14270778)	232507 (0-4139166)	843884 (0-16200584)	8574330 (0-149908885)	845451 (0-15722504)	928113 (0-2822523)	10511300 (0-207680704)	10236334 (0-42633067)	746586 (0-12552366)	36378922 (0-467760276)	9105385852 (0-20042006794)	9142890141 (0-20535531089)
**PV1km25IH**	1571605 (0-11467975)	225491 (0-2925220)	834386 (0-11522049)	8878384 (0-111326428)	885868 (0-15719555)	922809 (0-2407789)	10630452 (0-149053602)	10587774 (0-38074394)	749893 (0-8999451)	35868592 (0-347989373)	8923840063 (0-17644737868)	8958636330 (0-17991172684)
**PV1km25PC**	1487024 (0-11792688)	198386 (0-3283816)	745110 (0-12878538)	7662168 (0-123833194)	0 (0-0)	848560 (0-2260352)	9240337 (0-166757447)	8883275 (0-29588050)	690617 (0-9915771)	30704066 (0-359187138)	3575443696 (0-11333188332)	3604172304 (0-11728717509)
**PV3km14d_bov**	1501624 (0-12060607)	192581 (0-3295669)	705631 (0-12944523)	7500005 (0-124386570)	275088 (0-9011490)	901595 (0-3554406)	9131896 (0-167698589)	9556197 (0-54763606)	669575 (0-10326245)	30943910 (0-403857320)	9064321924 (0-20268939031)	9093337672 (0-20672264665)
**PV3km14d_sui**	1584598 (0-12136149)	201710 (0-3354261)	763844 (0-12948716)	7783789 (0-124585219)	686977 (0-15518963)	901595 (0-2461354)	9909876 (0-168922203)	9773776 (0-37428219)	705094 (0-10221568)	32411064 (0-389263451)	9101063334 (0-18408310813)	9139639774 (0-18788994646)
**PV3km14d_ovi**	1465558 (0-13493555)	200067 (0-3673521)	701100 (0-14240785)	7479735 (0-137304822)	59741 (0-2992844)	859167 (0-2981097)	8807369 (0-183461222)	8893036 (0-40055143)	658986 (0-11415181)	29676401 (0-415586446)	8800648277 (0-19836038773)	8830782814 (0-20238492706)
**SV3km14d**	1348437 (0-13557393)	195180 (0-3180239)	685175 (0-12407025)	7418924 (0-126101437)	338953 (0-15735623)	922809 (0-12951677)	8836282 (0-159803695)	23245926 (0-668287949)	6519238 (0-5414890642)	52090716 (0-6437686530)	3598536583 (0-11394396314)	3660560252 (0-17848139675)
**SV3km25IH**	1547809 (0-12843043)	207985 (0-3153588)	771679 (0-12430317)	8067573 (0-117713595)	0 (0-15728034)	922809 (0-12876898)	9656074 (0-159534923)	11499382 (0-675051382)	1309866 (0-5408405863)	34500707 (0-6419393926)	3674312214 (0-11013408494)	3740237984 (0-17440802360)
**SV3km14d_bov**	1364770 (0-10506107)	181761 (0-2832066)	664514 (0-11270932)	6972978 (0-102062908)	44081 (0-2732700)	816739 (0-4222116)	8207125 (0-148009818)	15253276 (0-422332270)	1482134 (0-303422118)	36016881 (0-1016058755)	3863771218 (0-10929919587)	3903368059 (0-11840793807)
**South_Pig (SZ 1)**												

**Basic**	1502083 (0-18706370)	204048 (0-5393934)	749302 (0-21547640)	7763518 (0-208573113)	0 (0-0)	869774 (0-2939200)	9728693 (0-280684931)	9325334 (0-34336334)	654473 (0-16754654)	31440780 (0-587711756)	3630237363 (0-15904146297)	3662080173 (0-16491102340)
**DP15**	1606468 (0-16786966)	451671 (0-14012746)	1459349 (0-43819824)	16358118 (0-497059727)	0 (0-0)	912202 (0-2832599)	17779486 (0-518274593)	10255628 (0-36579772)	1978956 (0-199765717)	5058469 (0-1331337600)	3865738408 (0-20498383325)	3905457602 (0-21840392819)
**DP15SZ15**	1560987 (0-15945049)	421026 (0-13832181)	1346634 (0-43003000)	14918927 (0-486900261)	0 (0-0)	906899 (0-2758350)	16098702 (0-503356448)	10828203 (0-36638898)	2023841 (0-203664283)	48813340 (0-1314242041)	4226315768 (0-20770593067)	4286617552 (0-22054102506)
**PZ5**	1520961 (0-18313838)	211198 (0-5689248)	789446 (0-22611412)	7824330 (0-213596090)	0 (0-0)	901595 (0-3575089)	10217142 (0-292843229)	9317014 (0-49745713)	659039 (0-17409474)	32450160 (0-629094444)	3716401960 (0-17167717991)	3745541564 (0-17805945713)
**SZ15**	1624766 (0-17407327)	214742 (0-5231246)	799264 (0-20566639)	8168924 (0-198257568)	0 (0-0)	875078 (0-2780095)	10301451 (0-267342615)	8973541 (0-33617068)	703222 (0-16063695)	32607342 (0-567882380)	4011021460 (0-15858909550)	4043838659 (0-16464279212)
**CH**	1335082 (0-15725177)	257429 (0-6018030)	932892 (0-23518094)	10520277 (0-228229211)	0 (0-0)	912202 (0-2844267)	11889922 (0-304887885)	12245452 (0-35308237)	819457 (0-18115174)	40166564 (0-631394088)	3766616993 (0-14942156825)	3808032487 (0-15570348117)
**PV1km14d**	1635636 (0-18949498)	221252 (0-5603601)	828546 (0-22444429)	8655412 (0-212708254)	850482 (0-15768165)	912202 (0-3342796)	10579706 (0-287582233)	9887029 (0-44210334)	733436 (0-16934901)	35035133 (0-631744634)	9129159706 (0-23754618192)	9166612186 (0-24386362825)
**PV1km25IH**	1601858 (0-18921151)	218760 (0-5547595)	784448 (0-22109153)	8168924 (0-209138654)	154121 (0-15768867)	917506 (0-3203844)	10045599 (0-284016049)	9662732 (0-41522556)	653264 (0-15884822)	32853386 (0-622451846)	8351106322 (0-23540437385)	8381591144 (0-24126185260)
**PV1km25PC**	1532486 (0-19903327)	194885 (0-5920280)	727572 (0-23828490)	7682438 (0-219750150)	0 (0-0)	859167 (0-3066484)	9166240 (0-306779634)	8221757 (0-33436320)	652239 (0-17492643)	29645768 (0-637704368)	3674593628 (0-16667277833)	3707151173 (0-17306396340)
**PV3km14d_bov**	1592826 (0-15599437)	223029 (0-4360111)	826920 (0-17402532)	8250006 (0-164513627)	346270 (0-10083337)	944023 (0-3648808)	10701024 (0-227567913)	10750051 (0-56608020)	695871 (0-13841517)	34838029 (0-513971064)	9241545194 (0-22476881634)	9275582472 (0-23009021642)
**PV3km14d_sui**	1600736 (0-18133677)	217820 (0-5285544)	803488 (0-21121052)	7804060 (0-193834592)	645339 (0-15525000)	880381 (0-3087698)	10442653 (0-273661900)	9829880 (0-42332533)	665961 (0-16068663)	33574274 (0-594393531)	9021096736 (0-22539990408)	9058416601 (0-23113137660)
**PV3km14d_ovi**	1542602 (0-20150787)	207379 (0-5913177)	758647 (0-23627830)	7763518 (0-219798798)	74418 (0-3618177)	890988 (0-3648808)	9706775 (0-305699315)	9081494 (0-40288186)	643462 (0-16704729)	30939704 (0-642619680)	9057838146 (0-24772355243)	9092554369 (0-25414974923)

**SV3km14d**	1460008 (0-18458714)	224373 (0-5216908)	832229 (0-20723256)	8391898 (0-187903506)	374156 (0-15762898)	954630 (0-13173894)	10573671 (0-269976559)	25464492 (0-694519765)	8446762 (0-5517393909)	58323720 (0-6625454336)	3766388508 (0-15250770577)	3823565706 (0-21966131274)
**SV3km25IH**	1619262 (0-19878173)	238264 (0-5588139)	874258 (0-22338863)	9324330 (0-215981903)	410105 (0-15784482)	1012968 (0-13120859)	11095268 (0-290481341)	25348430 (0-708755282)	9705104 (0-5528618593)	69180429 (0-6636707755)	3740240603 (0-15694576006)	3866248011 (0-22438568151)
**SV3km14d_bov**	1486310 (0-17498499)	203801 (0-4934985)	788447 (0-19920838)	7722978 (0-187485938)	56258 (0-4341780)	843257 (0-6216763)	10130884 (0-260567850)	17826638 (0-650787172)	1667971 (0-705150972)	41011362 (0-1873195712)	3955562695 (0-15663064870)	4000223953 (0-17645942264)
**Zealand_Pig (SZ 1)**												
**Basic**	95437 (0-2415421)	4680 (0-468686)	11507 (0-1564747)	364865 (0-17965551)	0 (0-0)	403066 (0-1135479)	99785 (0-18565157)	553624 (0-14965651)	25632 (0-1877653)	1551303 (0-58572256)	2565627767 (0-4293735739)	2567112402 (0-4344889443)
**DP15**	100692 (0-2501454)	5463 (0-1047382)	12916 (0-2932418)	445946 (0-36447986)	0 (0-0)	424280 (0-1157224)	111373 (0-33282080)	556348 (0-16305952)	27318 (0-5650563)	1613418 (0-97202399)	2635080742 (0-4541397269)	2636636109 (0-4649846242)
**DP15SZ15**	105172 (0-2239206)	5895 (0-924183)	13459 (0-2571972)	445946 (0-33577718)	0 (0-0)	413673 (0-1114265)	115447 (0-29730725)	570044 (0-14908229)	27579 (0-5398294)	1645164 (0-88550411)	2647406091 (0-4912360738)	2649003122 (0-4983653812)
**PZ5**	101394 (0-2499663)	5693 (0-413288)	13000 (0-1421144)	445946 (0-15975011)	0 (0-0)	424280 (0-1124872)	114705 (0-17666207)	562012 (0-14645718)	30116 (0-1657034)	1620342 (0-54362701)	2640103744 (0-4356892764)	2641713074 (0-4404648366)
**SZ15**	93296 (0-2440510)	4692 (0-479109)	10842 (0-1539415)	364865 (0-17053389)	0 (0-0)	403066 (0-1156163)	101963 (0-18322707)	554768 (0-15218074)	24508 (0-2035488)	1543824 (0-56170878)	2627398504 (0-4900189350)	2628812723 (0-4945896902)
**CH**	104291 (0-2188196)	6737 (0-571342)	14877 (0-1986514)	445946 (0-23434474)	0 (0-0)	392459 (0-1241019)	125137 (0-24777970)	569439 (0-19815627)	33374 (0-2134027)	1662525 (0-75657242)	2630901942 (0-4551139155)	2632671798 (0-4624330036)
**PV1km14d**	103532 (0-2650303)	5792 (0-482743)	13093 (0-1601932)	445946 (0-17521632)	0 (0-2183559)	424280 (0-1209728)	118382 (0-20302729)	565247 (0-16619459)	27702 (0-1853349)	1628712 (0-63923446)	2636473676 (0-4510323751)	2638241920 (0-4576115577)
**PV1km25IH**	94634 (0-2281423)	5760 (0-420005)	13742 (0-1318411)	445946 (0-15010144)	0 (0-4756784)	403066 (0-1157224)	114676 (0-16482000)	541719 (0-15511200)	27349 (0-1801425)	1587768 (0-57489688)	2624781478 (0-4156526437)	2626875306 (0-4211739025)
**PV1km25PC**	97834 (0-2345372)	5177 (0-447629)	12550 (0-1501856)	445946 (0-17361499)	0 (0-0)	403066 (0-1124342)	113879 (0-18154034)	548438 (0-14433422)	26100 (0-1986222)	1566834 (0-58041724)	2635165162 (0-4324929238)	2636774032 (0-4346808777)
**PV3km14d_bov**	94699 (0-2557116)	4845 (0-472573)	11225 (0-1610884)	445946 (0-18099336)	0 (0-709385)	403066 (0-1230412)	102610 (0-19402204)	551843 (0-16003307)	26399 (0-1891135)	1566580 (0-61323345)	2628749227 (0-4423894952)	2630090869 (0-4488764792)
**PV3km14d_sui**	107025 (0-2402105)	6347 (0-461699)	14577 (0-1471167)	445946 (0-16919604)	0 (0-1602349)	424280 (0-1124872)	119812 (0-18642879)	566242 (0-14925818)	30627 (0-1886774)	1644172 (0-59411752)	2638921861 (0-4335850662)	2641240418 (0-4383414919)
**PV3km14d_ovi**	90901 (0-2574513)	4443 (0-463718)	10000 (0-1564169)	364865 (0-18383120)	0 (0-206453)	403066 (0-1209728)	90029 (0-18329899)	535654 (0-16695748)	21230 (0-1726288)	1535267 (0-60532635)	2546491204 (0-4500549096)	2547851035 (0-4570925024)
**SV3km14d**	95037 (0-2271891)	4395 (0-435773)	10615 (0-1483725)	364865 (0-17489201)	0 (0-876514)	403066 (0-1304661)	93227 (0-17811787)	540832 (0-49366969)	24289 (0-28174502)	1580954 (0-118332977)	2513161714 (0-4334272163)	2564751920 (0-4467976314)
**SV3km25IH**	89667 (0-2489171)	5075 (0-450076)	11550 (0-1515150)	364865 (0-15022307)	0 (0-2755114)	403066 (0-2631597)	108481 (0-18722550)	545494 (0-116384971)	25749 (0-191068258)	1571232 (0-358567952)	2514654346 (0-4281958393)	2566296408 (0-4564690378)
**SV3km14d_bov**	98805 (0-2337671)	5359 (0-456740)	13769 (0-1330372)	364865 (0-17158795)	0 (0-134863)	371245 (0-1103658)	119588 (0-16573872)	547738 (0-36742618)	27599 (0-4969959)	1539636 (0-81789626)	2590853350 (0-4909893612)	2592375722 (0-4997043301)
**Entire_DK_Weaner (SZ 2)**												

**Basic**	2924018 (141359-17902067)	499594 (15560-4991905)	1839940 (32171-19695381)	19114878 (567568-199544731)	0 (0-0)	1294054 (434887-2875558)	23750377 (262887-255950981)	18346467 (712225-33783467)	1599401 (97157-15818147)	69778654 (2138985-552606446)	4687425316 (2680472865-15423821668)	4759688324 (2683355086-15938000917)
**DP15**	2738889 (142279-14920505)	1224480 (15560-11795394)	3571734 (32000-36056943)	42060834 (567568-403942108)	0 (0-0)	1246322 (413673-2609322)	42626130 (240517-417381113)	18402118 (617275-34023461)	6244442 (84939-134355743)	119991180 (2197177-1060948847)	4828642312 (2678913744-17328123381)	4946909600 (2681249157-18367301853)
**DP15SZ15**	2705517 (101462-13509738)	1194271 (12017-11067194)	3530882 (25752-34298777)	41797319 (441892-382228578)	0 (0-0)	1251626 (403066-2492645)	40451718 (222429-400243763)	18047147 (526375-33620382)	6101889 (75774-128887520)	115058294 (1802146-1008730105)	5394677242 (2652724564-17315171632)	5502715726 (2655014971-18324148018)
**PZ5**	2981002 (142504-17962303)	520675 (15998-5400176)	1934987 (34053-21552381)	19966230 (567568-211475821)	0 (0-0)	1378910 (444964-3595773)	24969842 (269654-278264016)	20694393 (682487-51895284)	1718142 (95300-16689837)	73158070 (2382276-608661286)	4768615824 (2698595507-17143192567)	4842296406 (2701746254-17770476911)
**SZ15**	2901673 (139766-17958543)	530305 (15560-5482114)	1935402 (32000-22177448)	20412176 (567568-212896767)	0 (0-0)	1315268 (434887-2939200)	24587561 (257234-288377501)	18487525 (650351-34799310)	1699977 (94016-17472414)	73004850 (2046221-607522191)	5393410905 (2682700181-16388735519)	5455281109 (2684554049-16992940281)
**CH**	2866524 (130761-15673906)	714402 (15560-5558519)	2692980 (36000-21990270)	28378398 (567568-222144070)	0 (0-0)	1389517 (403066-2800778)	34027914 (271850-285396233)	22047156 (686262-38337932)	2221190 (88631-17919815)	97246978 (2380006-610207710)	5136042946 (2666843404-14719586413)	5234998071 (2669148968-15368992718)
**PV1km14d**	2744673 (143638-17856321)	469190 (15560-5259957)	1753465 (33584-21166271)	18324337 (567568-202607572)	2908933 (0-15769869)	1378910 (434887-3648808)	22485016 (269654-273131438)	21049272 (653409-59608965)	1602743 (75714-16388668)	72016431 (2216102-615460537)	10961907677 (2684126520 - 23704693099)	11024438136 (2686930762 - 24298207151)
**PV1km25IH**	3213194 (177302-16360731)	563218 (17843-4854231)	2113026 (38160-19881901)	21871637 (648649-187214317)	9108186 (0-15755128)	1633478 (445494-3554936)	27259468 (306632-255960307)	26374537 (741694-57078938)	1842323 (109152-15359893)	95339984 (2647124-578879002)	11564899050 (2731531412 - 22945010420)	11657546126 (2734752884 - 23513836990)
**PV1km25PC**	3258059 (125197-20897445)	566405 (15920-6012596)	2065723 (35959-23742388)	22398664 (567568-233436643)	0 (0-0)	1336482 (434887-3182100)	26380817 (269654-310156836)	19081009 (487588-36862938)	1935269 (97157-18550455)	78616844 (2141018-662274002)	4916044604 (2694697704-17586623488)	4993311958 (2697203161-18236603568)
**PV3km14d_bov**	3129076 (183190-17317621)	541638 (17253-5013977)	1991763 (35936-20291901)	20756771 (729730-192742023)	531695 (0-7100308)	1389517 (456101-3648808)	25410010 (306825-262821036)	19587328 (809548-49983241)	1736362 (97215-15504148)	75475732 (2676812-578468378)	11080776944 (2726759954 - 23470628707)	11156325996 (2729133883 - 24049097085)
**PV3km14d_sui**	2927807 (181146-18120806)	501320 (15910-5099276)	1919046 (35000-20572859)	19500014 (648649-199135268)	2689889 (0-15525545)	1325875 (444964-3108912)	24864519 (269277-267292088)	19418398 (798368-42905591)	1678164 (97157-16513883)	75043496 (2580337-591342263)	10998649086 (2706573608 - 22882550023)	11075018844 (2708747822-23505737931)
**PV3km14d_ovi**	2717688 (156857-18272857)	465510 (15560-5383614)	1708017 (32000-21370277)	18020282 (567568-203126493)	116870 (0-2349720)	1278144 (434887-3383633)	21909612 (269397-277802339)	17462252 (650179-39427832)	1532432 (82320-16706634)	66226822 (2385393-586997361)	10687427733 (2698351359-23423081000)	10753300258 (2701253742-24019373291)

**SV3km14d**	2643132 (146747-18467534)	513519 (15560-5192951)	1834385 (36162-20289245)	19358122 (567568-192559590)	1503908 (0-15770997)	1776672 (434887-13100175)	23144208 (276004-261307843)	69044818 (714740-710569549)	66176356 (97157-5487195287)	199518492 (2343645-6603387516)	4729433158 (2720102510-16615112978)	4910029020 (2722655231-22940764889)
**SV3km25IH**	3025479 (171104-17995174)	485644 (17952-5070849)	1777731 (38474-20199434)	18263526 (648649-196700811)	3934758 (0-15783370)	3590470 (445494-12982968)	22456313 (299684-259906681)	152356869 (744219-720017074)	372868500000000 (9.725865e+04-5.462318e+09)	616682198 (2536527-6556660717)	4656655242 (2718977002-16126743088)	5296026300 (2721635434-22236791777)
**SV3km14d_bov**	2580514 (151933-14325167)	447013 (15560-3882364)	1620984 (33017-15701385)	17898660 (567568-150987265)	194740 (0-3629561)	1209198 (413673-5465787)	20865972 (281285-205392897)	46466355 (715134-578564722)	6846455 (97127-531002807)	97480890 (2410607-1509669214)	5182235476 (2715408024-13889367440)	5278248754 (2717937176-15496365027)
**North_Ruminant (SZ 1)**												
**Basic**	78481 (0-1111829)	233 (0-136959)	154 (0-483365)	81081 (0-4583111)	0 (0-0)	392459 (0-710669)	2038 (0-6241463)	679940 (0-6566190)	500 (0-409680)	1307026 (0-20186179)	2471444508 (0-3250262450)	2472771216 (0-3263254108)
**DP15**	79057 (0-1196951)	227 (0-108818)	144 (0-440315)	81081 (0-4297300)	0 (0-0)	392459 (0-721276)	1863 (0-5192345)	679397 (0-5735116)	437 (0-456387)	1307628 (0-18469999)	2471325352 (0-3435318275)	2472615741 (0-3447374330)
**DP15SZ15**	82115 (0-1266913)	227 (0-157567)	154 (0-568609)	81081 (0-5803382)	0 (0-0)	392459 (0-774841)	2070 (0-7482341)	686800 (0-7259158)	500 (0-613901)	1311979 (0-22162869)	2492296720 (0-3592597380)	2493484729 (0-3622484821)
**PZ5**	81222 (0-1252565)	247 (0-149673)	150 (0-577642)	81081 (0-6000004)	0 (0-0)	392459 (0-827346)	1863 (0-7674758)	687675 (0-9307714)	500 (0-370805)	1315164 (0-26397550)	2471365071 (0-3488928775)	2472740794 (0-3504678241)
**SZ15**	79913 (0-1160690)	231 (0-135287)	154 (0-487978)	81081 (0-4864868)	0 (0-0)	392459 (0-753627)	2070 (0-5958206)	687024 (0-7184331)	500 (0-411023)	1308752 (0-21819265)	2492098128 (0-3547264604)	2493383420 (0-3572024156)
**CH**	78165 (0-1228236)	248 (0-218198)	162 (0-753652)	81081 (0-8110141)	0 (0-0)	371245 (0-849090)	2070 (0-9757320)	687579 (0-10746943)	500 (0-635570)	1287919 (0-30294586)	2471523945 (0-3560585323)	2472813084 (0-3588607683)
**PV1km14d**	79376 (0-1151405)	225 (0-132578)	144 (0-460826)	81081 (0-5079733)	0 (0-344732)	392459 (0-764234)	1863 (0-6329040)	677107 (0-7703294)	437 (0-405326)	1301362 (0-22997651)	2471126760 (0-3372979073)	2472430690 (0-3385916590)
**PV1km25IH**	79847 (0-1224981)	243 (0-126302)	150 (0-477491)	81081 (0-4866895)	0 (0-0)	392459 (0-754688)	2070 (0-6517216)	686790 (0-7316627)	500 (0-359436)	1313811 (0-21739112)	2471404790 (0-3365212928)	2472751550 (0-3392027955)
**PV1km25PC**	78739 (0-1088036)	242 (0-100600)	154 (0-374482)	81081 (0-4506084)	0 (0-0)	392459 (0-721806)	2070 (0-4927451)	680039 (0-6745600)	500 (0-330373)	1305869 (0-18323521)	2471206197 (0-3298624030)	2472514372 (0-3310994620)
**PV3km14d_bov**	79827 (0-1387242)	231 (0-175419)	162 (0-598815)	81081 (0-6024328)	0 (0-205763)	392459 (0-870304)	2049 (0-7825141)	683868 (0-9232831)	500 (0-462889)	1309204 (0-26700284)	2471285634 (0-3490052295)	2472600014 (0-3525776286)
**PV3km14d_sui**	80015 (0-1267262)	245 (0-124909)	162 (0-412438)	81081 (0-4990544)	0 (0-335506)	392459 (0-764234)	2070 (0-5613565)	678711 (0-7009965)	500 (0-387388)	1309888 (0-21329922)	2471285634 (0-3429439252)	2472609524 (0-3448358189)
**PV3km14d_ovi**	78901 (0-1025015)	225 (0-99303)	144 (0-388349)	81081 (0-3938516)	0 (0-13838)	392459 (0-700592)	1863 (0-5098482)	679368 (0-5465754)	437 (0-305547)	1306159 (0-18092593)	2471206197 (0-3228326002)	2472541041 (0-3253727784)
**SV3km14d**	79142 (0-950006)	244 (0-82011)	150 (0-314538)	81081 (0-3571624)	0 (0-165175)	392459 (0-721276)	2001 (0-4176776)	682666 (0-11494015)	500 (0-2305446)	1305694 (0-23100580)	2471166478 (0-3169163468)	2472503847 (0-3183371334)
**SV3km25IH**	80102 (0-1206707)	252 (0-126321)	162 (0-483842)	81081 (0-4670273)	0 ()	392459 (0-774311)	2070 (0-6556135)	685878 (0-7179518)	500 (0-363641)	1312939 (0-19819349)	2471444508 (0-3329902390)	2472787354 (0-3347628166)
**SV3km14d_bov**	80611 (0-1218144)	231 (0-138605)	150 (0-489218)	81081 (0-4802030)	0 (0-23717)	371245 (0-721276)	1863 (0-6048517)	682186 (0-10738130)	500 (0-932867)	1284304 (0-26508311)	2491859817 (0-3559512230)	2493113478 (0-3588617060)
**Central_Ruminant (SZ 1)**												

**Basic**	81988 (0-1414424)	243 (0-138659)	152 (0-524781)	81081 (0-6004058)	0 (0-0)	392459 (0-828407)	1950 (0-6596355)	615388 (0-8436741)	500 (0-442421)	1222726 (0-23600887)	2469220272 (0-3520334455)	2470434396 (0-3552937321)
**DP15**	81294 (0-1314194)	223 (0-212980)	150 (0-725107)	81081 (0-8154734)	0 (0-0)	392459 (0-828937)	1863 (0-9351248)	616689 (0-8745567)	500 (0-892488)	1226066 (0-30788636)	2469140835 (0-3595527964)	2470398194 (0-3643428096)
**DP15SZ15**	80469 (0-1399887)	225 (0-203057)	150 (0-742724)	81081 (0-8730410)	0 (0-0)	392459 (0-849621)	1863 (0-9677745)	614938 (0-9930498)	437 (0-782329)	1221396 (0-33566870)	2488046841 (0-3964807200)	2489248708 (0-3998549353)
**PZ5**	82167 (0-1399180)	229 (0-163330)	154 (0-633839)	81081 (0-6498653)	0 (0-0)	392459 (0-870835)	2038 (0-8287440)	616948 (0-9974524)	500 (0-447826)	1222860 (0-28696686)	2469061398 (0-3585527787)	2470287178 (0-3615919826)
**SZ15**	82010 (0-1573046)	250 (0-199805)	162 (0-722068)	81081 (0-7883113)	0 (0-0)	392459 (0-912202)	2070 (0-9373521)	622088 (0-10244507)	500 (0-610902)	1230310 (0-32103760)	2488444026 (0-4008961618)	2489750505 (0-4050482510)
**CH**	78797 (0-1233940)	216 (0-215876)	150 (0-772354)	81081 (0-9293925)	0 (0-0)	371245 (0-912732)	1863 (0-10011133)	616772 (0-12172636)	437 (0-677743)	1197070 (0-34955077)	2469061398 (0-3578649482)	2470236232 (0-3617888971)
**PV1km14d**	80751 (0-1307968)	216 (0-122149)	150 (0-428933)	81081 (0-4510138)	0 (0-401338)	392459 (0-784918)	1863 (0-5606373)	612697 (0-7813029)	437 (0-415310)	1214560 (0-20856185)	2468823087 (0-3421632794)	2470032286 (0-3439796894)
**PV1km25IH**	81471 (0-1291881)	223 (0-146121)	150 (0-566299)	81081 (0-5760815)	0 (0-0)	392459 (0-827876)	1863 (0-6770117)	616123 (0-8153202)	437 (0-442742)	1223164 (0-23220039)	2469101116 (0-3479837203)	2470319723 (0-3499932384)
**PV1km25PC**	80592 (0-1353511)	225 (0-143048)	150 (0-548939)	81081 (0-5639193)	0 (0-0)	392459 (0-796055)	1863 (0-6505056)	616757 (0-7951946)	468 (0-443170)	1224268 (0-23236018)	2469061398 (0-3478651853)	2470280527 (0-3498165169)
**PV3km14d_bov**	81180 (0-1155745)	216 (0-121055)	144 (0-421179)	81081 (0-4301354)	0 (0-138713)	392459 (0-743020)	1863 (0-5357201)	614350 (0-6741922)	437 (0-344157)	1220026 (0-19410937)	2469021680 (0-3278017141)	2470241812 (0-3297428078)
**PV3km14d_sui**	81624 (0-1320249)	225 (0-155464)	154 (0-575687)	81081 (0-5647301)	0 (0-393821)	392459 (0-797116)	1863 (0-7374548)	615474 (0-8439957)	500 (0-490644)	1217546 (0-26462682)	2468902524 (0-3466886014)	2470126515 (0-3493963246)
**PV3km14d_ovi**	81366 (0-1461162)	225 (0-183576)	161 (0-685882)	81081 (0-6510814)	0 (0-67292)	392459 (0-870304)	2070 (0-9177441)	619807 (0-9768516)	500 (0-535126)	1231644 (0-28869925)	2469021680 (0-3557707615)	2470299443 (0-3578709031)
**SV3km14d**	81064 (0-1208902)	240 (0-150975)	154 (0-521590)	81081 (0-5517571)	0 (0-244709)	392459 (0-806132)	2070 (0-6312580)	618377 (0-18387796)	500 (0-4613214)	1225610 (0-36147945)	2469061398 (0-3361563822)	2470291595 (0-3391992137)
**SV3km25IH**	79958 (0-1252796)	216 (0-130728)	146 (0-463218)	81081 (0-4429057)	0 (0-0)	392459 (0-743551)	1863 (0-6141009)	613632 (0-7070693)	437 (0-344824)	1221805 (0-21244700)	2468902524 (0-3336131186)	2470175328 (0-3365622625)
**SV3km14d_bov**	78889 (0-1098984)	216 (0-137411)	144 (0-426132)	81081 (0-4504057)	0 (0-37367)	371245 (0-689985)	1863 (0-4951752)	613869 (0-11362791)	437 (0-1049450)	1190392 (0-23009439)	2488046841 (0-3482438870)	2489231508 (0-3500761198)
**South_Ruminant (SZ 1)**												
**Basic**	92620 (0-1458735)	350 (0-172345)	234 (0-648141)	81081 (0-5841896)	0 (0-0)	392459 (0-827346)	4689 (0-8208611)	731268 (0-7991678)	687 (0-574105)	1391152 (0-25657513)	2472556626 (0-3489257376)	2473997748 (0-3515668614)
**DP15**	93291 (0-1567549)	350 (0-310005)	250 (0-1068196)	81081 (0-12774332)	0 (0-0)	392459 (0-892049)	4729 (0-13665166)	743838 (0-10575895)	687 (0-1228397)	1390628 (0-43280974)	2472636063 (0-3800366136)	2474041290 (0-3843561615)
**DP15SZ15**	94427 (0-1542822)	325 (0-290001)	234 (0-1055614)	81081 (0-11977709)	0 (0-0)	392459 (0-880911)	4872 (0-13398217)	741317 (0-10086636)	687 (0-1252177)	1389146 (0-41235421)	2496665756 (0-4142405056)	2497999380 (0-4174048328)
**PZ5**	90429 (0-1487987)	324 (0-181062)	217 (0-731113)	81081 (0-6851356)	0 (0-0)	392459 (0-850681)	4342 (0-9653890)	744758 (0-9936915)	624 (0-543880)	1383376 (0-28057102)	2472477189 (0-3582383740)	2473878112 (0-3608413263)
**SZ15**	96016 (0-1917323)	381 (0-297818)	279 (0-1060989)	81081 (0-10631764)	0 (0-0)	392459 (0-1028879)	5722 (0-13408633)	745673 (0-13142091)	780 (0-850993)	1395642 (0-43478034)	2496824630 (0-4347180513)	2498269765 (0-4393579914)
**CH**	92932 (0-1495469)	360 (0-299719)	253 (0-1118914)	81081 (0-10548655)	0 (0-0)	371245 (0-1018272)	5230 (0-14476207)	744877 (0-14089412)	749 (0-831346)	1365122 (0-42621133)	2472794937 (0-3887240290)	2474119872 (0-3944161311)
**PV1km14d**	95496 (0-1560442)	360 (0-226983)	250 (0-900125)	81081 (0-7303383)	0 (0-678190)	392459 (0-913263)	5020 (0-11167377)	741284 (0-10824752)	749 (0-653806)	1395760 (0-34456566)	2472715500 (0-3635951070)	2474120304 (0-3668106801)

**PV1km25IH**	94614 (0-1847319)	394 (0-235913)	288 (0-861066)	81081 (0-9127709)	0 (0-1168135)	392459 (0-1019333)	5722 (0-11993437)	741972 (0-12414257)	812 (0-643294)	1399480 (0-38668866)	2472794937 (0-3920473472)	2474204660 (0-3967274606)
**PV1km25PC**	94641 (0-1624709)	359 (0-250650)	270 (0-962124)	81081 (0-8444600)	0 (0-0)	392459 (0-965767)	5549 (0-12098428)	744483 (0-11570038)	749 (0-686128)	1386110 (0-37328173)	2472715500 (0-3722623794)	2474074180 (0-3761245383)
**PV3km14d_bov**	93483 (0-1664166)	324 (0-213903)	234 (0-838593)	81081 (0-7952032)	0 (0-339479)	392459 (0-965767)	4505 (0-11066287)	740131 (0-11318712)	687 (0-628990)	1381406 (0-34864004)	2472556626 (0-3710200239)	2473940324 (0-3744594391)
**PV3km14d_sui**	92642 (0-1574525)	324 (0-225158)	225 (0-824822)	81081 (0-8355411)	0 (0-520533)	392459 (0-933946)	4347 (0-10944368)	739545 (0-10977615)	624 (0-575607)	1377100 (0-34623557)	2472556626 (0-3707120757)	2474016530 (0-3739231215)
**PV3km14d_ovi**	94126 (0-1641050)	324 (0-215947)	234 (0-820024)	81081 (0-9170276)	0 (0-103484)	392459 (0-965237)	4342 (0-10786188)	741907 (0-11903762)	687 (0-706293)	1381602 (0-34342504)	2472556626 (0-3727823702)	2474005628 (0-3777798706)
**SV3km14d**	90564 (0-1540267)	324 (0-265373)	234 (0-916147)	81081 (0-9206763)	0 (0-456434)	392459 (0-1028879)	4816 (0-12362277)	736894 (0-33894811)	687 (0-11185619)	1378604 (0-68892233)	2472556626 (0-3739284771)	2473934554 (0-3790567834)
**SV3km25IH**	93811 (0-1542124)	324 (0-204724)	230 (0-835754)	81081 (0-7751356)	0 (0-0)	392459 (0-901595)	4474 (0-11077136)	742074 (0-11215749)	687 (0-842253)	1383797 (0-34979958)	2472556626 (0-3583948218)	2474009668 (0-3668274238)
**SV3km14d_bov**	94807 (0-1551794)	377 (0-218685)	279 (0-822111)	81081 (0-7866897)	0 (0-66406)	371245 (0-880381)	6245 (0-10718913)	751176 (0-21540271)	812 (0-2032983)	1386610 (0-45231869)	2497102659 (0-4008982334)	2498444543 (0-4060845114)
**Zealand_Ruminant (SZ 1)**												
**Basic**	46548 (0-201286)	216 (0-9088)	126 (0-24170)	81081 (0-648649)	0 (0-0)	392459 (0-456101)	1449 (0-239674)	482744 (0-812062)	375 (0-22152)	1042586 (0-2292719)	2467075473 (0-2733630332)	2468143187 (0-2735919895)
**DP15**	45859 (0-160853)	216 (0-10769)	126 (0-10769)	81081 (0-608108)	0 (0-0)	392459 (0-456101)	1449 (0-163752)	483646 (0-834296)	375 (0-13531)	1040516 (0-2132682)	2467075473 (0-2704367129)	2468118540 (0-2705789792)
**DP15SZ15**	45215 (0-185284)	216 (0-8575)	126 (0-20519)	81081 (0-652703)	0 (0-0)	392459 (0-456101)	1449 (0-241293)	488927 (0-998752)	375 (0-26103)	1045706 (0-2305608)	2484789924 (0-2762020071)	2485895437 (0-2765045167)
**PZ5**	46071 (0-174883)	216 (0-7567)	126 (0-13843)	81081 (0-527027)	0 (0-0)	392459 (0-445494)	1449 (0-145246)	484857 (0-896116)	375 (0-16750)	1041813 (0-2177681)	2466996036 (0-2709749224)	2467997388 (0-2713448772)
**SZ15**	45117 (0-175599)	216 (0-9712)	126 (0-20343)	81081 (0-648649)	0 (0-0)	392459 (0-445494)	1449 (0-238147)	483829 (0-783143)	375 (0-26221)	1036376 (0-2147437)	2484233865 (0-2726412307)	2485286879 (0-2728260475)
**CH**	46068 (0-147350)	216 (0-7206)	126 (0-16824)	81081 (0-648649)	0 (0-0)	371245 (0-413673)	1449 (0-178832)	486695 (0-899588)	375 (0-16418)	1021039 (0-2038013)	2467075473 (0-2670963939)	2468083330 (0-2672795633)
**PV1km14d**	46133 (0-162532)	216 (0-7889)	126 (0-22705)	81081 (0-648649)	0 (0-0)	392459 (0-456101)	1449 (0-232015)	484000 (0-877511)	375 (0-20455)	1038696 (0-2211233)	2467075473 (0-2733400015)	2468098215 (0-2735515076)
**PV1km25IH**	46533 (0-162310)	216 (0-7637)	126 (0-17214)	81081 (0-527027)	0 (0-0)	392459 (0-456101)	1449 (0-175790)	479698 (0-877901)	375 (0-15910)	1040046 (0-2144181)	2466996036 (0-2716247972)	2468013600 (0-2717527489)
**PV1km25PC**	45397 (0-177910)	216 (0-11462)	126 (0-25165)	81081 (0-648649)	0 (0-0)	392459 (0-456101)	1449 (0-246476)	482654 (0-824501)	375 (0-48262)	1040897 (0-2166850)	2467075473 (0-2720989010)	2468122110 (0-2723723493)
**PV3km14d_bov**	46694 (0-169904)	216 (0-7648)	126 (0-19364)	81081 (0-648649)	0 (0-0)	392459 (0-456101)	1449 (0-231838)	483064 (0-788544)	375 (0-20121)	1039578 (0-2318828)	2467075473 (0-2719941723)	2468120686 (0-2722726986)
**PV3km14d_sui**	45177 (0-165722)	216 (0-9741)	125 (0-19368)	81081 (0-450000)	0 (0-0)	392459 (0-456101)	1449 (0-178989)	482086 (0-800388)	375 (0-27492)	1042564 (0-1887341)	2466996036 (0-2722905416)	2468052654 (0-2724341337)
**PV3km14d_ovi**	46269 (0-182306)	216 (0-10929)	126 (0-24797)	81081 (0-648649)	0 (0-0)	392459 (0-466708)	1449 (0-323259)	485428 (0-825078)	375 (0-28926)	1045162 (0-2318086)	2467115192 (0-2746993778)	2468204681 (0-2751039777)
**SV3km14d**	46522 (0-195106)	216 (0-11807)	126 (0-31227)	81081 (0-729730)	0 (0-0)	392459 (0-456101)	1449 (0-349687)	485571 (0-971076)	406 (0-35191)	1047756 (0-2686797)	2467154910 (0-2745189688)	2468217014 (0-2747465438)
**SV3km25IH**	46604 (0-182879)	216 (0-9080)	126 (0-21988)	81081 (0-648649)	0 (0-0)	392459 (0-456101)	1449 (0-235772)	486069 (0-814285)	375 (0-22781)	1037558 (0-2237868)	2467075473 (0-2747080753)	2468090821 (0-2749523153)

**SV3km14d_bov**	45201 (0-165410)	216 (0-8499)	126 (0-19211)	81081 (0-527027)	0 (0-0)	371245 (0-424810)	1449 (0-182297)	484164 (0-781315)	375 (0-27650)	1021838 (0-1971836)	2484511894 (0-2721566974)	2485525882 (0-2723118419)
**Entire_DK_Ruminant (SZ 2)**												
**Basic**	63088 (13637-807645)	4784 (1548-82814)	3310 (800-186099)	162162 (81081-2312839)	0 (0-0)	392459 (381852-625813)	41479 (9506-230846)	682686 (258543-3664931)	8866 (2747-205109)	1459531 (819159-9897155)	2474304240 (2459370084-3138473306)	2475718042 (2460230234-3144927294)
**DP15**	60839 (11792-839978)	4716 (1572-96188)	3303 (838-240838)	162162 (81081-2432434)	0 (0-0)	371245 (360638-584446)	40683 (9618-294983)	693995 (258932-3456229)	8679 (2810-255288)	1448001 (799322-11110965)	2473986492 (2459842734-3136724837)	2475479224 (2460630285-3151807821)
**DP15SZ15**	61477 (11832-732156)	4464 (1548-82814)	3265 (781-224033)	162162 (81081-2031082)	0 (0-0)	371245 (360638-562171)	39475 (9294-2726340)	691660 (248254-3124456)	8323 (2685-216913)	1445279 (808991-9665559)	2498453088 (2468664213-3181180339)	2499739095 (2469467427-3188390185)
**PZ5**	62041 (14866-787932)	4716 (1548-84262)	3240 (809-207452)	162162 (81081-2316893)	0 (0-0)	403066 (381852-625813)	39484 (9315-2646702)	727936 (263202-3298964)	8679 (2806-216254)	1491044 (849152-11170193)	2493555793 (2460085017-3140683941)	2494704039 (246090514-3151784538)
**SZ15**	64685 (13190-714063)	4628 (1548-69448)	3437 (774-178553)	162162 (81081-2191217)	0 (0-0)	392459 (381852-583385)	42907 (8901-2296009)	691924 (237978-3141563)	8679 (2685-189411)	1500274 (823793-9262379)	2501392257 (2468477536-3181533102)	2502914117 (2469256972-3184874729)
**CH**	57598 (10938-727366)	4959 (1575-101961)	3310 (828-281314)	162162 (81081-3652705)	0 (0-0)	371245 (360638-668241)	41479 (9613-3564408)	718133 (245182-5889204)	9085 (2810-265195)	1490234 (772818-1494306)	2501301342 (2459528958-3166968232)	2502828780 (2460300371-3180666831)
**PV1km14d**	63184 (13645-845457)	4248 (1548-83513)	3370 (810-212836)	162162 (81081-2519596)	0 (0-141406)	403066 (381852-615206)	43020 (9506-2779874)	721671 (293152-3604912)	8148 (2747-222298)	1502799 (853437-11122928)	2474780862 (2460077073-3129483045)	2476194958 (2460887529-3137564215)
**PV1km25IH**	64950 (13403-1007264)	4701 (1548-120580)	3500 (810-361845)	162162 (81081-3445948)	0 (0-0)	403066 (381852-710669)	44192 (9506-4467486)	718017 (244592-5354382)	8679 (2806-278757)	1522051 (822470-16246717)	2476925661 (2459846706-3238271184)	2478473906 (2460671292-3254169383)
**PV1km25PC**	62394 (12747-936959)	5004 (1575-82647)	3600 (810-234398)	162162 (81081-3164191)	0 (0-0)	403066 (381852-647027)	43056 (9315-3079941)	724631 (247637-4320364)	9335 (2810-216011)	1507627 (830246-12954394)	2476528476 (2459528958-3132691947)	2477965482 (2460306544-3144659668)
**PV3km14d_bov**	65207 (13930-658081)	4649 (1548-81861)	3240 (781-194719)	162162 (81081-2152704)	0 (0-9933)	403066 (381852-593992)	39484 (9294-2478477)	710469 (260833-3055724)	8648 (2685-194256)	1505982 (818285-9545489)	2476766787 (2459846706-3085114211)	2478204654 (2460678128-3092837947)
**PV3km14d_sui**	63675 (15881-958385)	4611 (1548-99289)	3552 (781-249303)	162162 (81081-3000002)	0 (0-183360)	397762 (381852-636950)	44778 (9315-3184985)	697339 (246821-4184345)	8679 (2685-243665)	1495187 (827535-12904571)	2476766787 (2460077073-3150308707)	2478200946 (2460886306-3163564430)
**PV3km14d_ovi**	63427 (14768-724635)	4464 (1548-82008)	2916 (800-176003)	162162 (81081-2029055)	0 (0-1932)	392459 (381852-583385)	36225 (9294-2202728)	696913 (267057-2760084)	8055 (2747-185752)	1469168 (878833-9608174)	2475257484 (2460720513-3062069460)	2476705764 (2461873663-3070889196)
**SV3km14d**	67685 (13004-703990)	4428 (1572-82048)	3310 (816-214882)	162162 (81081-2152704)	0 (0-66920)	403066 (381852-615206)	41336 (9521-2732441)	699355 (242978-6416574)	8385 (2810-857320)	1518416 (827738-15512327)	2503157047 (2459767269-307358469)	2504331448 (2460559163-3087553853)
**SV3km25IH**	63404 (12699-774538)	4000 (1548-82008)	2880 (781-205905)	162162 (81081-2233785)	0 (0-0)	403066 (381852-573838)	35354 (9315-2662036)	693771 (244111-3250068)	7524 (2685-194715)	1496273 (821302-10421606)	2476528476 (2459846706-3083677409)	2477926156 (2460678142-3092586477)
**SV3km14d_bov**	62818 (12918-821038)	4716 (1620-81854)	3402 (846-216019)	162162 (81081-2312839)	0 (0-6895)	371245 (360638-583915)	41845 (9936-272861)	690475 (247764-4636693)	9179 (2931-271739)	1441573 (806031-11701296)	2500995072 (2468664213-3217715431)	2502230644 (2469612655-3226794023)

References

[3] Bradhurst, R.; Garner, G.; Hóvári, M.; de la Puente, M.; Mintiens, K.; Yadav, S.; Federici, T.; Kopacka, I.; Stockreiter, S.; Kuzmanova, I.; et al. Development of a transboundary model of livestock disease in Europe. *Transbound. Emerg. Dis.* **2021**, *69*, 1963–1982. https://doi.org/10.1111/tbed.14201.

[8] Garner, G.; Vosloo, W.; Tapsuwan, S.; Bradhurst, R.; Seitzinger, A.H.; Breed, A.C.; Capon, T. Comparing surveillance approaches to support regaining free status after a foot-and-mouth disease outbreak. *Prev. Vet. Med.* **2021**, *194*, 105441. https://doi.org/10.1016/j.prevetmed.2021.105441.

[53] Brito, B. P., Perez, A. M., Cosentino, B., Rodriguez, L. L., König, G. A. Factors associated with within-herd transmission of serotype A foot-and-mouth disease virus in cattle, during the 2001 outbreak in Argentina: A protective effect of vaccination. *Transbound. Emerg. Dis.* **2011**. *58*, 387–393. doi:10.1111/j.1865-1682.2011.01217.x

[54] Carpenter, T.E., Thurmond, M.C., Bates, T.W. A simulation model of intra herd transmission of foot and mouth disease with reference to disease spread before and after clinical diagnosis. *J. Vet. Diagn. Investig.* **2004**, *16*, 11–16.doi:10.1177/104063870401600103

[55] Goris, N. E., Eblé, P. L., de Jong, M. C. and De Clercq, K. Quantification of foot-and-mouth disease virus transmission rates using published data. *Altex*, **2009**, *26*, 52–54.pmid:19326033

[56] European Union., 2003. Council Directive 2003/85/EC. Off J L 306. 2003. Available online at: https://eur-lex.europa.eu/legal-content/EN/TXT/HTML/?uri=CELEX:32003L0085&from=EN(Accessed on 01.08.2022).

[57] Orsel, K., Dekker, A., Bouma, A., Stegeman, J.A., De Jong, M.C.M., 2005. Vaccination against foot and mouth disease reduces virus transmission in groups of calves. *Vaccine*, **2005**, *23*, 4887-4894. doi:10.1016/j.vaccine.2005.05.014

[58] Orsel, K., De Jong, M.C.M., Bouma, A., Stegeman, J. A., Dekker, A. The effect of vaccination on foot and mouth disease virus transmission among dairy cows. *Vaccine*, **2007a,** *25*, 327–335. doi:10.1016/j.vaccine.2006.07.030

[59] Orsel, K., De Jong, M.C.M., Bouma, A., Stegeman, J. A., Dekker, A. Foot and mouth disease virus transmission among vaccinated pigs after exposure to virus shedding pigs. *Vaccine*, **2007b**. *25*, 6381–6391. doi:10.1016/j.vaccine.2007.06.010

[60] Orsel, K., Bouma, A., Dekker, A., Stegeman, J. A., De Jong, M.C.M. Foot and mouth disease virus transmission during the incubation period of the disease in piglets, lambs, calves, and dairy cows. Preventive Veterinary Medicine, **2009,** *88*, 158–163.doi:10.1016/j.prevetmed.2008.09.001
